# B cells expressing mutated IGHV1-69–encoded antigen receptors related to virus neutralization show lymphoma-like transcriptomes in patients with chronic HCV infection

**DOI:** 10.1097/HC9.0000000000000503

**Published:** 2024-07-31

**Authors:** Christoph Schultheiß, Edith Willscher, Lisa Paschold, Christin Ackermann, Moritz Escher, Rebekka Scholz, Maximilian Knapp, Jana Lützkendorf, Lutz P. Müller, Julian Schulze zur Wiesch, Mascha Binder

**Affiliations:** 1 Divison of Medical Oncology, University Hospital Basel, Basel, Switzerland; 2 Laboratory of Translational Immuno-Oncology, Department of Biomedicine, University of Basel and University Hospital of Basel, Basel, Switzerland; 3 Internal Medicine IV, Department of Hematology/Oncology, Martin-Luther-University Halle-Wittenberg, Halle (Saale), Germany; 4 Infectious Disease Unit, I, Department of Medicine, University Medical Center Hamburg-Eppendorf, Hamburg, Germany

## Abstract

**Background:**

Chronic HCV infection leads to a complex interplay with adaptive immune cells that may result in B cell dyscrasias like cryoglobulinemia or lymphoma. While direct-acting antiviral therapy has decreased the incidence of severe liver damage, its effect on extrahepatic HCV manifestations such as B cell dyscrasias is still unclear.

**Methods:**

We sequenced B cell receptor (BCR) repertoires in patients with chronic HCV mono-infection and patients with HCV with a sustained virological response (SVR) after direct-acting antiviral therapy. This data set was mined for highly neutralizing HCV antibodies and compared to a diffuse large B cell lymphoma data set. The TKO model was used to test the signaling strength of selected B-BCRs in vitro. Single-cell RNA sequencing of chronic HCV and HCV SVR samples was performed to analyze the transcriptome of B cells with HCV-neutralizing antigen receptors.

**Results:**

We identified a B cell fingerprint with high richness and somatic hypermutation in patients with chronic HCV and SVR. Convergence to specific immunoglobulin genes produced high-connectivity complementarity-determining region 3 networks. In addition, we observed that IGHV1-69 CDR1 and FR3 mutations characterizing highly neutralizing HCV antibodies corresponded to recurrent point mutations found in clonotypic BCRs of high-grade lymphomas. These BCRs did not show autonomous signaling but a lower activation threshold in an in vitro cell model for the assessment of BCR signaling strength. Single-cell RNA sequencing revealed that B cells carrying these point mutations showed a persisting oncogenic transcriptome signature with dysregulation in signaling nodes such as CARD11, MALT1, RelB, MAPK, and NFAT.

**Conclusions:**

We provide evidence that lymphoma-like cells derive from the anti-HCV immune response. In many patients, these cells persist for years after SVR and can be interpreted as a mechanistic basis for HCV-related B cell dyscrasias and increased lymphoma risk even beyond viral elimination.

## Graphical abstract

**Figure:**
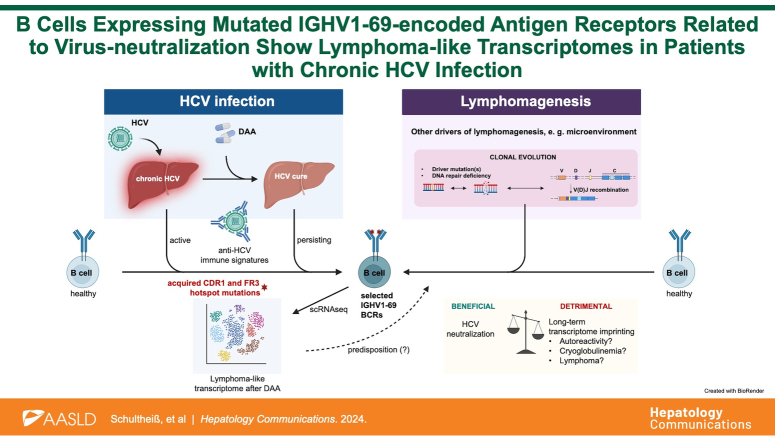

## INTRODUCTION

The HCV is an enveloped single-stranded RNA virus that can cause chronic disease courses.^1^,^2^ According to the WHO, an estimated 58 million people have chronic HCV infection worldwide, with about 1.5 million new infections per year. Chronic HCV infection can cause severe liver disease and result in liver cirrhosis with the risk of developing HCC and a variety of clinically important extrahepatic B cell dyscrasias such as mixed cryoglobulinemia or—rarely—lymphoma.^3^,^4^ The chronic course of the disease is generally attributed to a number of host and viral factors.^5^,^6^ Importantly, HCV RNA replicates inside infected host cells at high levels with a high mutation rate constantly generating quasispecies that evade efficient humoral and cellular immunity.^7^ Exhausted HCV-specific CD8^+^ T cells comprise memory-like and terminally exhausted subsets that are maintained even after viral elimination.^8^,^9^ The complex interplay of HCV with the adaptive immune system is also showcased by the evident B cell dyscrasia observed in this disease. Type II mixed cryoglobulinemia results from the production of a monoclonal rheumatoid factor–like IgM autoantibody binding IgG by HCV B cells. These antibodies are found in up to 50% of patients with chronic HCV, but the circulating immune complexes result in clinically detectable symptomatic arthritis and vasculitis in only 5%–10% of patients.^10^ Analysis of these antibodies revealed similar sequences, often favoring the use of specific immunoglobulin heavy chain (HC) variable genes (IGHV) such as IGHV1‐69, IGHV3-23, IGHV3-7, and IGHV4-59.^11^ Interestingly, many HCV-dependent or HCV-independent B cell non-Hodgkin lymphomas use the same IGHV genes in their clonotypic B cell receptor (BCR).^11^ Therefore, chronic active BCR-mediated clonal selection was hypothesized to cause both the expansion of autoreactive B cells and drive lymphomagenesis at least in some patients with HCV.^11^ Yet, it remains unclear whether the BCR expressed by the lymphoma cell directly (cross-)reacts with HCV antigens, what consequences may arise from such an interaction, or if the mechanisms underlying clonal B cell selection may be more indirect.^12^,^13^

Today, chronic HCV is treatable through direct-acting antiviral (DAA) therapy administered over several weeks.^1^,^14^,^15^ The introduction of DAA treatment has largely transformed our ability to encounter the global threat caused by HCV. Nevertheless, while greatly reducing the burden of severe liver damage and its clinical consequences, the effect of viral elimination on extrahepatic manifestations of HCV, such as the accompanying B cell dyscrasia, remains to be defined.

Here, we dissect HCV imprinting of B cell repertoires in patients with chronic HCV mono-infection and patients with HCV with a sustained virological response (SVR) after HCV therapy. We show that complementarity-determining region 1 (CDR1) and framework region 3 mutations characterizing highly neutralizing HCV antibodies correspond to recurrent point mutations found in the clonotypic BCRs of HCV-unrelated high-grade lymphomas. Using single-cell sequencing, we demonstrate that these point mutations confer a persisting oncogenic signature on B cells that can be interpreted as a mechanistic basis for HCV-related B cell dyscrasias and that highlights the elevated lymphoma risk in these patients even years after HCV cure.

## METHODS

### Biological samples

Peripheral blood of 52 patients with HCV (30 with chronic HCV, 29 with SVR; 7 paired samples with matched time points) was collected in in BD Vacutainer CPT™ tubes between 2010 and 2019 during routine clinical monitoring at the University Medical Center Hamburg-Eppendorf. As a control, the blood of 12 healthy donors (HDs) without any hematological abnormalities was collected (University Medical Center Halle). Written informed consent was obtained from each patient, and HDs were included for the use of their biological material as approved by the respective Ethics Commission Hamburg (Ethikkommission der Ärztekammer Hamburg, project number PV4081, Ethikkommission Halle 2020-039 and 2014/75) and in line with the Helsinki Declaration of 1975, as revised in 2000. Peripheral blood mononuclear cells (PBMCs) were isolated by density gradient centrifugation and cryopreserved in liquid nitrogen. Bone marrow was aspirated from a patient in preparation for allogeneic stem cell donation at the University Hospital Halle (Saale). The subcohort of patients with chronic HCV was previously used as a control group in a report on autoimmune hepatitis.^16^

### Next-generation immunosequencing and data processing

Bulk immunosequencing of B cell repertoires was performed from 500 ng of PBMC or bone marrow–derived genomic DNA as described.^16^,^17^,^18^ In brief, V(D)J rearranged IGH loci were amplified in multiplex PCR using the BIOMED2-FR1 primer set, pooled at 4 nM, and quality-assessed on a 2100 Bioanalyzer (Agilent Technologies). Sequencing was performed on an Illumina MiSeq (paired-end, 2×301 cycles, v3 chemistry). Rearranged IGH loci were annotated using MiXCR v3.0.13 and the IMGT 202011-3.sv6 IGH library as reference. Nonproductive reads containing stop codons and sequences that were not covered by at least 2 read counts were discarded. Each unique complementarity-determining region 3 (CDR3) amino acid sequence was considered a clone. All samples were proportionally normalized to a read depth of 30,000 to avoid repertoire biases due to varying sequencing depth. Broad repertoire metrics (clonality, diversity, and richness) were analyzed as described.^17^ IGHV genes were regarded as somatically hypermutated if they showed <98% identity to the germline sequence. Principal component analysis of V-J Gene combinations, their contributions, and the Pillai *p* value were calculated using R package ade4.

### Network connectivity analysis

Network connectivity of all IGH clonotypes in a given repertoire was calculated with the imnet tool (https://github.com/rokroskar/imnet) and visualized as petri dish plots with R library igraph. CDR3 sequences (=nodes in the petri dish plot) were calculated as connected (=edges in the petri dish plot) if they differed in a maximum of 3 positions (Levenshtein distance ≤3). The connectivity index β was calculated by dividing the number of links by the number of sequences. Data analysis and plotting were performed using the R version (v4.1.2).

### Sequence alignment of IGHV1-69

Raw reads from the bulk immunosequencing analysis were aligned to germline sequences using igblast.^19^ Sequences were filtered for productive and complete IGHV1-69 V(D)J regions. Any amino acid alteration with respect to the IGHV1-69*01-IGHV1-69*20 germline alleles was counted as a mutation.

### Single-cell RNA and BCR sequencing and data processing

Single-cell RNA and BCR sequencing as described.^20^ CD19^+^ cells B cells were FACS-sorted (CD19-PECy7, clone J3-119, Beckman Coulter) from cryopreserved PBMCs of 7 patients with HCV and processed on a 10X Chromium Controller (10X Genomics). Single-cell libraries were generated using the Next GEM Single Cell 5ʹ Kit v2 and Chromium Single Cell Human BCR Amplification kits. The libraries were sequenced on an Illumina NovaSeq 6000 system (S4 flow cell) with 150 base pairs and paired-end configurations. Filtered reads were aligned, and the barcodes were counted with the cellranger count pipeline (v 5.0.1). For quality control purposes, samples were first integrated with single-cell RNA sequencing data of a previously published healthy individual^20^ in R (v 4.2.1) using the package Seurat (v 4.1.1). This step was implemented to rule out run-based cluster artifacts. After quality control, HCV-related samples were integrated separately. Integration anchors were calculated using the function *FindIntegrationAnchors,* and data sets were integrated with *IntegrateData* to one object. After scaling, principal component analysis and uniform manifold approximation and projection calculations were performed on 20 dimensions. Clusters that were not covered by each individual were discarded. B cell clusters were assigned according to subset markers found with the function *FindAllMarkers* and selected B cell population marker genes. For cluster assignment, IGHV, IGKV, and IGLV genes were eliminated from genelists. Fastq files from V(D)J libraries were analyzed with the cellranger vdj pipeline, and the filtered results were integrated with GEX Data with package scRepertoire (v 1.4.0). Gene module scores were calculated in Seurat with the function *AddModuleScore*. For further analysis of B cells with IGHV1-69–encoded antigen receptor including Potential of Heat-diffusion for Affinity-based Transition Embedding (PHATE),^21^ we re-structured the data set using the function *subset* to selectively obtain B cells from HCV with BCRs encoded by a wild type or mutant IGHV1-69 gene as well as all HD cells. We used package SCISSORS (v.1.2) to wrap results from PHATE^21^ into the Seurat object. After *RunPHATE* was performed on 20 principal components and Euclidean distance, a *DimPlot* was generated on PHATE reduction. Differentially expressed genes were detected with the function *FindSpecificMarkers* and visualized with the package EnhancedVolcano (v 1.14.0). Upregulated genes were used as input for pathway analysis with package enrichR (v.3.0).

### Cloning and cellular expression of IGHV1-69 BCR

We used the triple knockout (TKO) cell model to assess the autonomous signaling capacity of selected BCRs from patients with ABC diffuse large B cell lymphoma (DLBCL). TKO cells are pre-B cells without functional BCRs due to the knockout of *RAG2*, *λ5*, and *SLP-65* (*BLNK*).^22^ Retroviral transduction of BCR HC and light chain (LC) sequences reconstitutes functional BCRs that can induce Ca^2+^ flux with or without antigen. The introduced estrogen receptor (ERT2)-inducible SLP-65 variant restricts BCR signaling to the presence of 4-hydroxytamoxifen (OHT).^22^ HC and LC sequences (Supplemental Table S1, http://links.lww.com/HC9/A999) were synthesized through GeneArt (ThermoFisher). HCs encompassed the completely rearranged V(D)J sequence without the constant region. HCs were cloned in-frame to the adjacent murine µ constant chain in the pMIZCC vector through *Xho*I and *Psh*AI. LCs encompassed the respective kappa or lambda constant region and were cloned into the pMIZYN vector through *Xho*I and *Eco*RI. We used the second-generation retrovirus producer cell line Phoenix-eco to produce helper-free retroviral particles containing the HC and LC sequences for TKO transduction. To produce viral supernatant, Phoenix-eco cells were seeded at 0.5×10^6^ cells/well in 6-well plates 24 hours before transfection and transfected with 1 µg of DNA per plasmid along with 3 µg PEI MAX (Polysciences 24765-100) per well. The DNA mix consisted of 3 plasmids in a 1:1:1 ratio (pMIZYN containing the human variable light and constant chain, pMIZCC encoding the HC human variable region fused to murine µ constant region and the pKAT packaging plasmid to increase virus production). Viral supernatant was collected after 48 hours of incubation at 37°C, filtered through a 0.45 µm filter, and concentrated using the Lenti-X Concentrator (Clontech 631231) overnight. Transductions were performed by resuspending 2.5×10^5^ TKO cells in TKO medium containing 10 µg/mL polybrene (Merck Millipore TR-1003-G) and viral supernatant followed by 3-hour spinoculation at 37°C and 300*g*.

### Calcium signaling

Quantification of intracellular Ca^2+^ release from the endoplasmic reticulum was used as a proxy for BCR signaling. For calcium flux measurements, 1.5×10^6^ TKO cells were loaded with the fluorescent Ca^2+^ indicator Indo-1 AM (ThermoFisher I1223) using the nonionic, surfactant polyol Pluronic F-127 (Invitrogen P3000MP) and stimulated with either 2 µM 4-OHT (Merck H7904) for measuring of autonomous signaling capabilities by induction of SLP-65-ERT2 or 2 µM 4-OHT + 10 µg/mL anti-IgM (Sigma-Aldrich AP500 Goat Anti-Mouse IgM) for BCR crosslinking. As a loading control to achieve maximum cytosolic calcium levels, 4 µg/mL Ionomycin (StemCell Technology 73722) was added. All measurements were performed at 37°C using a custom 3D-printed tube holder connected to a water pump and heating unit on a BD LSR Fortessa flow cytometer.

### Statistical analysis

We used ordinary 1-way ANOVA followed by post-hoc testing (Tukey multiple comparisons test) to assess differences in immune repertoire metrics between groups. To test for the difference in the mean connectivity in B cell networks, we used the 2-tailed Mann-Whitney test. Both tests were performed using GraphPad Prism 8.3.1 (GraphPad Software).

## RESULTS

### HCV B cell repertoires show a high richness and somatic hypermutation independently of disease activity

**FIGURE 1:**
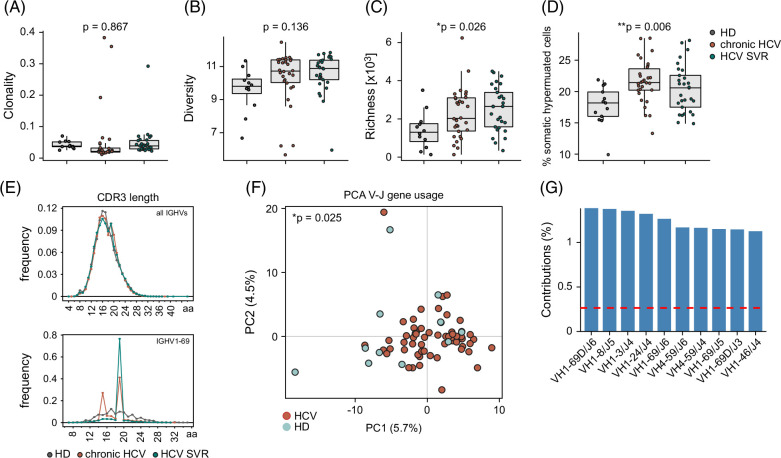
HCV-induced immune repertoire metrics of peripheral blood B cells. Clonality (A), diversity (B), and richness (C) were compared between patients with chronic HCV (n = 30), with SVR (n = 29) and HDs (n = 12). (D) Frequency of B cell clones with somatic hypermutation (<98% identity to germlines) in the immune repertoires of patients with HCV and HD. Statistics: 1-way ANOVA. (E) Mean frequency of CDR3 lengths per group in aa. (F) PCA for comparison of IGHV-J rearrangements in the immune repertoires of all patients with HCV (n = 42) and HDs (n = 12). Statistics: Pillai-Bartlett test of MANOVA of all PCs. The first 2 PCs are shown. (G) The contributing V-J gene combinations of the first 2 PCs are shown in percent. The dotted red line indicates the expected value if the contributions were uniform. Abbreviations: aa, amino acid; HD, healthy donor; PC, principal components; PCA, principal component analysis; SVR, sustained virological response.

We performed peripheral blood next-generation IGHV(D)J sequencing to determine general immune repertoire metrics in a real-world cohort of patients with chronic HCV. Table 1 summarizes the demographic, clinical, and serological characteristics of all included patients. Clonality, diversity, and richness were calculated for each repertoire since these parameters robustly define the general repertoire architecture. Figures 1A–C summarizes the repertoire features of patients with chronic HCV infection and in patients with SVR after DAA treatment (and interferon treatment) as well as HDs. We found substantially increased B cell richness in HCV B cell repertoires independently of disease activity and viral clearance. Moreover, patients with HCV, regardless of their clinical status, had higher rates of somatically hypermutated B cells as compared to healthy controls (Figure 1D). This increase persisted in individuals with SVR (Figure 1D). In line with prior studies,^23^,^24^ patients with HCV had B cell subsets with longer heavy-chain CDR3s independent of clinical status (Figure 1E). This overrepresentation was especially characteristic of IGHV1-69–encoded BCRs known for their broad HCV reactivity.^23^

**TABLE 1:** Characteristics of cohorts

	Healthy individuals	Patients with HCV
No. individuals	12	52
No. samples	12	59
Sex		
Female	9	33
Male	3	19
Age, y, median (range)	44 (26–86)	55.5 (26–79)
Viremia (IU/mL)		22–50 Mio IU/mL
Genotype (%)		
GT1		43.8
GT2		9.4
GT3		37.5
GT4		3.1
GT5		3.1
GT6		3.1
Frequency of cryoglobulinemia		3.3%
Treatment (%)		
Ledipasvir/sofosbuvir		20.69
Sofosbuvir/velpatasvir		20.69
Glecaprevir/pibrentasvir		17.24
Velpatasvir/sofosbuvir		13.78
Peginterferonalfa/ribavirin		10.35
Elbasvir/grazoprevir		3.45
Ombitasvir/paritaprevir/ritonavir		3.45
Ombitasvir/paritaprevir/ritonavir/dasabuvir		3.45
Sofosbuvir/ribavirin		3.45
Sofosbuvir/velpatasvir/voxilaprevir		3.45

### B cells are biased toward the usage of IGHV1-69 and IGHV4-59 rearrangements

We next calculated the global usage of productive V(D)J rearrangements to probe for HCV-related BCR signatures. These immune signatures were visualized per individual using a principal component analysis (Figure 1F). Interestingly, despite our bulk sequencing approach in a cohort without obvious clinical autoimmunity or cryoglobulinemia, we were able to statistically show repertoire skewing toward a specific IGHV(D)J gene usage regardless of clinical status (Figure 1F). Compared to HDs, this skewing mainly derived from the increased frequency of BCRs with defined IGHV1-69 and IGHV4-59 rearrangements in patients with HCV, similar to previous findings in lymphomas and HCV-associated autoreactive B cell clones (Figure 1G).^11^,^24^ This finding suggested systematic expansion of B cells with potential autoreactive or even lymphoma-like properties in patients with HCV.

### Sequence connectivity analysis shows convergent recombination to stereotyped CDR3 sequences in HCV that persist beyond successful treatment

**FIGURE 2:**
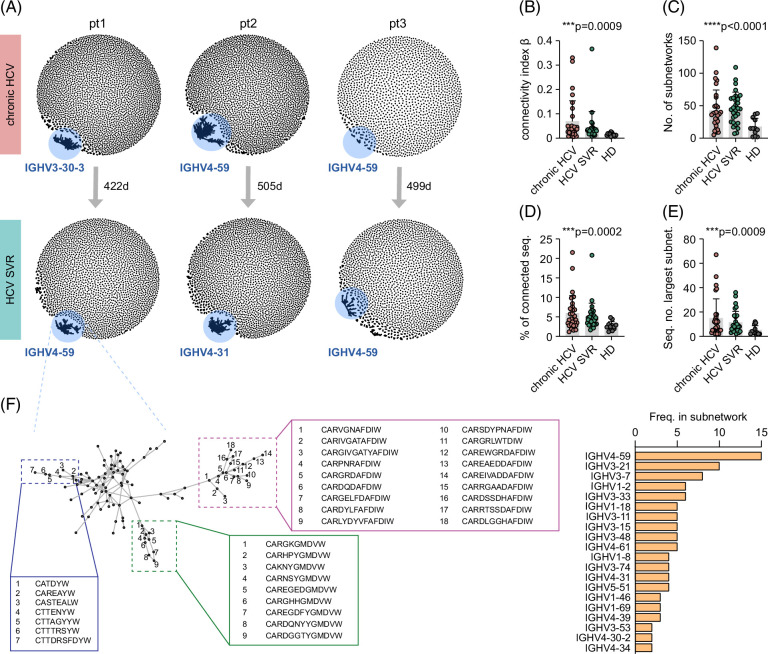
Connectivity analysis of B cells in HCV. (A) Petri dish plots showing B cell connectivity of 3 patients with HCV at active disease and with SVR, respectively. Each point/node corresponds to a clone. Clones are connected by a line/edge if their sequences differ with Levenshtein distance ≤3. (B) Quantitative B cell connectivity analysis for complete B cell repertoires, (C) number of B cell subnetworks, (D) percentage of connected B cell receptor sequences, and (E) size of largest B cell network in patients with chronic HCV and SVR compared to healthy individuals. Statistics: Welch ANOVA. (F) Exemplary subnetwork from patient 1 at SVR with selected CDR3 sequences highlighted. The dominant V-gene families and frequencies within the subnetwork are presented as a table. Abbreviations: CDR3, complementarity-determining region 3; SVR, sustained virological response.

Antibody sequence similarity relations are a fundamental architectural feature of B cell repertoires that can be captured quantitatively using network analysis.^25^ Since structural network parameters like connectivity or assortativity (degree of correlation between nodes) are robust factors to identify key patterns that describe the current state of an individual’s immune repertoire in health in disease,^25^ we next calculated B cell network connectivity to detect potential stereotypic CDR3 patterns characteristic of B cell non-Hodgkin lymphomas and autoreactive B cells. This was performed using a classical graph-based approach and is exemplarily visualized as petri dish plots for repertoires of patients 1, 2, and 3 (Figure 2A). Overall, quantitative analysis of CDR3 sequences showed substantially higher connectivity within the B cell repertoires of patients with HCV with chronic disease and SVR as compared to those of HDs (Figure 2B). We noticed more than a doubling in the number of subnetworks in chronic HCV and SVR compared to HDs (mean of 42 and 46 vs. 17; Figure 2C), a higher percentage of connected sequences (mean of 5.9 and 5.1 vs. 2.7%, Figure 2D), and larger networks in chronic HCV and SVR compared to HDs (mean largest subnetworks 14.7 and 11.4 vs. 5 nodes; Figure 2E).

When we calculated the biggest subnetwork for IGHV gene usage per individual, we found that in many cases, the most frequently used IGHV gene belonged to one of the gene families responsible for skewing. Yet, within individual subnetworks of similar CDR3 sequences, a variety of IGHV gene families was typically present (exemplarily shown in Figure 2F). This argued strongly in favor of B cell maturation trajectories characterized by convergent IGHV(D)J recombination toward specific stereotyped CDR3 sequences. Of note, high B cell connectivity persisted even years after HCV cure, suggesting substantial longevity of this repertoire imprint (Figure 2A).

### Specific IGHV1-69 point mutations defining HCV elite-neutralizing antibodies correspond to recurrent IGHV1-69 point mutations found in HCV-unrelated lymphomas

**FIGURE 3:**
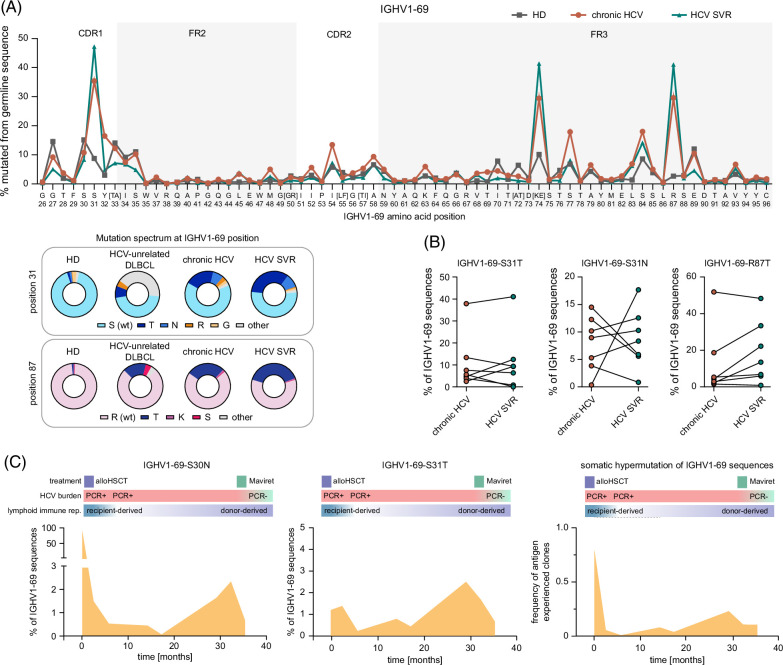
IGHV1-69 mutational hotspots in anti-HCV immunity and lymphoma antigen receptors. (A) Mutational frequencies within the IGHV1-69 gene in B cells from patients with chronic HCV, with SVR, and HDs. Mutational spectrum at hotspot position including data from IGHV1-69 expressing lymphomas^26^ as donut plots. (B) IGHV1-69-S31T, IGHV1-69-S31N, and IGHV1-69-R87T frequency pretreatment and posttreatment with DAA. The median time between DAA therapy and posttherapeutic sampling was 447 days. (C) Tracking of IGHV1-69 mutations in a patient with chronic HCV undergoing replacement of the adaptive immune system by hematopoietic stem cell transplantation for leukemia. Mutation frequencies are shown over time from the peritransplant period (recipient B lineage cells) to later stages in which donor-derived B cells are progressively exposed to HCV antigens. The last 2 time points reflect cessation of antigenic pressure after HCV cure. Abbreviations: DAA, direct-acting antiviral; HD, healthy donor; SVR, sustained virological response.

Specific point mutations within the variable regions of IGHV1-69–encoded antibodies with E2 specificity have been shown to define HCV elite neutralizers.^23^ Our data set contained a high mutation frequency at some of these CDR1 and framework region 3 hotspots within the IGHV1-69 gene both in patients with chronic HCV and SVR, even though these patients were not selected for HCV neutralization capacity (Figure 3A). Notably, the alignment of all 3593 IGHV1-69 sequences from our cohort to the 140 IGHV1-69–encoded antibody sequences with verified neutralizing capacity from Weber et al^23^ showed the prevalence of almost identical clonotypes in our repertoires (Supplemental Data S1, http://links.lww.com/HC9/A999). Of the previously described hotspot positions, especially positions IGHV1-69-S31 and IGHV1-69-R87 were heavily mutated in our cohort (>4 times more often than in HDs). We did, however, not detect an increased mutational frequency at position IGHV1-69-S30, which has also been reported for elite neutralizers.^23^ We aligned 2 of the most prominent HCV-specific hotspots from our data set—IGHV1-69-S31 and IGHV1-69-R87—with the clonotypic sequences from 24 IGHV1-69^+^ DLBCLs.^26^ Intriguingly, we noticed a similar mutational frequency and an overlapping spectrum of mutations at these positions in the HCV-derived and lymphoma-derived BCRs (Figure 3A). Given that these DLBCL cases were unrelated to HCV, we reasoned that these mutations—while mediating efficient HCV neutralization during an infection—may also have (predisposing) oncogenic potential beyond the context of HCV.

To substantiate this hypothesis, we next tested the general persistence of these mutations in the blood of patients with HCV. For this, we determined the mutational frequencies at the IGHV1-69-S31 and –R87 positions in samples acquired at the chronic HCV stage and after DAA treatment in all patients with matched blood samples. The median time between DAA treatment and blood sampling was 447 days. As shown in Figure 3B, in the majority of patients with HCV, the mutant clone persisted beyond HCV cure and did not trend toward decrement (Figure 3B). Given the persistence of B cell clones with mutated IGHV1-69 BCRs, we wished to track the kinetics of this BCR repertoire imprinting by HCV over time from early infection phases to later time points. Unfortunately, none of the patients in our cohort had preinfection blood samples available since all patients had been first diagnosed at the stage of chronic HCV infection. To approach this question, we longitudinally analyzed bone marrow B cells from a patient with chronic HCV infection who underwent an allogeneic stem cell transplant for myeloid leukemia. We found 2 classical HCV hotspot mutations in this patient (IGHV1-69-N30 and IGHV1-69-T31). When tracking these mutant sequences over time, we observed high mutational frequencies in the peritransplant period, reflecting residual recipient-derived B lineage cells (Figure 3C). This was followed by a dramatic decrease in mutational frequencies a few months after allogeneic stem cell transplant, reflecting the expansion of HCV-naïve donor-derived B lineage cells. Over 2 years, the mutational frequencies in HCV-specific hotspot regions of IGHV1-69 increased substantially (Figure 3C). After viral elimination by DAA therapy, these clones showed again a moderate drop but were not eliminated from the repertoire (Figure 3C).

### HCV B cells show lymphoma-like transcriptomes at single-cell resolution

The finding of overlapping mutational patterns in HCV-neutralizing antibodies/BCRs and HCV-unrelated lymphoma BCRs suggested that these mutations may confer oncogenic properties to the antigen receptor.

**FIGURE 4:**
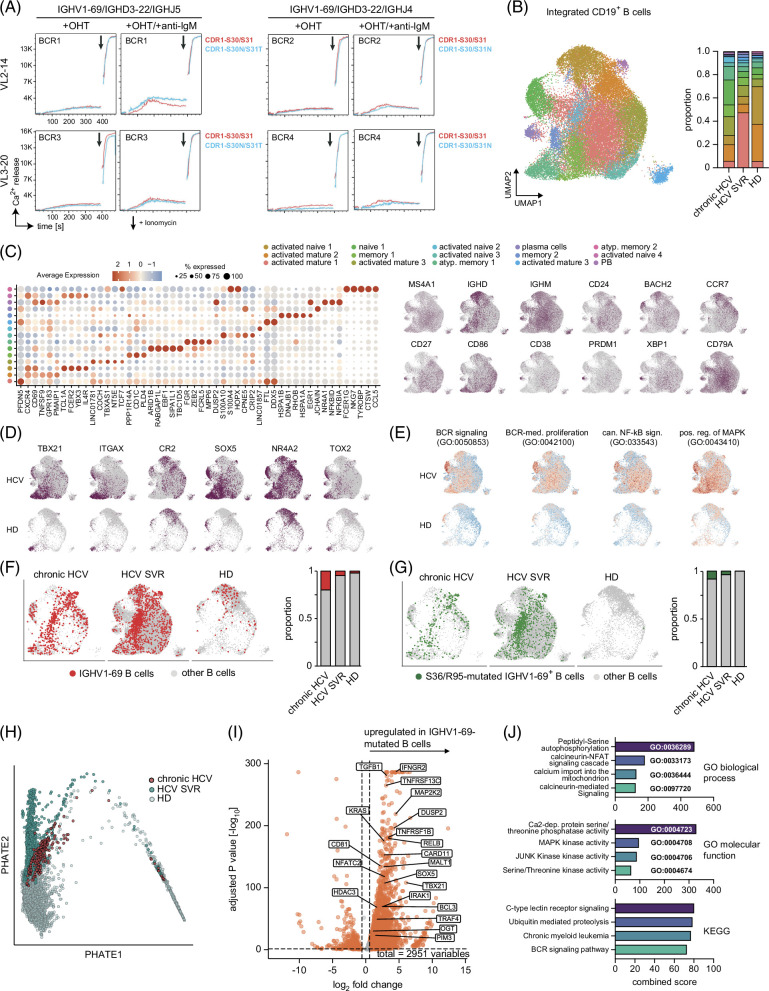
Lymphoma-like transcriptomes of B cells carrying IGHV1-69 BCR with HCV hotspot mutations. (A) Ca2+ influx analyses of TKO cells expressing lymphoma-derived IGHV1-69 BCRs with (IGHV1-69-N30/T31 or IGHV1-69-N31) or without HCV hotspot mutations upon stimulation with 4-OHT or 4-OHT plus anti-IgM antibody. Stimulations were started after recording 30 seconds of baseline signal. (B) UMAP projection of an integrated PBMC data set from 2 HDs, 2 patients with chronic HCV, and 5 patients with SVR (HD: 4585 cells; chronic HCV: 2777 cells, HCV SVR: 29,509 cells). Cluster proportions are shown as stacked bar plots. (C) The top 5 differentially expressed genes per cluster are shown as dot plots, and the expression of signature genes for cluster definition as violin plots. Expression of selected B cell signature genes as feature plots. (D) Feature plots showing T-bet positive atypical B cell populations in patients with HCV and HDs. (E) Feature plots showing enrichment for BCR-related pathways in patients with HCV and HDs. B cells expressing IGHV1-69 (F) and IGHV1-69-S36 or IGHV1-69-R87 mutations (G) were marked within the indicated groups. Stacked bar plots show the percentage of these cells within the B cell repertoire. (H) PHATE mapping shows overlapping trajectories between B cells with IGHV1-69 hotspot mutations that were derived from patients with chronic HCV, SVR, and healthy donor groups. (I) Differentially expressed genes in HCV B cells with IGHV1-69-S36 or IGHV1-69-R87 mutations. Cutoffs: adjusted *p* value <0.05 and log_2_ fold change </>0.58. (J) Upregulated genes were used as input for pathway analysis using enrichR. Top 4 hits for indicated pathway annotation sets. Abbreviations: BCR, B cell receptor; HD, healthy donor; PBMC, peripheral blood mononuclear cell; PHATE, Potential of Heat-diffusion for Affinity-based Transition Embedding; SVR, sustained virological response; TKO, triple knockout; UMAP, uniform manifold approximation and projection.

Since some of the BCRs used by DLBCL cells show autonomous signaling capacity, we wished to rule out that the hotspot mutations created similar antigen-independent signaling patterns. To this end, we cloned combinations of 2 DLBCL-derived IGHV1-69 rearrangements^26^ that carried HCV-typical hotspot mutations (IGHV1-69-N30/T31 or IGHV1-69-N31) along with 2 matching LC sequences for expression in TKO cells (BCR1-4, Supplemental Table S1, http://links.lww.com/HC9/A999), a murine TKO cell line that can be induced to express the BCR downstream signaling machinery upon 4-OHT challenge. In addition, the CDR1 sequences of these 4 BCRs were reverted to the germline configuration and also expressed in TKO cells (Supplemental Table S1, http://links.lww.com/HC9/A999). While all of the BCRs showed anti-IgM-induced Ca^2+^ influx, none of the tested BCRs showed autonomous signaling capacity, no matter if the original mutant CDR1 or the germline-reverted CDR1 were expressed (Figure 4A). We noticed, however, that BCR1, a receptor with 2 CDR1 hotspot mutations (IGHV1-69-N30/T31) and a VL2-14-encoded LC, had a slightly higher response to IgM ligation compared to the germline-reverted CDR1 (Figure 4A). Interestingly, pairing of the IGHV1-69 sequence from BCR1 with the VK3-20 LC (BCR3) did not result in CDR1-related differences in anti-IgM responsiveness (Figure 4A). Together, these data suggested that basic receptor functionality was preserved or even increased in IGHV1-69 variants carrying HCV hotspot mutations, but no autonomous signaling was induced.

Next, we asked whether B cells from patients with chronic HCV carrying such hotspot mutations showed specific transcriptome signatures. To look at this at single-cell resolution, we subjected sorted HCV and HD B cells to combined single-cell RNA and BCR V(D)J sequencing. We compared B cells from 2 HDs with those from 2 patients with chronic HCV and 5 with SVR. The 5 patients with HCV with SVR had received their DAA treatment at a median of 403 days (range: 360–422 d) before blood sampling. Figure 4B shows the uniform manifold approximation and projection projection of the integrated PBMC data set (HD: 4584 cells; chronic HCV: 2777 cells, HCV SVR: 29,509 cells). Clusters were shared by all disease groups and were defined based on the expression of classic B cell surface markers for naïve/mature, memory, and plasma/plasmablast stages (Figures 4B–D; Supplemental Figures S1A, B, http://links.lww.com/HC9/A999). The distribution of B cell subpopulations differed between groups with HCV SVR patients exhibiting an intermediate distribution between chronic HCV and HDs (Figures 4B, C). While HDs had a higher proportion of naïve/mature B cells with signatures of activation (*CD69*, *GPR183*, *NT5E*, *EBF1*, and *CXCR4*), patients with HCV showed more antigen-experienced class-switched memory-like B cell subsets (*CD27*, *CD1C*, and *PPP1R14A*) in line with the strong somatic hypermutation observed by repertoire deep sequencing (Figures 4B, C; Supplemental Figure S1B, http://links.lww.com/HC9/A999). Patients with HCV SVR were enriched for 2 clusters with activated mature-like transcription patterns (*CXCR4* and *GPR183*) that were characterized by high expression of *PFDN5*, *FTL*, and *DDX5* and a memory-like cluster with high expression of *EGR1*, *HSPA1A*, *HSPA1B*, and *DNAJB1* (Figure 4B). Notably, a highly similar cluster (activated mature 3) was expanded in patients with chronic HCV (Figure 4B). HCV samples also had a higher proportion of memory B cells (Figure 4B). In addition, we observed the HCV-specific expansion of a cluster that was characterized by the expression of T-bet (*TBX21*) and further positive and negative key markers of atypical B cells^27^,^28^ as well as by *ZEB2, FGR*, and *FCRL5* expression (Figures 4B–D). These clusters also displayed a pronounced signature of BCR-mediated signaling, proliferation, and NF-κB and MAPK activity in HCV (Figure 4E).

Patients with chronic HCV and SVR showed high numbers of IGHV1-69 expressing B cells making up to 20% of the whole repertoire (Figure 4F), many with mutations characteristic of HCV-neutralizing antibodies (Figure 4G). The percentage of those cells in the repertoire was higher in patients with chronic HCV as compared to SVR (Figures 4F, G). Notably, 35.5% of all HCV-associated IGHV1-69 sequences, which corresponds to 5.9% of all detected B cells, exhibited high homology to IGHV1-69–encoded BCRs with validated neutralizing capacity^23^ (Supplemental Data S2, http://links.lww.com/HC9/A999). To further characterize this expanded population, we performed PHATE analysis on HCV patient-derived B cells carrying IGHV1-69 with HCV-typical hotspot mutations. This analysis showed a clear separation of this class of B cells from HD B cells, while most cells from patients with chronic HCV overlapped with cells from SVR (Figure 4H). Differentially regulated genes were enriched for classical lymphoma driver genes downstream of the BCR, including *MALT1* and *CARD11*, as well as *NFATC2*, *TNFRSF13C* (BAFF), and components of the NF-κB (*RELB*, *IRAK1*, *BCL3*, and *TNFRSF1B*) pathway (Figure 4I). Broader pathway analysis confirmed these data and showed that also other parts of this signaling cascade were deregulated, such as the calcineurin-NFAT axis as well as other pathways known to synergize with BCR signaling, such as Ca2+-dependent protein phosphatase activity as well as MAPK and JUNK kinase activity (Figure 4J).

## DISCUSSION

Lymphomas are characterized by the uncontrolled growth of clonal lymphocytes. Risk factors include age, family history, autoimmune disease, immunosuppression, exposure to chemicals or radiation, genetic risk factors, and specific bacterial or viral infections. While some viruses, such as EBV, HHV-8, and HTLV-1, can directly transform lymphocytes, the B cell dyscrasias and B cell lymphomas arising in patients chronically infected with HCV have long been suspected to be driven by chronic immune stimulation, either indirectly by a chronic inflammatory environment such as in Helicobacter-driven lymphomas or more directly through chronic stimulation of the BCR pathway by HCV antigens.^3^,^11^,^12^,^13^ Yet, the exact pathophysiological phenomena underlying the association of HCV and lymphomas remained unclear.

In our work, we characterized the peripheral blood B cell architecture of patients with chronic HCV that—clinically—did not suffer from B cell dyscrasias such as cryoglobulinemia or lymphoma. We found a characteristic cross-individual HCV fingerprint essentially consisting of lymphoma-like immunoglobulin gene usage, high somatic hypermutation, and stereotypic CDR3 motifs. Unexpectedly, this fingerprint persisted in the majority of patients even years after successful HCV therapy. Moreover, we pinpointed mutations of serine 31 within the CDR1 of the IGHV1-69 gene that had recently been identified to facilitate broad neutralization of antibodies against HCV E2.^23^ While serine 31 mutations have been reported in small cohorts of HCV-associated lymphomas (3/8 patients with S31T and 1/8 patients with S31N in Ivanovski et al^29^; 1/3 with S31T and 1/3 with S31N in Marasca et al^30^), we show that this residue is also frequently mutated in HCV-unrelated high-grade lymphomas.

It has been widely recognized over the past decade that the antigen receptor, with its specific configuration, is a powerful tumor promotor in lymphoma. Major revelations were the discovery of preferentially used immunoglobulin genes (eg, IGHV4-34, IGHV4-59, or IGHV1-69) to the extent of virtually identical “stereotyped” receptor conurations,^13^,^31^,^32^ their reactivity with self-antigens,^31^,^33^,^34^,^35^ their autonomous signaling capacity through BCR-internal epitopes^34^,^36^ as well as the clinical success of new therapies interfering with antigen receptor signaling. Through these studies, it became clear that lymphoma BCRs harbor recurrent sequence motifs that are rare or absent in healthy lymphocytes, for example, the chronic lymphocytic leukemia-specific LC point mutation IGLV3-21^R110^.^37^,^38^,^39^ The acquisition of enhancing IGHV1-69 HCV-neutralizing hotspot mutations during infection that are at the same time recurrent aberrations in BCRs of HCV-unrelated DLBCLs does add a whole new perspective on the potential risks underlying antiviral immune responses. Our data suggest that in the case of HCV, broad virus neutralization may come at the cost of generating BCR variants with increased oncogenic potential that mimic the chronic active BCR signaling phenotype that is especially characteristic for chronic lymphocytic leukemia, ABC DLBCL, and marginal zone lymphoma, while other lymphoid malignancies like Burkitt’s lymphoma depend on tonic BCR signals.^40^ Notably, DLBCLs and marginal zone lymphomas represent the B cell non-Hodgkin lymphoma classes most commonly associated with HCV infection.^41^ Chronic active BCR signaling is typified by antigen-independent activation of NF-κB, MAPK, and NFAT signaling, while tonic BCR signaling exclusively engages the PI3K pathway.^40^ In line with this, we observed that HCV-derived B cells expressing IGHV1-69–encoded BCRs with CDR1 hotspot mutations upregulated, besides classical lymphoma drivers downstream of the BCR like MYC, NF-κB, MAPK, and NFAT pathway components such as *CARD11*, *MALT1*, *RELB*, *PIM3*, *KRAS*, *MAP2K2*, *BCL3*, and *NFATC2*. As a consequence, the persistence of expanded B cell networks may not simply be regarded as an uncleared and inert BCR-centered “HCV scar,” but as the result of imprinted lymphoma-like BCR signaling maintaining the persistence of these clonal networks. Notably, we did not observe a clear enrichment of these B cells in a distinct cellular subset, possibly due to the limited sample size of our cohort. It will be important to assess in future studies whether clonotypes with the IGHV1-69-mutated BCRs are part of a distinct B cell population or show phenotypic and functional plasticity depending on disease state and time point relative to HCV infection.

One mechanistic explanation of how upstream IGHV1-69 hotspot mutations can imprint persisting lymphoma-like signaling comes from the superior responses to IgM ligation for one of the lymphoma-derived BCRs with mutated CDR1 (BCR1). It is plausible that specific mutation-induced steric changes in receptors of this family, with their inherent propensity for autoreactivity, lower the activation threshold for cross-reaction with self-antigens. This is in line with recent insights into BCR structure that revealed that the amino acid–dependent conformation of extracellular and membrane-associated BCR domains can have an even more prominent impact on activation thresholds and signaling strengths of individual BCRs than on their affinity for the respective antigen,^42^,^43^,^44^ and also illustrated by the LC dependency of the here observed activation effect. A prominent example in this regard is the IGLV3-21^R110^ LC mutation that, in concert with several conserved amino acids in the HC, confers autonomous signaling by mediating homotypic interactions in up to 15% of patients with chronic lymphocytic leukemia.^37^,^38^,^45^ It is important to note that BCR1 not only carries 2 CDR1 hotspot mutations but also shares other characteristics with classical elite-neutralizing HCV antibodies, for example, the long and hydrophobic CDR3, the hydrophobic CDR2, and the rather high mutation rate of 94% identity to germline.^23^ The requirement for multiple BCR alterations in combination with proper LC usage might contribute to the only moderately elevated lymphoma risk of patients with HCV.

While highly or broadly neutralizing IGHV1-69–encoded antibodies specific for the E2 envelope protein are an important contributor to spontaneous viral clearance or long-term disease control,^46^,^47^ immune responses targeting other viral proteins can achieve similar outcomes. Of particular interest are antibody responses against the nonstructural protein NS5 that have been associated with spontaneous viral clearance.^48^ Interestingly, B cells from these patients with HCV showed higher clonal evolution and substantially higher proliferative capacity in vitro as B cells from patients with chronic HCV and NS5 antibody response.^48^ Further studies will be necessary to assess whether NS5-directed B cell responses result in similar BCR imprinting as described here.

From a clinical perspective, the substantial longevity of the HCV-induced oncogenic imprints despite cessation of antigenic pressure may provide an explanation for the increased lymphoma risk even after HCV cure that has been suggested by some cohort studies. One of these studies showed that 3 of 431 DAA-treated patients were afterward diagnosed with high-grade non-Hodgkin lymphoma; the prevalence in this cohort was 696 per 100,000—about 30 times higher compared with the general population.^49^ Also, El-Serag et al^50^ demonstrated no impact of DAA treatment on non-Hodgkin lymphoma risk.

Importantly, our sequence-based approach is not definitive proof of the HCV-neutralizing capacity of the mutated BCRs and may be interpreted as a study limitation. Nevertheless, the proposed model of HCV-induced immune imprinting does not necessarily mandate that all IGHV1-69 BCRs exhibit a high neutralizing capacity. Rather, we suggest that the multistep affinity maturation process triggered by an anti-HCV immune response may generate increased numbers of IGHV1-69–encoded BCRs with the described point mutations.

Together, these data contribute a new perspective on how antiviral immune responses may foster lymphomagenesis and should raise our awareness of elevated lymphoma risk in successfully treated patients with HCV in the DAA era.

## Supplementary Material

SUPPLEMENTARY MATERIAL
