# Supplementary material for: B cells expressing mutated IGHV1-69–encoded antigen receptors related to virus neutralization show lymphoma-like transcriptomes in patients with chronic HCV infection
Source: Hepatol Commun. 2024 Jul 31;8(8):e0503. doi: 10.1097/HC9.0000000000000503 (PMC12333805; doi:10.1097/HC9.0000000000000503)
Supplement: SUPPLEMENTARY MATERIAL [file hc9-8-e0503-s001.docx]

**B cells expressing mutated IGHV1-69-encoded antigen receptors related to virus-neutralization show lymphoma-like transcriptomes in patients with chronic HCV infection**

Christoph Schultheiß, Edith Willscher, Lisa Paschold, Christin Ackermann, Moritz Escher, Rebekka Scholz, Maximilian Knapp, Jana Lützkendorf, Lutz P. Müller, Julian Schulze zur Wiesch, and Mascha Binder

Table of contents

Supplementary Table S1…………………………………………………………………..2

Supplementary Figure S1………………………………………………………………….3

Supplementary Data S1……………………………………………………………………4

Supplementary Data S2…………………………………………………………………..17

**Supplementary Table S1. CDR3 Sequences of cloned BCRs.**

|  | HC_V | HC_D | HC_J | HC_CDR1 | HC_CDR3 | LC |
| --- | --- | --- | --- | --- | --- | --- |
| BCR1_CDR1^N30/T31^ | IGHV1-69*01 | IGHD3-22*01 | IGHJ5*02 | GDPF**NT**YG | CARDSDNFDSSGYSYNWFDPW | IGLV2-14*02 |
| BCR1_CDR1^S30/S31^ | IGHV1-69*01 | IGHD3-22*01 | IGHJ5*02 | GGTF**SS**YA | CARDSDNFDSSGYSYNWFDPW | IGLV2-14*02 |
| BCR2_CDR1^S30/N31^ | IGHV1-69*06 | IGHD3-22*01 | IGHJ4*02 | GGTF**SN**YA | CARGPDSSSYYYFYW | IGLV2-14*02 |
| BCR2_CDR1^S30/S31^ | IGHV1-69*06 | IGHD3-22*01 | IGHJ4*02 | GGTF**SS**YA | CARGPDSSSYYYFYW | IGLV2-14*02 |
| BCR3_CDR1^N30/T31^ | IGHV1-69*01 | IGHD3-22*01 | IGHJ5*02 | GDPF**NT**YG | CARDSDNFDSSGYSYNWFDPW | IGKV3-20*01 |
| BCR3_CDR1^S30/S31^ | IGHV1-69*01 | IGHD3-22*01 | IGHJ5*02 | GGTF**SS**YA | CARDSDNFDSSGYSYNWFDPW | IGKV3-20*01 |
| BCR4_CDR1^S30/N31^ | IGHV1-69*06 | IGHD3-22*01 | IGHJ4*02 | GGTF**SN**YA | CARGPDSSSYYYFYW | IGKV3-20*01 |
| BCR4_CDR1^S30/S31^ | IGHV1-69*06 | IGHD3-22*01 | IGHJ4*02 | GGTF**SS**YA | CARGPDSSSYYYFYW | IGKV3-20*01 |

**
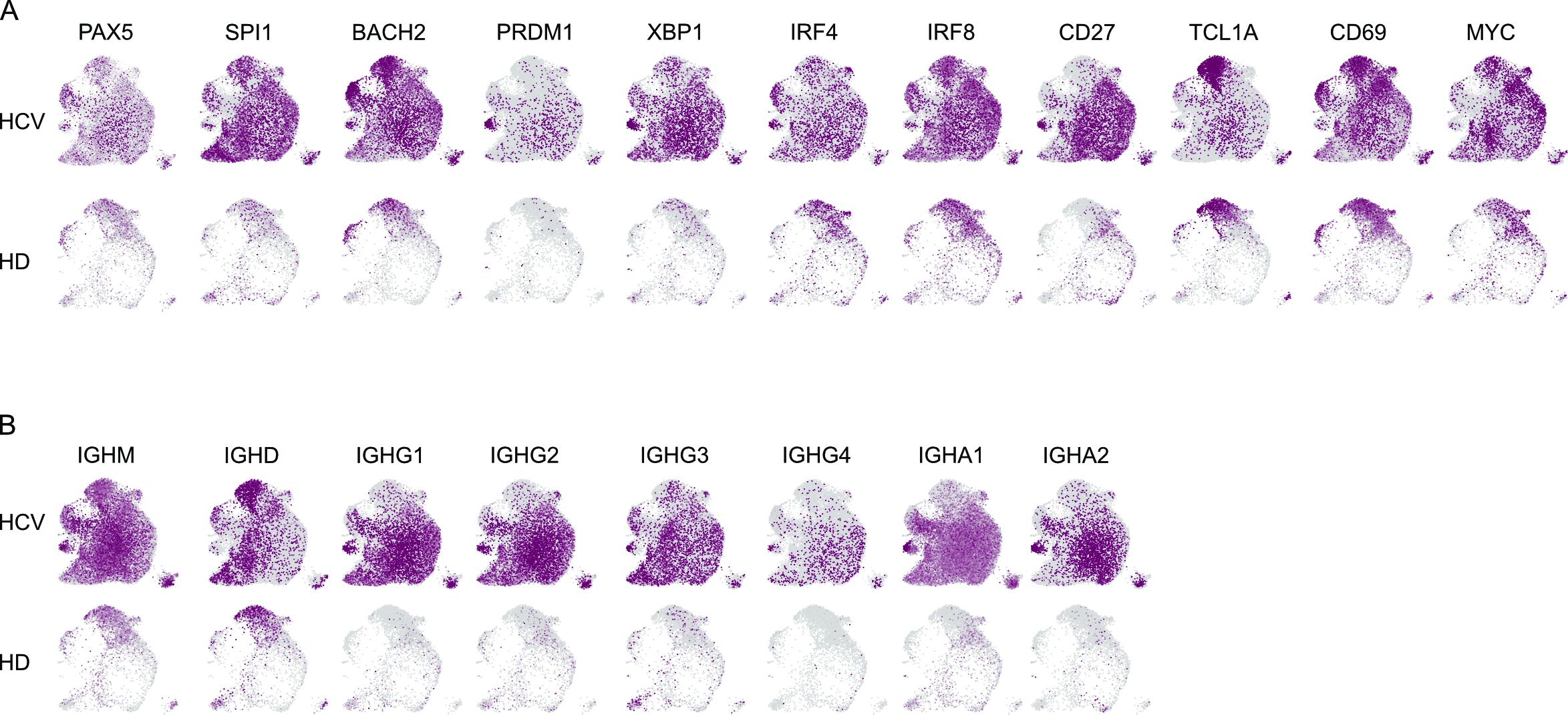
**

**Supplementary Figure S1. Gene expression of B cell markers.** UMAP projection of an integrated PBMC dataset from two healthy donors (HD), two patients with chronic HCV and five patients with sustained virological response (SVR) (HD: 4585 cells; chronic HCV: 2777 cells, HCV SVR: 29509 cells). (A) Key markers for B cell lineage commitment and differentiation. (B) Expression of immunoglobulin constant regions.

**Supplementary Data S1:**

Alignment of 3593 IGHV1-69 sequences from our bulk NGS cohort to 144 IGHV1-69 sequences of validated HCV-neutralizing antibodies published by Weber et al [1]. All sequences were first gapped according to their germline alignment, then levenshtein distance was calculated for each pair of sequences.

**A. Alignment with up to two exchanges_________________________________________**

Weber et. al Sequences 1198_01_E11 and 1198_03_G07

GGTF....SSYAISWVRQAPGQGLEWMGGIIPI..FGTANYAQKLQ.DRVTITADKSTSTAYMELSSLRSEDTAVYYC

Aligned Sequences from our cohort

S.217 GGTF....SSYAISWVRQAPGQGLEWMGGIIPI..FGTANYAQKFQ.DRVTITADKSTSTAYMELSSLRSEDTAVYYC

S.324 GGTF....SSYAISWVRQAPGQGLEWMGGIIPI..FGTANYAQKFQ.DRVTITADKSTSTAYMELSSLTSEDTAVYYC

S.2797 GGTF....SSYAISWVRQAPGQGLEWMGGIIPI..FGTGNYAQKFQ.DRVTITADKSTSTAYMELSSLRSEDTAVYYC

And 186 Sequences with the following motif

GGTF....SSYAISWVRQAPGQGLEWMGGIIPI..FGTANYAQKFQ.GRVTITADKSTSTAYMELSSLRSEDTAVYYC

**B. Alignment with up to three exchanges_______________________________________**

Weber et. al Sequences 1198_01_E11 and 1198_03_G07

GGTF....SSYAISWVRQAPGQGLEWMGGIIPI..FGTANYAQKLQ.DRVTITADKSTSTAYMELSSLRSEDTAVYYC

Aligned Sequences (n=510) from our cohort

B.197 B.198 B.227 B.301 B.307 B.327 B.363 B.2603 B.2776 B.2848

GGTF....STYAISWVRQAPGQGLEWMGGIIPI..FGTANYAQKFQ.GRVTITADKSTSTAYMELSSLRSEDTAVYYC

B.200 B.220 B.2842

GGTF....SSYAISWVRQAPGQGLEWMGGIIPI..FGTANYAQKFQ.GRVTITADKSTSTAYMELNSLRSEDTAVYYC

B.205 GGTF....SSYAISWVRQAPGQGLEWMGGIIPI..FGTANYAQKFQ.GRVTITADKSTSTSYMELSSLRSEDTAVYYC

B.206 GGTF....SSYAISWVRQVPGQGLEWMGGIIPI..FGTANYAQKFQ.GRVTITADKSTSTAYMELSSLRSEDTAVYYC

B.241 B.1563

GGTF....SSYAITWVRQAPGQGLEWMGGIIPI..FGTANYAQKFQ.GRVTITADKSTSTAYMELSSLRSEDTAVYYC

B.268 B.1332

GGTF....SSYAISWVRQAPGQGLEWMGGIIPI..FGTANYAQKFQ.GRVTITADKSTSTAYMELSSLRSDDTAVYYC

B.279 B.308

GGTF....SSYAISWVRQAPGQGLEWMGGIIPI..FGTANYAQKFQ.GRVTITADTSTSTAYMELSSLRSEDTAVYYC

B.294 GGTF....SSYAISWVRQAPGQGLEWMGGIIPI..FGTANYAQKFQ.GRVTITADKSTSTAYMDLSSLRSEDTAVYYC

B.323 GGTF....SSYAISWVRQAPGQGLEWMGGIIPI..FGTANYAQKFQ.DRVTITADKSTSTAYMELSGLTSEDTAVYYC

B.334 GGTF....SSYAISWVRQAPGQGLEWMGGIIPI..FGTANYAQKFQ.GRVTITADKSTSTAYMEMSSLRSEDTAVYYC

B.362 GGTF....SSYAISWVRQAPGQGLEWMGGIIPI..FGTANYAQKFQ.GRVTITADKSTSTAYMELSSLRSEDAAVYYC

B.467 GGTF....SSYAISWVRQAPGQGLEWMGGIIPI..FGTANYAQKFQ.GRVTITADKSTSTAYMELRSLRSEDTAVYYC

B.485 GGTF....SSYAISWVRQAPGQGLEWMGGIIPI..FGTANYAQKFQ.GRVTITADKSTSTAYMEVSSLRSEDTAVYYC

B.696 GGTF....SSYAISWVRQAPGQGLEWMGGIIPI..FGTANYAQKFQ.GRVTITADKSTSTAYMELSNLRSEDTAVYYC

B.930 B.2820

GGTF....SSYAISWVRQAPGQGLEWMGGIFPI..FGTANYAQKFQ.GRVTITADKSTSTAYMELSSLRSEDTAVYYC

B.1019 GGTF....SSYAVSWVRQAPGQGLEWMGGIIPI..FGTANYAQKFQ.GRVTITADKSTSTAYMELSSLRSEDTAVYYC

B.1051 GGTF....SSYAISWVRQAPGQGLEWMGGIIPI..FGTANYAQKFQ.GRVTITADKSTSTAYMELSSLRSEDTTVYYC

B.1156 GGTF....SSYAISWVRQAPGQGLEWMGGIIPI..FITANYAQKFQ.GRVTITADKSTSTAYMELSSLRSEDTAVYYC

B.1175 GGTF....SSYAISWVRQAPGQGLEWMGGIIPI..FGTANYAQKFQ.GRVTITADKSTSTAYMELSSLRSEYTAVYYC

B.1259 B.1602 B.2631 B.3470 B.3481 B.3482 B.3484 B.3490 B.3496

GGTF....SSYAISWVRQAPGQGLEWMGGIIPI..FGIANYAQKFQ.GRVTITADKSTSTAYMELSSLRSEDTAVYYC

B.1270 GDTF....SSYAISWVRQAPGQGLEWMGGIIPI..FGTANYAQKFQ.GRVTITADKSTSTAYMELSSLRSEDTAVYYC

B.1327 GCTF....SSYAISWVRQAPGQGLEWMGGIIPI..FGTANYAQKFQ.GRVTITADKSTSTAYMELSSLRSEDTAVYYC

B.1345 GGTF....SSYAISWVRQAPGQGLEWMGGIIPI..FGTANYAQKFQ.GRVTITADKSTSTVYMELSSLRSEDTAVYYC

B.1565 GGTF....SSYAISWVRQAPGQGLEWMGGIIPI..FGTANYGQKFQ.GRVTITADKSTSTAYMELSSLRSEDTAVYYC

B.1590 GGTF....SSYAISWVRQAPGQGLEWMGGIIPI..FGTAKYAQKFQ.GRVTITADKSTSTAYMELSSLRSEDTAVYYC

B.1591 GGTL....SSYAISWVRQAPGQGLEWMGGIIPI..FGTANYAQKFQ.GRVTITADKSTSTAYMELSSLRSEDTAVYYC

B.1599 GGTF....SSYAISWVRQAPGQGLEWMGGIIPI..FGTANYPQKFQ.GRVTITADKSTSTAYMELSSLRSEDTAVYYC

B.1905 GGTF....SSYAISWVRQAPGQGLEWMGGIIPI..FGTANYAQKFQ.GRVTITADKSTSTAYMELSSLRPEDTAVYYC

B.2288 GGTF....SSYAISWLRQAPGQGLEWMGGIIPI..FGTANYAQKFQ.GRVTITADKSTSTAYMELSSLRSEDTAVYYC

B.2309 GGTF....SSYAISWVRQAPGQGLEWMGGIIPI..FGTANYAQRFQ.GRVTITADKSTSTAYMELSSLRSEDTAVYYC

B.2757 B.2809 B.2826 B.2951

GGTF....SNYAISWVRQAPGQGLEWMGGIIPI..FGTANYAQKFQ.GRVTITADKSTSTAYMELSSLRSEDTAVYYC

B.2769 GGTF....STYAISWVRQAPGQGLEWMGGIIPI..FGTANYAQKFQ.DRVTITADKSTSTAYMELRSLRSEDTAVYYC

B.2773 GGTF....SSYAISWVRQAPGQGLEWMGGIIPI..FATANYAQKFQ.GRVTITADKSTSTAYMELSSLRSEDTAVYYC

B.2780 GGTF....TSYAISWVRQAPGQGLEWMGGIIPI..FGTANYAQKFQ.GRVTITADKSTSTAYMELSSLRSEDTAVYYC

B.2784 GGTF....SSYAISWVRQAPGQGLEWMGGIIPI..FGTANYAQNFQ.DRVTITADKSTSTAYMELSSLRSEDTAIYYC

B.2795 GGTF....SSYAISWVRQAPGQGLEWMGGIIPI..FGTANYAQKFQ.GRVTITADKSTSTAYMGLSSLRSEDTAVYYC

B.2799 GGTF....TSYAISWVRQAPGQGLEWMGGIIPI..FGTANYAQNFQ.DRVTITADKSTSTAYMELSSLRSEDTAVYYC

B.2857 GGTF....SSYAISWVRQAPGQGLEWMGGIIPI..FGTANYAQKFQ.GRVTITADKSTSTAYMELSSLRSEDTAIYYC

B.3077 GGTF....SSYAISWVRQAPGQGLEWMGGIIPI..FGAANYAQKFQ.GRVTITADKSTSTAYMELSSLRSEDTAVYYC

B.3161 GGTS....SSYAISWVRQAPGQGLEWMGGIIPI..FGTANYAQKFQ.GRVTITADKSTSTAYMELSSLRSEDTAVYYC

B.3202 GGTF....SSYAISWVRQAPGQGLEWMGGIIPI..FGTANYAQKFQ.GRVTITADKSTSTAYMELSSLRSEDTAVYCC

B.3249 GGTF....SSYAISWVRQAPGQGLEWMGWIIPI..FGTANYAQKFQ.GRVTITADKSTSTAYMELSSLRSEDTAVYYC

B.3256 GGTF....SSYAISWVRQAPGQGLEWMGGIIPI..FGTANYAQKFQ.GRVTITADKSTSTAYVELSSLRSEDTAVYYC

B.3578 GGTF....SSYGISWVRQAPGQGLEWMGGIIPI..FGTANYAQKFQ.GRVTITADKSTSTAYMELSSLRSEDTAVYYC

B.3591 GGTF....SSYAISWVRQAPGQGLEWMGGIIPI..FGTANYAQKFQ.GRVTITADKFTSTAYMELSSLRSEDTAVYYC

B.3608 GGTF....SSYAISWVRQAPGQGLEWMGGIIPI..FGTANYAQKFQ.GRVTITADKSTSTAYLELSSLRSEDTAVYYC

And 438 Sequences with the followig motif GGTF....SSYAISWVRQAPGQGLEWMGGIIPI..FGTANYAQKFQ.GRVTITADESTSTAYMELSSLRSEDTAVYYC

**C. Alignment with up to four exchanges________________________________________**

Weber et. al Sequences 1198_01_E11 and 1198_03_G07

GGTF....SSYAISWVRQAPGQGLEWMGGIIPI..FGTANYAQKLQ.DRVTITADKSTSTAYMELSSLRSEDTAVYYC

Aligned Sequences (n=201) from our cohort

B.102 GGTF....SSYAISWVRQAPGQGLEWMGGIIPI..LGIANYAQKFQ.GRVTITADKSTSTAYMELSSLRSEDTAVYYC

B.111 GGTF....SSYAISWVRQAPGQGLEWMGGIIPI..LGIANYAQKFQ.GRVTITADKSTSTAYMELSSLRSEDTAVYYC

B.112 GGTF....SSYAISWVRQAPGQGLEWMGGIIPI..LGIANYAQKFQ.GRVTITADKSTSTAYMELSSLRSEDTAVYYC

B.123 GGTF....SSYAISWVRQAPGQGLEWMGGIIPI..LGIANYAQKFQ.GRVTITADKSTSTAYMELSSLRSEDTAVYYC

B.144 GGTF....SSYAISWVRQAPGQGLEWMGGIIPI..LGIANYAQKFQ.GRVTITADKSTSTAYMELSSLRSEDTAVYYC

B.145 GGTF....SSYAISWVRQAPGQGLEWMGGIIPI..LGIANYAQKFQ.GRVTITADKSTSTAYMELSSLRSEDTAVYYC

B.148 GGTF....SSYAISWVRQAPGQGLEWMGGIIPI..LGIANYAQKFQ.GRVTITADKSTSTAYMELSSLRSEDTAVYYC

B.152 GGTF....SSYAISWVRQAPGQGLEWMGGIIPI..LGIANYAQKFQ.GRVTITADKSTSTAYMELSSLRSEDTAVYYC

B.154 GGTF....SSYAISWVRQAPGQGLEWMGGIIPI..LGIANYAQKFQ.GRVTITADKSTSTAYMELSSLRSEDTAVYYC

B.162 GGTF....SSYAISWVRQAPGQGLEWMGGIIPI..LGIANYAQKFQ.GRVTITADKSTSTAYMELSSLRSEDTAVYYC

B.165 GGTF....SSYAISWVRQAPGQGLEWMGGIIPI..LGIANYAQKFQ.GRVTITADKSTSTAYMELSSLRSEDTAVYYC

B.169 GGTF....SSYAISWVRQAPGQGLEWMGGIIPI..LGIANYAQKFQ.GRVTITADKSTSTAYMELSSLRSEDTAVYYC

B.179 GGTF....SSYAISWVRQAPGQGLEWMGGIIPI..LGIANYAQKFQ.GRVTITADKSTSTAYMELSSLRSEDTAVYYC

B.371 GGTF....SSYAISWVRQAPGQGLEWMGGIIPI..LGIANYAQKFQ.GRVTITADKSTSTAYMELSSLRSEDTAVYYC

B.373 GGTF....SSYAISWVRQAPGQGLEWMGGIIPI..LGIANYAQKFQ.GRVTITADKSTSTAYMELSSLRSEDTAVYYC

B.375 GGTF....SSYAISWVRQAPGQGLEWMGGIIPI..LGIANYAQKFQ.GRVTITADKSTSTAYMELSSLRSEDTAVYYC

B.382 GGTF....SSYAISWVRQAPGQGLEWMGGIIPI..LGIANYAQKFQ.GRVTITADKSTSTAYMELSSLRSEDTAVYYC

B.385 GGTF....SSYAISWVRQAPGQGLEWMGGIIPI..LGIANYAQKFQ.GRVTITADKSTSTAYMELSSLRSEDTAVYYC

B.386 GGTF....SSYAISWVRQAPGQGLEWMGGIIPI..LGIANYAQKFQ.GRVTITADKSTSTAYMELSSLRSEDTAVYYC

B.389 GGTF....SSYAISWVRQAPGQGLEWMGGIIPI..LGIANYAQKFQ.GRVTITADKSTSTAYMELSSLRSEDTAVYYC

B.390 GGTF....SSYAISWVRQAPGQGLEWMGGIIPI..LGIANYAQKFQ.GRVTITADKSTSTAYMELSSLRSEDTAVYYC

B.391 GGTF....SSYAISWVRQAPGQGLEWMGGIIPI..LGIANYAQKFQ.GRVTITADKSTSTAYMELSSLRSEDTAVYYC

B.395 GGTF....SSYAISWVRQAPGQGLEWMGGIIPI..LGIANYAQKFQ.GRVTITADKSTSTAYMELSSLRSEDTAVYYC

B.397 GGTF....SSYAISWVRQAPGQGLEWMGGIIPI..LGIANYAQKFQ.GRVTITADKSTSTAYMELSSLRSEDTAVYYC

B.399 GGTF....SSYAISWVRQAPGQGLEWMGGIIPI..LGIANYAQKFQ.GRVTITADKSTSTAYMELSSLRSEDTAVYYC

B.401 GGTF....SSYAISWVRQAPGQGLEWMGGIIPI..LGIANYAQKFQ.GRVTITADKSTSTAYMELSSLRSEDTAVYYC

B.402 GGTF....SSYAISWVRQAPGQGLEWMGGIIPI..LGIANYAQKFQ.GRVTITADKSTSTAYMELSSLRSEDTAVYYC

B.403 GGTF....SSYAISWVRQAPGQGLEWMGGIIPI..LGIANYAQKFQ.GRVTITADKSTSTAYMELSSLRSEDTAVYYC

B.413 GGTF....SSYAISWVRQAPGQGLEWMGGIIPI..LGIANYAQKFQ.GRVTITADKSTSTAYMELSSLRSEDTAVYYC

B.415 GGTF....SSYAISWVRQAPGQGLEWMGGIIPI..LGIANYAQKFQ.GRVTITADKSTSTAYMELSSLRSEDTAVYYC

B.416 GGTF....SSYAISWVRQAPGQGLEWMGGIIPI..LGIANYAQKFQ.GRVTITADKSTSTAYMELSSLRSEDTAVYYC

B.417 GGTF....SSYAISWVRQAPGQGLEWMGGIIPI..LGIANYAQKFQ.GRVTITADKSTSTAYMELSSLRSEDTAVYYC

B.2971 GGTF....SSYAISWVRQAPGQGLEWMGGIIPI..LGIANYAQKFQ.GRVTITADKSTSTAYMELSSLRSEDTAVYYC

B.3305 GGTF....SSYAISWVRQAPGQGLEWMGGIIPI..LGIANYAQKFQ.GRVTITADKSTSTAYMELSSLRSEDTAVYYC

B.3390 GGTF....SSYAISWVRQAPGQGLEWMGGIIPI..LGIANYAQKFQ.GRVTITADKSTSTAYMELSSLRSEDTAVYYC

B.190 GGTF....SSYAISWVRQAPGQGLEWMGGIIPI..FGTANYAQKFQ.GRVTITADTSTSTAYMELSSLTSEDTAVYYC

B.208 GGTF....SSYAISWVRQAPGQGLEWMGGIIPI..FGTANYAQKFQ.GRVTITADTSTSTAYMELSSLTSEDTAVYYC

B.343 GGTF....SSYAISWVRQAPGQGLEWMGGIIPI..FGTANYAQKFQ.GRVTITADTSTSTAYMELSSLTSEDTAVYYC

B.2753 GGTF....SSYAISWVRQAPGQGLEWMGGIIPI..FGTANYAQKFQ.GRVTITADTSTSTAYMELSSLTSEDTAVYYC

B.2762 GGTF....SSYAISWVRQAPGQGLEWMGGIIPI..FGTANYAQKFQ.GRVTITADTSTSTAYMELSSLTSEDTAVYYC

B.2802 GGTF....SSYAISWVRQAPGQGLEWMGGIIPI..FGTANYAQKFQ.GRVTITADTSTSTAYMELSSLTSEDTAVYYC

B.194 GGTF....SSYAISWVRQAPGQGPEWMGGIIPM..FGTANYAQKFQ.GRVTITADKSTSTAYMELSSLRSEDTAVYYC

B.207 GGTF....SSYAISWVRQAPGQGLEWMGGIIPI..FGTANYAQKFQ.GRVTITADKSTSTAYMELTSLTSEDTAVYYC

B.210 GGTF....SSYAISWVRQAPGQGLEWMGGIIPI..FGTANYAQKFQ.GRATITADTSTSTAYMELSSLRSEDTAVYYC

B.213 GGTF....SSYAISWVRQAPGQGLEWMGGIIPI..FGTANYAQKFQ.GRVTITADKSTTTAYMELNSLRSEDTAVYYC

B.216 GGTF....STYAISWVRQAPGQGLEWMGGIIPI..FGTANYAQKFQ.DRVTITADTSTSTAYMELSSLTSEDTAVYYC

B.2813 GGTF....STYAISWVRQAPGQGLEWMGGIIPI..FGTANYAQKFQ.DRVTITADTSTSTAYMELSSLTSEDTAVYYC

B.230 GGTF....SSYAISWVRQAPGQGLEWMGGIIPI..FGTANYAQKFQ.GRVTITADKSTSTAYMELNSLRFEDTAVYYC

B.248 GGTF....STYAISWVRQAPGQGLEWMGGIIPI..FGTANYAQKFQ.DRVTITADKSTSTAYMELSGLTSEDTAVYYC

B.249 GGTF....SVYAISWVRQAPGQGLEWMGGIIPI..FGTANYAQKFQ.GRVTITADKSTSIAYMELSSLRSEDTAVYYC

B.259 GGTF....STYAISWVRQAPGQGLEWMGGIIPI..FGTANYAQKFQ.GRVTITADKSTSTAYMELNSLRSEDTAVYYC

B.2798 GGTF....STYAISWVRQAPGQGLEWMGGIIPI..FGTANYAQKFQ.GRVTITADKSTSTAYMELNSLRSEDTAVYYC

B.265 GGTF....STYAISWVRQAPGQGLEWMGGIIPI..FGTANYAQKFQ.GRVTITADKSTSTAYMELSSLRSEDTAVYFC

B.274 GGTF....ANYAISWVRQAPGQGLEWMGGIIPI..FGTANYAQKFQ.GRVTITADKSTSTAYMELSSLRSEDTAVYYC

B.275 GGTF....STYAISWVRQAPGQGPEWMGGIIPI..FGTANYAQKFQ.GRVTITADKSTSTAYMELSSLRSEDTAVYYC

B.2791 GGTF....STYAISWVRQAPGQGPEWMGGIIPI..FGTANYAQKFQ.GRVTITADKSTSTAYMELSSLRSEDTAVYYC

B.289 GGTF....SSYAISWVRQAPGQGLEWMGGIIPI..FGTANYAQKFQ.GRVTITADKSTSTAYMELRSLRSDDTAVYYC

B.290 GGTF....STYAISWVRQAPGQGLEWMGGIIPI..FGTANYAQKFQ.GRVTITADKSTSTAYMELSSLRFEDTAVYYC

B.293 GGTF....STYAISWVRQAPGQGLEWMGGIIPI..FGTANYAQKFQ.GRVTITADTSTSTAYMELSSLRSEDTAVYYC

B.297 GGTF....SSYAISWVRQVPGQGLEWMGGIIPI..FGTANYAQKFQ.GRVTITADRSTSTAYMELSSLRSEDTAVYYC

B.305 GGTF....STHAISWVRQAPGQGLEWMGGIIPI..FGTANYAQKFQ.GRVTITADKSTSTAYMELSSLRSEDTAVYYC

B.314 GGTF....SSYAISWVRQAPGQGLEWMGGIIPI..FGTANYAQKFQ.GRVTITADKSTSTAYMELSSLRSEDAAIYYC

B.325 GGTF....SSYAISWVRQAPGQGLEWMGGIIPI..FGTANYAPKFQ.GRVTITADTSTSTAYMELSSLRSEDTAVYYC

B.329 GGTF....SSYAISWVRQAPGQGLEWMGGIIPI..FGTANYAQKFQ.GRVTITADESRSTAYMELSSLRSEDTAVYYC

B.347 GGTF....STYAISWVRQAPGQGLEWMGGIIPI..FGTANYAQKFQ.GRVTITADKSTSTAYMELSSLRSDDTAVYYC

B.477 GGTF....SSYATSWVRQAPGQGLEWMGGIIPI..FGTANYAQKFQ.GRVTITADESTSTAYMELSSLRSEDTAVYYC

B.3023 GGTF....SSYATSWVRQAPGQGLEWMGGIIPI..FGTANYAQKFQ.GRVTITADESTSTAYMELSSLRSEDTAVYYC

B.484 GGTF....SSYAISWVRQAPGQGLEWMGGIIPI..FGTASYAQKFQ.GRVTITADESTSTAYMELSSLRSEDTAVYYC

B.526 GGTF....SSYAISWVRQAPGQGLEWMGGIIPI..FGTANYAQEFQ.GRVTITADESTSTAYMELSSLRSEDTAVYYC

B.536 GGTF....SSYAISWVRQAPGQGLEWMGGIIPI..FGTANYAQKFQ.GRVTITADESTSTAHMELSSLRSEDTAVYYC

B.571 GGTF....SSYAISWVRQAPGQGLEWMGGIIPL..FGTANYAQKFQ.GRVTITADESTSTAYMELSSLRSEDTAVYYC

B.621 GGTF....SSYAIIWVRQAPGQGLEWMGGIIPI..FGTANYAQKFQ.GRVTITADESTSTAYMELSSLRSEDTAVYYC

B.3284 GGTF....SSYAIIWVRQAPGQGLEWMGGIIPI..FGTANYAQKFQ.GRVTITADESTSTAYMELSSLRSEDTAVYYC

B.622 GGAF....SSYAISWVRQAPGQGLEWMGGIIPI..FGTANYAQKFQ.GRVTITADESTSTAYMELSSLRSEDTAVYYC

B.643 GGIF....NSYAISWVRQAPGQGLEWMGGIIPI..FGTANYAQKFQ.GRVTITADKSTSTAYMELSSLRSEDTAVYYC

B.676 GGIF....NSYAISWVRQAPGQGLEWMGGIIPI..FGTANYAQKFQ.GRVTITADKSTSTAYMELSSLRSEDTAVYYC

B.648 GGTF....CSYAISWVRQAPGQGLEWMGGIIPI..FGTANYAQKFQ.GRVTITADESTSTAYMELSSLRSEDTAVYYC

B.2088 GGTF....CSYAISWVRQAPGQGLEWMGGIIPI..FGTANYAQKFQ.GRVTITADESTSTAYMELSSLRSEDTAVYYC

B.708 GGTF....SSYTISWVRQAPGQGLEWMGGIIPI..FGTANYAQKFQ.GRVTITADESTSTAYMELSSLRSEDTAVYYC

B.712 GGTF....SSYTISWVRQAPGQGLEWMGGIIPI..FGTANYAQKFQ.GRVTITADESTSTAYMELSSLRSEDTAVYYC

B.908 GGTF....SSYTISWVRQAPGQGLEWMGGIIPI..FGTANYAQKFQ.GRVTITADESTSTAYMELSSLRSEDTAVYYC

B.1092 GGTF....SSYTISWVRQAPGQGLEWMGGIIPI..FGTANYAQKFQ.GRVTITADESTSTAYMELSSLRSEDTAVYYC

B.1240 GGTF....SSYTISWVRQAPGQGLEWMGGIIPI..FGTANYAQKFQ.GRVTITADESTSTAYMELSSLRSEDTAVYYC

B.1789 GGTF....SSYTISWVRQAPGQGLEWMGGIIPI..FGTANYAQKFQ.GRVTITADESTSTAYMELSSLRSEDTAVYYC

B.3155 GGTF....SSYTISWVRQAPGQGLEWMGGIIPI..FGTANYAQKFQ.GRVTITADESTSTAYMELSSLRSEDTAVYYC

B.742 GGTF....SSYAFSWVRQAPGQGLEWMGEIIPI..FGTANYAQKFQ.GRVTITADKSTSTAYMELSSLRSEDTAVYYC

B.749 GGTF....SSYAISWVRQAPGQGLEWMGGIIPI..FGRANYAQKFQ.GRVTITADESTSTAYMELSSLRSEDTAVYYC

B.856 GGTF....SSYAISWVRQAPGQGLEWMGGIIPI..FGTENYAQKFQ.GRVTITADESTSTAYMELSSLRSEDTAVYYC

B.861 GGTF....SSYAISWVRQAPGQGLEWMGGIIPI..FGTANYAQKFQ.GRVTITADESTSTAYMELSSLRSEDTAVHYC

B.876 GGTF....SSHAISWVRQAPGQGLEWMGGIIPI..FGTANYAQKFQ.GRVTITADESTSTAYMELSSLRSEDTAVYYC

B.897 GGTF....SSYAISWVRQAPGQGLEWMGGIIPI..FGTANYAQKFQ.GRVTINADESTSTAYMELSSLRSEDTAVYYC

B.899 GGTF....SSYAISWVRQAPGHGLEWMGGIIPI..FGTANYAQKFQ.GRVTITADKYTSTAYMELSSLRSEDTAVYYC

B.922 GGTF....SSYAISWVRQAPGQGLEWMGGIIPI..FGTANYAQRFQ.GRVTITADESTSTAYMELSSLRSEDTAVYYC

B.937 GGTF....SSYAISWVRQAPGQGLEWMGGIIPI..FGTANYAQKFQ.GRVTITADGSTRTAYMELSSLRSEDTAVYYC

B.970 GGTF....SSYAISWVRQAPGQGLEWMGGIIPI..FGTANYAQKFQ.GRVTITADESTRTAYMELSSLRSEDTAVYYC

B.1509 GGTF....SSYAISWVRQAPGQGLEWMGGIIPI..FGTANYAQKFQ.GRVTITADESTRTAYMELSSLRSEDTAVYYC

B.1651 GGTF....SSYAISWVRQAPGQGLEWMGGIIPI..FGTANYAQKFQ.GRVTITADESTRTAYMELSSLRSEDTAVYYC

B.1701 GGTF....SSYAISWVRQAPGQGLEWMGGIIPI..FGTANYAQKFQ.GRVTITADESTRTAYMELSSLRSEDTAVYYC

B.3121 GGTF....SSYAISWVRQAPGQGLEWMGGIIPI..FGTANYAQKFQ.GRVTITADESTRTAYMELSSLRSEDTAVYYC

B.991 GGTF....SSYAISWVRQAPGQGLEWIGGIIPI..FGTANYAQKFQ.GRVTITADESTSTAYMELSSLRSEDTAVYYC

B.1571 GGTF....SSYAISWVRQAPGQGLEWIGGIIPI..FGTANYAQKFQ.GRVTITADESTSTAYMELSSLRSEDTAVYYC

B.997 GGTF....STYAISWVRQAPGQGLEWMGGIIPI..FGTANYAQKFQ.GRVTITADESTSTAYMELSSLRSEDTAVYYC

B.1974 GGTF....STYAISWVRQAPGQGLEWMGGIIPI..FGTANYAQKFQ.GRVTITADESTSTAYMELSSLRSEDTAVYYC

B.1016 GGTF....SRYAISWVRQAPGQGLEWMGGIIPI..FGTANYAQKFQ.GRVTITADESTSTAYMELSSLRSEDTAVYYC

B.1017 GGTF....SSYAISWVRQAPGQGLEWMGGIIPI..FGTPNYAQKFQ.GRVTITADESTSTAYMELSSLRSEDTAVYYC

B.2090 GGTF....SSYAISWVRQAPGQGLEWMGGIIPI..FGTPNYAQKFQ.GRVTITADESTSTAYMELSSLRSEDTAVYYC

B.1068 GGTF....SNYAISWVRQAPGQGLEWMGGIIPI..FGTANYAQKFQ.GRVTITADESTSTAYMELSSLRSEDTAVYYC

B.1096 GGTF....SNYAISWVRQAPGQGLEWMGGIIPI..FGTANYAQKFQ.GRVTITADESTSTAYMELSSLRSEDTAVYYC

B.1982 GGTF....SNYAISWVRQAPGQGLEWMGGIIPI..FGTANYAQKFQ.GRVTITADESTSTAYMELSSLRSEDTAVYYC

B.2092 GGTF....SNYAISWVRQAPGQGLEWMGGIIPI..FGTANYAQKFQ.GRVTITADESTSTAYMELSSLRSEDTAVYYC

B.3625 GGTF....SNYAISWVRQAPGQGLEWMGGIIPI..FGTANYAQKFQ.GRVTITADESTSTAYMELSSLRSEDTAVYYC

B.1074 GGTF....SSYAISWVRHAPGQGLEWMGGIIPI..FGTANYAQKFQ.GRVTITADESTSTAYMELSSLRSEDTAVYYC

B.1089 GGTF....SNFAISWVRQAPGQGLEWMGGIIPI..FGTANYAQKFQ.GRVTITADKSTSTAYMELSSLRSEDTAVYYC

B.1098 GGTF....SSYAISWVRQAPGQGLEWMGGIIPI..FGTANYAQKFQ.GRVTITADESTSTAYMEVSSLRSEDTAVYYC

B.1853 GGTF....SSYAISWVRQAPGQGLEWMGGIIPI..FGTANYAQKFQ.GRVTITADESTSTAYMEVSSLRSEDTAVYYC

B.1131 GGTF....TSYAISWVRQAPGQGLEWMGGIIPI..FGTTNYAQKFQ.GRVTITADKSTSTAYMELSSLRSEDTAVYYC

B.1182 GGTF....SSYAISWVRQAPGQGLEWMGGIIPI..FGTANYAQKFQ.GRVTITADVSRSTAYMELSSLRSEDTAVYYC

B.1238 GGTF....SSYAIRWVRQAPGQGLEWMGGIIPI..FGTANYAQKFQ.GRVTITADESTSTAYMELSSLRSEDTAVYYC

B.1330 GGTF....SNYAISWVRQAPGQGLEWMGGIIPI..FGTTNYAQKFQ.GRVTITADKSTSTAYMELSSLRSEDTAVYYC

B.1346 GGTF....SRYAISWVRQAPGQGLEWMGGIIPF..FGTANYAQKFQ.GRVTITADKSTSTAYMELSSLRSEDTAVYYC

B.1435 .GTF....SSYAISWVRQAPGQGLEWMGGIIPI..FGTANYAQKFQ.GRVTITADESTSTAYMELSSLRSEDTAVYYC

B.1454 GGTF....SSYALSWVRQAPGQGLEWMGGIIPI..FGTANYAQKFQ.GRVT.TADKSTSTAYMELSSLRSEDTAVYYC

B.1470 GGTF....SSYAISWVRQAPGQGLEWMGGIIPI..FGTANYAQKFQ.GRVTITADKSTSTAYVELSSLRSEDTAFYYC

B.1520 GGTF....SSYAISWVRQAPGQGLEWMGWIIPI..FGTANYAQKFQ.GRVTITADESTSTAYMELSSLRSEDTAVYYC

B.1583 GGTF....SSYAISWVRQAPGQGLEWMGGIIPI..FGTANYAQKFQ.DRVKITADKSTSTAYMEVNSLRSEDTAVYYC

B.1593 GGTF....SSYAISWVRQAPGQGLEWMGGIIPI..FGTANYAHNCQ.GRVTITADKSTSTAYMELSSLRSEDTAVYYC

B.1601 GGTF....SSYAISWVRQAPGQGLEWMGGIIPI..FGTANYAQKFQ.GRVTITADKSTSTAYMELSSLTSDDTAVYYC

B.1622 GGTF....SSYAISWVRQAPGQGLEWMGGIIPI..IGTANYAQKFQ.GRVTITADESTSTAYMELSSLRSEDTAVYYC

B.1631 GGTF....SSSAISWVRQAPGQGLEWMGGIIPI..FGTANYAQKFQ.GRVTITADESTSTAYMELSSLRSEDTAVYYC

B.1671 GGTF....SSSAISWVRQAPGQGLEWMGGIIPI..FGTANYAQKFQ.GRVTITADESTSTAYMELSSLRSEDTAVYYC

B.1696 GGTF....SSSAISWVRQAPGQGLEWMGGIIPI..FGTANYAQKFQ.GRVTITADESTSTAYMELSSLRSEDTAVYYC

B.2733 GGTF....SSSAISWVRQAPGQGLEWMGGIIPI..FGTANYAQKFQ.GRVTITADESTSTAYMELSSLRSEDTAVYYC

B.1639 GGTF....SSYAISWVRQAPGQGLEWMGGIIPI..FGKANYAQKFQ.GRVTITADESTSTAYMELSSLRSEDTAVYYC

B.1653 GGTF....SSYAISWVRQAPGQGLEWMGGIIPI..FGTANYAQKFQ.GRVTITADESTSTVYMELSSLRSEDTAVYYC

B.1776 GGTF....SSYAISWVRQAPGQGLEWMGGIIPI..FGTANYAQKFQ.GRVTITADESTSTVYMELSSLRSEDTAVYYC

B.1717 GGTF....SSYAISWVRQAPGQGLEWVGGIIPI..FGTANYAQKFQ.GRVTITADESTSTAYMELSSLRSEDTAVYYC

B.1790 GGTF....SSYAISWVRQAPGQGLEWVGGIIPI..FGTANYAQKFQ.GRVTITADESTSTAYMELSSLRSEDTAVYYC

B.1869 GGTF....SSYAISWVRQAPGQGLEWVGGIIPI..FGTANYAQKFQ.GRVTITADESTSTAYMELSSLRSEDTAVYYC

B.1885 GGTF....SSYAISWVRQAPGQGLEWVGGIIPI..FGTANYAQKFQ.GRVTITADESTSTAYMELSSLRSEDTAVYYC

B.2700 GGTF....SSYAISWVRQAPGQGLEWVGGIIPI..FGTANYAQKFQ.GRVTITADESTSTAYMELSSLRSEDTAVYYC

B.1733 GGTF....SSYAISWVRQAPGQGLEWMGGIIPI..FGTANYAQKFQ.GRVTITADESTSTAYMELSSLRSEDTAVYFC

B.1768 GGTF....SSYAISWVRQAPGQGLEWMGGIIPI..FGTANYAQKFQ.GRVTITADESTSTAYMELTSLRSEDTAVYYC

B.1820 GGTF....SSYAISWVRQAPGQGLEWMGGIIPI..FGTANYAQKFQ.GRVTITADESTSTAYMELSSLRSEDTTVYYC

B.1832 GGTF....SSYAISWVRQAPGQGLEWMGGIIPI..FGTANYAQKFQ.GRVTITADESTSTAYMELSSLRPEDTAVYYC

B.1840 GGTF....SSYAISWVRQAPGQGLEWMGGIIPI..FGTANYAQNFQ.GRVTITADESTSTAYMELSSLRSEDTAVYYC

B.1858 GGTF....SSYAISWVRQAPGQGLEWMGGIIPI..FGTANYAQKFR.GRVTITADESTSTAYMELSSLRSEDTAVYYC

B.2032 GGTF....SSYAISWVRQAPGQGLEWMGGIIPI..FGTANYAQKFR.GRVTITADESTSTAYMELSSLRSEDTAVYYC

B.1861 GGTF....SSYAISWVRQAPGQGLEWMGGIIPI..FGTANYAQKFQ.GRVTITADESTSTAYMELSSRRSEDTAVYYC

B.1886 GGTF....SSYAISWVRQAPGQGLEWMGGIIPI..FGTANYAQKFQ.GRVTITADESTSTAYMELSSLRSEDTAVFYC

B.1892 GGTF....SSYAISWVRQAPGQGLEWMGGIIPI..FGTANYAQKFQ.GRVTITADESTSTAYMELSSLRSDDTAVYYC

B.3587 GGTF....SSYAISWVRQAPGQGLEWMGGIIPI..FGTANYAQKFQ.GRVTITADESTSTAYMELSSLRSDDTAVYYC

B.1933 GGTF....SSYAISWVRQAPGQGLEWMGGIIPI..FGTTNYAQKFQ.GRVTITADESTSTAYMELSSLRSEDTAVYYC

B.2902 GGTF....SSYAISWVRQAPGQGLEWMGGIIPI..FGTTNYAQKFQ.GRVTITADESTSTAYMELSSLRSEDTAVYYC

B.1984 GGTF....SSYAISWVRQAPGQGLEWMGGIIPI..FGTANYAQKFQ.GRVTITADESTSTAYLELSSLRSEDTAVYYC

B.2021 GGTF....RSYAISWVRQAPGQGLEWMGGIIPI..FGTANYAQKFQ.GRVTITADESTSTAYMELSSLRSEDTAVYYC

B.2056 GGTF....SNYAISWVRQAPGQGLEWMGRIIPI..FGTANYAQKFQ.GRVTITADKSTSTAYMELSSLRSEDTAVYYC

B.2077 GGTF....SSYAISCVRQAPGQGLEWMGGIIPI..FGTANYAQKFQ.GRVTITADESTSTAYMELSSLRSEDTAVYYC

B.2094 GGTF....SSYAISWVRQAPGQGLEWMGGIIPI..FGTAKYAQKFQ.GRVTITADESTSTAYMELSSLRSEDTAVYYC

B.2276 GGTF....SSYAISWVRQAPGQGLEWMGGIIPI..FGTANYAQKFQ.GRVTITADESTSTAYMELSSLRSEDSAVYYC

B.2295 GGTF....SSYAISWVRQVPGQGLEWMGGIIPI..FGTANYAQRFQ.GRVTITADKSTSTAYMELSSLRSEDTAVYYC

B.2358 GGTF....SSYAISWVRQAPGQGLEWMGGIIPI..FGTANSAQKFQ.GRVTITADKSTSTVYMELSSLRSEDTAVYYC

B.2359 GGTF....SSYAISWVRQAPGQGLEWMGGIIPI..LGTANYAQKFQ.GRVTITADESTSTAYMELSSLRSEDTAVYYC

B.2470 GGTF....SSYAISWVRQAPGQGLEWMGGIIPI..FGTANYAQKFQ.GRVTITADASTSTAYMDLSSLRSEDTAVYYC

B.2622 GGTF....SGYAISWVRQAPGQGLEWMGGIIPI..FGTANYAQKFQ.GRVTITADESTSTAYMELSSLRSEDTAVYYC

B.2636 GGTF....SSYAISWVRQAPGQGLEWMGGIIPI..FGIANYAQKFQ.GRVTITADKSTSTAYMELRSLRSEDTAVYYC

B.2679 GGTF....SSYAISWVRQAPGQGLEWMGGIIPM..FGTANYAQKFQ.GRVTITADESTSTAYMELSSLRSEDTAVYYC

B.2720 GGTF....SSYAISWVRQAPGQGLEWMGGIIPI..FGTANYAQKFQ.GRVTITADESTTTAYMELSSLRSEDTAVYYC

B.2761 GGTF....SNYAFSWVRQAPGQGLEWMGGIIPI..FGTANYAQKFQ.GRVTITADKSTSTAYMELSSLRSEDTAVYYC

B.2765 GGTF....SNYAISWVRQAPGQGLEWMGGIIPI..FGTANYAQKFQ.GRVTITADKSTSTAYMELSSLTSEDTAVYYC

B.2783 GGTF....SNYAISWVRQAPGQGLEWMGGIIPI..FGTANYAQKFQ.GRVTITADKSTSTAYMELSSLTSEDTAVYYC

B.2767 GGTF....STYAISWVRQAPGQGLEWMGGIIPI..FGTANYAQKFQ.GRVTITADKSTSTAYMELSSLTSEDTAVYYC

B.2768 GGTF....GSHAISWVRQAPGQGLEWMGGIIPI..FGTANYAQKFQ.DRVTITADKSTSTAYMELSSLTSEDTAVYYC

B.2822 GGTF....GSHAISWVRQAPGQGLEWMGGIIPI..FGTANYAQKFQ.DRVTITADKSTSTAYMELSSLTSEDTAVYYC

B.2800 GGTF....SSYAISWVRQAPGQGLEWMGGIIPI..FGTANYAQKFQ.GRVTITADKSTNTAYMELSSLTSEDTAVYYC

B.2816 GGTF....SSYAINWVRQAPGQGLEWMGGIIPI..FGTANYAQKFQ.GRVTITADESTSTAYMELSSLRSEDTAVYYC

B.2823 GDTF....SSYAISWVRQAPGQGLEWMGGIIPI..FGTGNYAQKFQ.GRVTITADKSTSTAYMELSSLRSEDTAVYYC

B.2825 GGTF....SSYAISWVRQAPGQGLEWMGGIIPI..FGTANYAQKFQ.GRVTITADTSTSTVYMELSSLRSEDTAVYYC

B.2833 GGTF....STYAISWVRQAPGQGLEWMGGIIPI..FGTANYAQKFQ.GRVTITADKSTRTAYMELSSLRSEDTAVYYC

B.2834 GGTF....STYAISWVRQAPGQGLEWMGGIIPI..FGTANYAQKFQ.GRVTIAADKSTSTAYMELSSLRSEDTAVYYC

B.2844 GGTF....SSYAISWVRQAPGQGLEWMGGIIPI..FGTANYAQKFQ.GRATITADKSTTTAYMELSSLRSEDTAVYYC

B.3096 GGTF....SSYAISWVRQAPGQGLEWMGGIIPI..FGTANYAQKFQ.GRVTITSDESTSTAYMELSSLRSEDTAVYYC

B.3126 GGTF....SSYPISWVRQAPGQGLEWMGGIIPI..FGTANYAQKFQ.GRVTITADESTSTAYMELSSLRSEDTAVYYC

B.3135 GGTF....SSYAISWVRQAPGQGLEWMGGIIPI..FGTANYAQKFQ.GRVTI.ADKSTSTAYMELRSLRSEDTAVYYC

B.3138 GGTF....SSYSISWVRQAPGQGLEWMGGIIPF..FGTADYAQKFQ.DRVTITADKSTSTAYMELSSLRSEDTAVYYC

B.3162 GGTF....SSYAISWVRQAPGQGLEWMGGIIPI..FGTANYAQKFQ.GRVTITADESTSTAYMELRSLRSEDTAVYYC

B.3186 GGTF....SSYAISWVRQAPGQGLEWMGGIIPI..FGTANYALKFQ.GRVTITADESTSTAYMELSSLRSEDTAVYYC

B.3191 GGTC....SSYAISWVRQAPGQGLEWMGGIIPI..FGTANYAQKFQ.GRVTITADESTSTAYMELSSLRSEDTAVYYC

B.3267 GGTF....SNYAITWVRQAPGQGLEWMGGIIPI..FGTANYAQKFQ.GRVTITADKSTSTAYMELSSLRSEDTAVYYC

B.3290 GGTF....SSYAISWVRQAPGQGLEWMGGIIPI..FGTANYAQKFQ.GRVTITADESTSTAYMELSSLRSEDTAVYHC

B.3381 GGTF....SSYAISWVRQAPGQGLEWMGGIIPI..FGTANYAQKFL.GRVTITADASTSTAYMELSSLRSEDTAVYYC

B.3430 GDTF....SSYAISWVRQAPGQGLEWMGGIIPI..FGTANYAQKFQ.GRVTITADESTSTAYMELSSLRSEDTAVYYC

B.3437 GGTF....SSYAISWVRQAPGQGLEWMGGIIPI..FGTANYAQKFQ.GRVTITADESTSTAYMELSRLRSEDTAVYYC

B.3454 GGTF....SSYAISWVRQAPGQGLEWMGGIIPI..FGIANYAQKFQ.GRVTITADESTSTAYMELSSLRSEDTAVYYC

B.3462 GGTF....SSYAISWVRQAPGQGLEWMGGIIPI..FGIANYAQKFQ.GRVTITADESTSTAYMELSSLRSEDTAVYYC

B.3498 GGTF....SSYAISWVRQAPGQGLEWMGGIIPI..FGIANYAQKFQ.GRVTITADESTSTAYMELSSLRSEDTAVYYC

B.3503 GGTF....SSYAISWVRQAPGQGLEWMGGIIPI..FGIANYAQKFQ.GRVTITADESTSTAYMELSSLRSEDTAVYYC

B.3504 GGTF....SSYAISWVRQAPGQGLEWMGGIIPI..FGIANYAQKFQ.GRVTITADESTSTAYMELSSLRSEDTAVYYC

B.3479 GGTF....SNYAISWVRQAAGQGLEWMGGIIPI..FGTANYAQKFQ.GRVTITADKSTSTAYMELSSLRSEDTAVYYC

B.3514 GGTF....SSYAISWVRQAPGQGLEWMGGIIPI..FGTANYVQKFQ.GRVTITADKSTNTAYMELSSLRSEDTAVYYC

B.3530 GGTF....SSYAISWVRQAPGQGLEWMGGIIPI..FGTANYAQKFQ.GRVTITADEFTSTAYMELSSLRSEDTAVYYC

B.3562 GGTF....SSYAISWVRQAPGQGLEWMGGIIPI..FGTANYAQKFQ.GRVKITADESTSTAYMELSSLRSEDTAVYYC

**D. Alignment with up to five exchanges________________________________________**

Weber et. al Sequences 1198_01_E11 and 1198_03_G07

GGTF....SSYAISWVRQAPGQGLEWMGGIIPI..FGTANYAQKLQ.DRVTITADKSTSTAYMELSSLRSEDTAVYYC

Aligned Sequences (n=327) from our cohort

B.3 GGTF....SSYAISWVRQAPGQGLEWMGRIIPI..LGIANYAQKFQ.GRVTITADKSTSTAYMELSSLRSEDTAVYYC

B.5 GGTF....SSYAISWVRQAPGQGLEWMGRIIPI..LGIANYAQKFQ.GRVTITADKSTSTAYMELSSLRSEDTAVYYC

B.7 GGTF....SSYAISWVRQAPGQGLEWMGRIIPI..LGIANYAQKFQ.GRVTITADKSTSTAYMELSSLRSEDTAVYYC

B.11 GGTF....SSYAISWVRQAPGQGLEWMGRIIPI..LGIANYAQKFQ.GRVTITADKSTSTAYMELSSLRSEDTAVYYC

B.23 GGTF....SSYAISWVRQAPGQGLEWMGRIIPI..LGIANYAQKFQ.GRVTITADKSTSTAYMELSSLRSEDTAVYYC

B.25 GGTF....SSYAISWVRQAPGQGLEWMGRIIPI..LGIANYAQKFQ.GRVTITADKSTSTAYMELSSLRSEDTAVYYC

B.647 GGTF....SSYAISWVRQAPGQGLEWMGRIIPI..LGIANYAQKFQ.GRVTITADKSTSTAYMELSSLRSEDTAVYYC

B.657 GGTF....SSYAISWVRQAPGQGLEWMGRIIPI..LGIANYAQKFQ.GRVTITADKSTSTAYMELSSLRSEDTAVYYC

B.671 GGTF....SSYAISWVRQAPGQGLEWMGRIIPI..LGIANYAQKFQ.GRVTITADKSTSTAYMELSSLRSEDTAVYYC

B.755 GGTF....SSYAISWVRQAPGQGLEWMGRIIPI..LGIANYAQKFQ.GRVTITADKSTSTAYMELSSLRSEDTAVYYC

B.756 GGTF....SSYAISWVRQAPGQGLEWMGRIIPI..LGIANYAQKFQ.GRVTITADKSTSTAYMELSSLRSEDTAVYYC

B.763 GGTF....SSYAISWVRQAPGQGLEWMGRIIPI..LGIANYAQKFQ.GRVTITADKSTSTAYMELSSLRSEDTAVYYC

B.769 GGTF....SSYAISWVRQAPGQGLEWMGRIIPI..LGIANYAQKFQ.GRVTITADKSTSTAYMELSSLRSEDTAVYYC

B.773 GGTF....SSYAISWVRQAPGQGLEWMGRIIPI..LGIANYAQKFQ.GRVTITADKSTSTAYMELSSLRSEDTAVYYC

B.776 GGTF....SSYAISWVRQAPGQGLEWMGRIIPI..LGIANYAQKFQ.GRVTITADKSTSTAYMELSSLRSEDTAVYYC

B.777 GGTF....SSYAISWVRQAPGQGLEWMGRIIPI..LGIANYAQKFQ.GRVTITADKSTSTAYMELSSLRSEDTAVYYC

B.778 GGTF....SSYAISWVRQAPGQGLEWMGRIIPI..LGIANYAQKFQ.GRVTITADKSTSTAYMELSSLRSEDTAVYYC

B.779 GGTF....SSYAISWVRQAPGQGLEWMGRIIPI..LGIANYAQKFQ.GRVTITADKSTSTAYMELSSLRSEDTAVYYC

B.780 GGTF....SSYAISWVRQAPGQGLEWMGRIIPI..LGIANYAQKFQ.GRVTITADKSTSTAYMELSSLRSEDTAVYYC

B.783 GGTF....SSYAISWVRQAPGQGLEWMGRIIPI..LGIANYAQKFQ.GRVTITADKSTSTAYMELSSLRSEDTAVYYC

B.788 GGTF....SSYAISWVRQAPGQGLEWMGRIIPI..LGIANYAQKFQ.GRVTITADKSTSTAYMELSSLRSEDTAVYYC

B.790 GGTF....SSYAISWVRQAPGQGLEWMGRIIPI..LGIANYAQKFQ.GRVTITADKSTSTAYMELSSLRSEDTAVYYC

B.792 GGTF....SSYAISWVRQAPGQGLEWMGRIIPI..LGIANYAQKFQ.GRVTITADKSTSTAYMELSSLRSEDTAVYYC

B.801 GGTF....SSYAISWVRQAPGQGLEWMGRIIPI..LGIANYAQKFQ.GRVTITADKSTSTAYMELSSLRSEDTAVYYC

B.804 GGTF....SSYAISWVRQAPGQGLEWMGRIIPI..LGIANYAQKFQ.GRVTITADKSTSTAYMELSSLRSEDTAVYYC

B.806 GGTF....SSYAISWVRQAPGQGLEWMGRIIPI..LGIANYAQKFQ.GRVTITADKSTSTAYMELSSLRSEDTAVYYC

B.807 GGTF....SSYAISWVRQAPGQGLEWMGRIIPI..LGIANYAQKFQ.GRVTITADKSTSTAYMELSSLRSEDTAVYYC

B.818 GGTF....SSYAISWVRQAPGQGLEWMGRIIPI..LGIANYAQKFQ.GRVTITADKSTSTAYMELSSLRSEDTAVYYC

B.823 GGTF....SSYAISWVRQAPGQGLEWMGRIIPI..LGIANYAQKFQ.GRVTITADKSTSTAYMELSSLRSEDTAVYYC

B.824 GGTF....SSYAISWVRQAPGQGLEWMGRIIPI..LGIANYAQKFQ.GRVTITADKSTSTAYMELSSLRSEDTAVYYC

B.825 GGTF....SSYAISWVRQAPGQGLEWMGRIIPI..LGIANYAQKFQ.GRVTITADKSTSTAYMELSSLRSEDTAVYYC

B.829 GGTF....SSYAISWVRQAPGQGLEWMGRIIPI..LGIANYAQKFQ.GRVTITADKSTSTAYMELSSLRSEDTAVYYC

B.830 GGTF....SSYAISWVRQAPGQGLEWMGRIIPI..LGIANYAQKFQ.GRVTITADKSTSTAYMELSSLRSEDTAVYYC

B.831 GGTF....SSYAISWVRQAPGQGLEWMGRIIPI..LGIANYAQKFQ.GRVTITADKSTSTAYMELSSLRSEDTAVYYC

B.832 GGTF....SSYAISWVRQAPGQGLEWMGRIIPI..LGIANYAQKFQ.GRVTITADKSTSTAYMELSSLRSEDTAVYYC

B.838 GGTF....SSYAISWVRQAPGQGLEWMGRIIPI..LGIANYAQKFQ.GRVTITADKSTSTAYMELSSLRSEDTAVYYC

B.839 GGTF....SSYAISWVRQAPGQGLEWMGRIIPI..LGIANYAQKFQ.GRVTITADKSTSTAYMELSSLRSEDTAVYYC

B.1357 GGTF....SSYAISWVRQAPGQGLEWMGRIIPI..LGIANYAQKFQ.GRVTITADKSTSTAYMELSSLRSEDTAVYYC

B.1446 GGTF....SSYAISWVRQAPGQGLEWMGRIIPI..LGIANYAQKFQ.GRVTITADKSTSTAYMELSSLRSEDTAVYYC

B.1489 GGTF....SSYAISWVRQAPGQGLEWMGRIIPI..LGIANYAQKFQ.GRVTITADKSTSTAYMELSSLRSEDTAVYYC

B.1513 GGTF....SSYAISWVRQAPGQGLEWMGRIIPI..LGIANYAQKFQ.GRVTITADKSTSTAYMELSSLRSEDTAVYYC

B.1514 GGTF....SSYAISWVRQAPGQGLEWMGRIIPI..LGIANYAQKFQ.GRVTITADKSTSTAYMELSSLRSEDTAVYYC

B.1542 GGTF....SSYAISWVRQAPGQGLEWMGRIIPI..LGIANYAQKFQ.GRVTITADKSTSTAYMELSSLRSEDTAVYYC

B.1544 GGTF....SSYAISWVRQAPGQGLEWMGRIIPI..LGIANYAQKFQ.GRVTITADKSTSTAYMELSSLRSEDTAVYYC

B.1546 GGTF....SSYAISWVRQAPGQGLEWMGRIIPI..LGIANYAQKFQ.GRVTITADKSTSTAYMELSSLRSEDTAVYYC

B.1959 GGTF....SSYAISWVRQAPGQGLEWMGRIIPI..LGIANYAQKFQ.GRVTITADKSTSTAYMELSSLRSEDTAVYYC

B.1968 GGTF....SSYAISWVRQAPGQGLEWMGRIIPI..LGIANYAQKFQ.GRVTITADKSTSTAYMELSSLRSEDTAVYYC

B.1990 GGTF....SSYAISWVRQAPGQGLEWMGRIIPI..LGIANYAQKFQ.GRVTITADKSTSTAYMELSSLRSEDTAVYYC

B.1993 GGTF....SSYAISWVRQAPGQGLEWMGRIIPI..LGIANYAQKFQ.GRVTITADKSTSTAYMELSSLRSEDTAVYYC

B.2001 GGTF....SSYAISWVRQAPGQGLEWMGRIIPI..LGIANYAQKFQ.GRVTITADKSTSTAYMELSSLRSEDTAVYYC

B.2020 GGTF....SSYAISWVRQAPGQGLEWMGRIIPI..LGIANYAQKFQ.GRVTITADKSTSTAYMELSSLRSEDTAVYYC

B.2034 GGTF....SSYAISWVRQAPGQGLEWMGRIIPI..LGIANYAQKFQ.GRVTITADKSTSTAYMELSSLRSEDTAVYYC

B.2053 GGTF....SSYAISWVRQAPGQGLEWMGRIIPI..LGIANYAQKFQ.GRVTITADKSTSTAYMELSSLRSEDTAVYYC

B.2119 GGTF....SSYAISWVRQAPGQGLEWMGRIIPI..LGIANYAQKFQ.GRVTITADKSTSTAYMELSSLRSEDTAVYYC

B.2120 GGTF....SSYAISWVRQAPGQGLEWMGRIIPI..LGIANYAQKFQ.GRVTITADKSTSTAYMELSSLRSEDTAVYYC

B.2123 GGTF....SSYAISWVRQAPGQGLEWMGRIIPI..LGIANYAQKFQ.GRVTITADKSTSTAYMELSSLRSEDTAVYYC

B.2132 GGTF....SSYAISWVRQAPGQGLEWMGRIIPI..LGIANYAQKFQ.GRVTITADKSTSTAYMELSSLRSEDTAVYYC

B.2186 GGTF....SSYAISWVRQAPGQGLEWMGRIIPI..LGIANYAQKFQ.GRVTITADKSTSTAYMELSSLRSEDTAVYYC

B.2410 GGTF....SSYAISWVRQAPGQGLEWMGRIIPI..LGIANYAQKFQ.GRVTITADKSTSTAYMELSSLRSEDTAVYYC

B.2415 GGTF....SSYAISWVRQAPGQGLEWMGRIIPI..LGIANYAQKFQ.GRVTITADKSTSTAYMELSSLRSEDTAVYYC

B.2420 GGTF....SSYAISWVRQAPGQGLEWMGRIIPI..LGIANYAQKFQ.GRVTITADKSTSTAYMELSSLRSEDTAVYYC

B.2428 GGTF....SSYAISWVRQAPGQGLEWMGRIIPI..LGIANYAQKFQ.GRVTITADKSTSTAYMELSSLRSEDTAVYYC

B.2432 GGTF....SSYAISWVRQAPGQGLEWMGRIIPI..LGIANYAQKFQ.GRVTITADKSTSTAYMELSSLRSEDTAVYYC

B.2436 GGTF....SSYAISWVRQAPGQGLEWMGRIIPI..LGIANYAQKFQ.GRVTITADKSTSTAYMELSSLRSEDTAVYYC

B.2439 GGTF....SSYAISWVRQAPGQGLEWMGRIIPI..LGIANYAQKFQ.GRVTITADKSTSTAYMELSSLRSEDTAVYYC

B.2441 GGTF....SSYAISWVRQAPGQGLEWMGRIIPI..LGIANYAQKFQ.GRVTITADKSTSTAYMELSSLRSEDTAVYYC

B.2443 GGTF....SSYAISWVRQAPGQGLEWMGRIIPI..LGIANYAQKFQ.GRVTITADKSTSTAYMELSSLRSEDTAVYYC

B.2444 GGTF....SSYAISWVRQAPGQGLEWMGRIIPI..LGIANYAQKFQ.GRVTITADKSTSTAYMELSSLRSEDTAVYYC

B.2449 GGTF....SSYAISWVRQAPGQGLEWMGRIIPI..LGIANYAQKFQ.GRVTITADKSTSTAYMELSSLRSEDTAVYYC

B.2451 GGTF....SSYAISWVRQAPGQGLEWMGRIIPI..LGIANYAQKFQ.GRVTITADKSTSTAYMELSSLRSEDTAVYYC

B.2453 GGTF....SSYAISWVRQAPGQGLEWMGRIIPI..LGIANYAQKFQ.GRVTITADKSTSTAYMELSSLRSEDTAVYYC

B.2456 GGTF....SSYAISWVRQAPGQGLEWMGRIIPI..LGIANYAQKFQ.GRVTITADKSTSTAYMELSSLRSEDTAVYYC

B.2462 GGTF....SSYAISWVRQAPGQGLEWMGRIIPI..LGIANYAQKFQ.GRVTITADKSTSTAYMELSSLRSEDTAVYYC

B.2463 GGTF....SSYAISWVRQAPGQGLEWMGRIIPI..LGIANYAQKFQ.GRVTITADKSTSTAYMELSSLRSEDTAVYYC

B.2505 GGTF....SSYAISWVRQAPGQGLEWMGRIIPI..LGIANYAQKFQ.GRVTITADKSTSTAYMELSSLRSEDTAVYYC

B.2507 GGTF....SSYAISWVRQAPGQGLEWMGRIIPI..LGIANYAQKFQ.GRVTITADKSTSTAYMELSSLRSEDTAVYYC

B.2512 GGTF....SSYAISWVRQAPGQGLEWMGRIIPI..LGIANYAQKFQ.GRVTITADKSTSTAYMELSSLRSEDTAVYYC

B.2515 GGTF....SSYAISWVRQAPGQGLEWMGRIIPI..LGIANYAQKFQ.GRVTITADKSTSTAYMELSSLRSEDTAVYYC

B.2528 GGTF....SSYAISWVRQAPGQGLEWMGRIIPI..LGIANYAQKFQ.GRVTITADKSTSTAYMELSSLRSEDTAVYYC

B.2530 GGTF....SSYAISWVRQAPGQGLEWMGRIIPI..LGIANYAQKFQ.GRVTITADKSTSTAYMELSSLRSEDTAVYYC

B.2534 GGTF....SSYAISWVRQAPGQGLEWMGRIIPI..LGIANYAQKFQ.GRVTITADKSTSTAYMELSSLRSEDTAVYYC

B.2536 GGTF....SSYAISWVRQAPGQGLEWMGRIIPI..LGIANYAQKFQ.GRVTITADKSTSTAYMELSSLRSEDTAVYYC

B.2537 GGTF....SSYAISWVRQAPGQGLEWMGRIIPI..LGIANYAQKFQ.GRVTITADKSTSTAYMELSSLRSEDTAVYYC

B.2865 GGTF....SSYAISWVRQAPGQGLEWMGRIIPI..LGIANYAQKFQ.GRVTITADKSTSTAYMELSSLRSEDTAVYYC

B.2866 GGTF....SSYAISWVRQAPGQGLEWMGRIIPI..LGIANYAQKFQ.GRVTITADKSTSTAYMELSSLRSEDTAVYYC

B.2868 GGTF....SSYAISWVRQAPGQGLEWMGRIIPI..LGIANYAQKFQ.GRVTITADKSTSTAYMELSSLRSEDTAVYYC

B.2872 GGTF....SSYAISWVRQAPGQGLEWMGRIIPI..LGIANYAQKFQ.GRVTITADKSTSTAYMELSSLRSEDTAVYYC

B.2874 GGTF....SSYAISWVRQAPGQGLEWMGRIIPI..LGIANYAQKFQ.GRVTITADKSTSTAYMELSSLRSEDTAVYYC

B.2885 GGTF....SSYAISWVRQAPGQGLEWMGRIIPI..LGIANYAQKFQ.GRVTITADKSTSTAYMELSSLRSEDTAVYYC

B.2889 GGTF....SSYAISWVRQAPGQGLEWMGRIIPI..LGIANYAQKFQ.GRVTITADKSTSTAYMELSSLRSEDTAVYYC

B.2952 GGTF....SSYAISWVRQAPGQGLEWMGRIIPI..LGIANYAQKFQ.GRVTITADKSTSTAYMELSSLRSEDTAVYYC

B.3034 GGTF....SSYAISWVRQAPGQGLEWMGRIIPI..LGIANYAQKFQ.GRVTITADKSTSTAYMELSSLRSEDTAVYYC

B.3035 GGTF....SSYAISWVRQAPGQGLEWMGRIIPI..LGIANYAQKFQ.GRVTITADKSTSTAYMELSSLRSEDTAVYYC

B.3036 GGTF....SSYAISWVRQAPGQGLEWMGRIIPI..LGIANYAQKFQ.GRVTITADKSTSTAYMELSSLRSEDTAVYYC

B.3038 GGTF....SSYAISWVRQAPGQGLEWMGRIIPI..LGIANYAQKFQ.GRVTITADKSTSTAYMELSSLRSEDTAVYYC

B.3041 GGTF....SSYAISWVRQAPGQGLEWMGRIIPI..LGIANYAQKFQ.GRVTITADKSTSTAYMELSSLRSEDTAVYYC

B.3042 GGTF....SSYAISWVRQAPGQGLEWMGRIIPI..LGIANYAQKFQ.GRVTITADKSTSTAYMELSSLRSEDTAVYYC

B.3044 GGTF....SSYAISWVRQAPGQGLEWMGRIIPI..LGIANYAQKFQ.GRVTITADKSTSTAYMELSSLRSEDTAVYYC

B.3045 GGTF....SSYAISWVRQAPGQGLEWMGRIIPI..LGIANYAQKFQ.GRVTITADKSTSTAYMELSSLRSEDTAVYYC

B.3046 GGTF....SSYAISWVRQAPGQGLEWMGRIIPI..LGIANYAQKFQ.GRVTITADKSTSTAYMELSSLRSEDTAVYYC

B.3048 GGTF....SSYAISWVRQAPGQGLEWMGRIIPI..LGIANYAQKFQ.GRVTITADKSTSTAYMELSSLRSEDTAVYYC

B.3057 GGTF....SSYAISWVRQAPGQGLEWMGRIIPI..LGIANYAQKFQ.GRVTITADKSTSTAYMELSSLRSEDTAVYYC

B.3060 GGTF....SSYAISWVRQAPGQGLEWMGRIIPI..LGIANYAQKFQ.GRVTITADKSTSTAYMELSSLRSEDTAVYYC

B.3273 GGTF....SSYAISWVRQAPGQGLEWMGRIIPI..LGIANYAQKFQ.GRVTITADKSTSTAYMELSSLRSEDTAVYYC

B.3299 GGTF....SSYAISWVRQAPGQGLEWMGRIIPI..LGIANYAQKFQ.GRVTITADKSTSTAYMELSSLRSEDTAVYYC

B.3302 GGTF....SSYAISWVRQAPGQGLEWMGRIIPI..LGIANYAQKFQ.GRVTITADKSTSTAYMELSSLRSEDTAVYYC

B.3317 GGTF....SSYAISWVRQAPGQGLEWMGRIIPI..LGIANYAQKFQ.GRVTITADKSTSTAYMELSSLRSEDTAVYYC

B.3318 GGTF....SSYAISWVRQAPGQGLEWMGRIIPI..LGIANYAQKFQ.GRVTITADKSTSTAYMELSSLRSEDTAVYYC

B.3330 GGTF....SSYAISWVRQAPGQGLEWMGRIIPI..LGIANYAQKFQ.GRVTITADKSTSTAYMELSSLRSEDTAVYYC

B.3342 GGTF....SSYAISWVRQAPGQGLEWMGRIIPI..LGIANYAQKFQ.GRVTITADKSTSTAYMELSSLRSEDTAVYYC

B.3345 GGTF....SSYAISWVRQAPGQGLEWMGRIIPI..LGIANYAQKFQ.GRVTITADKSTSTAYMELSSLRSEDTAVYYC

B.3350 GGTF....SSYAISWVRQAPGQGLEWMGRIIPI..LGIANYAQKFQ.GRVTITADKSTSTAYMELSSLRSEDTAVYYC

B.3351 GGTF....SSYAISWVRQAPGQGLEWMGRIIPI..LGIANYAQKFQ.GRVTITADKSTSTAYMELSSLRSEDTAVYYC

B.3356 GGTF....SSYAISWVRQAPGQGLEWMGRIIPI..LGIANYAQKFQ.GRVTITADKSTSTAYMELSSLRSEDTAVYYC

B.3357 GGTF....SSYAISWVRQAPGQGLEWMGRIIPI..LGIANYAQKFQ.GRVTITADKSTSTAYMELSSLRSEDTAVYYC

B.3359 GGTF....SSYAISWVRQAPGQGLEWMGRIIPI..LGIANYAQKFQ.GRVTITADKSTSTAYMELSSLRSEDTAVYYC

B.3362 GGTF....SSYAISWVRQAPGQGLEWMGRIIPI..LGIANYAQKFQ.GRVTITADKSTSTAYMELSSLRSEDTAVYYC

B.3364 GGTF....SSYAISWVRQAPGQGLEWMGRIIPI..LGIANYAQKFQ.GRVTITADKSTSTAYMELSSLRSEDTAVYYC

B.3367 GGTF....SSYAISWVRQAPGQGLEWMGRIIPI..LGIANYAQKFQ.GRVTITADKSTSTAYMELSSLRSEDTAVYYC

B.3370 GGTF....SSYAISWVRQAPGQGLEWMGRIIPI..LGIANYAQKFQ.GRVTITADKSTSTAYMELSSLRSEDTAVYYC

B.3380 GGTF....SSYAISWVRQAPGQGLEWMGRIIPI..LGIANYAQKFQ.GRVTITADKSTSTAYMELSSLRSEDTAVYYC

B.3382 GGTF....SSYAISWVRQAPGQGLEWMGRIIPI..LGIANYAQKFQ.GRVTITADKSTSTAYMELSSLRSEDTAVYYC

B.3431 GGTF....SSYAISWVRQAPGQGLEWMGRIIPI..LGIANYAQKFQ.GRVTITADKSTSTAYMELSSLRSEDTAVYYC

B.3432 GGTF....SSYAISWVRQAPGQGLEWMGRIIPI..LGIANYAQKFQ.GRVTITADKSTSTAYMELSSLRSEDTAVYYC

B.3440 GGTF....SSYAISWVRQAPGQGLEWMGRIIPI..LGIANYAQKFQ.GRVTITADKSTSTAYMELSSLRSEDTAVYYC

B.3507 GGTF....SSYAISWVRQAPGQGLEWMGRIIPI..LGIANYAQKFQ.GRVTITADKSTSTAYMELSSLRSEDTAVYYC

B.3508 GGTF....SSYAISWVRQAPGQGLEWMGRIIPI..LGIANYAQKFQ.GRVTITADKSTSTAYMELSSLRSEDTAVYYC

B.3510 GGTF....SSYAISWVRQAPGQGLEWMGRIIPI..LGIANYAQKFQ.GRVTITADKSTSTAYMELSSLRSEDTAVYYC

B.3524 GGTF....SSYAISWVRQAPGQGLEWMGRIIPI..LGIANYAQKFQ.GRVTITADKSTSTAYMELSSLRSEDTAVYYC

B.3526 GGTF....SSYAISWVRQAPGQGLEWMGRIIPI..LGIANYAQKFQ.GRVTITADKSTSTAYMELSSLRSEDTAVYYC

B.3532 GGTF....SSYAISWVRQAPGQGLEWMGRIIPI..LGIANYAQKFQ.GRVTITADKSTSTAYMELSSLRSEDTAVYYC

B.35 GGTF....NNYAISWVRQAPGQGLEWMGGIIPI..FGTANYAQKFQ.GRVTISADKSTSTAYMELSSLRSEDTAVYYC

B.42 GGTF....SSYGINWVRQAPGQGLEWMGGIIPI..FGTANYAQKFQ.SRVTITADKSTSTPYMELSSLRSEDTAVYYC

B.192 GGTF....STYAISWVRQAPGQGLEWMGGIIPI..FGTANYAQKFQ.GRVTITADTSTSTAYMELSSLTSEDTAVYYC

B.203 GGTF....STYAISWVRQAPGQGLEWMGGIIPI..FGTANYAQKFQ.GRVTITADTSTSTAYMELSSLTSEDTAVYYC

B.204 GGTF....STYAISWVRQAPGQGLEWMGGIIPI..FGTANYAQKFQ.GRVTITADTSTSTAYMELSSLTSEDTAVYYC

B.211 GGTF....STYAISWVRQAPGQGLEWMGGIIPI..FGTANYAQKFQ.GRVTITADTSTSTAYMELSSLTSEDTAVYYC

B.215 GGTF....STYAISWVRQAPGQGLEWMGGIIPI..FGTANYAQKFQ.GRVTITADTSTSTAYMELSSLTSEDTAVYYC

B.232 GGTF....STYAISWVRQAPGQGLEWMGGIIPI..FGTANYAQKFQ.GRVTITADTSTSTAYMELSSLTSEDTAVYYC

B.246 GGTF....STYAISWVRQAPGQGLEWMGGIIPI..FGTANYAQKFQ.GRVTITADTSTSTAYMELSSLTSEDTAVYYC

B.253 GGTF....STYAISWVRQAPGQGLEWMGGIIPI..FGTANYAQKFQ.GRVTITADTSTSTAYMELSSLTSEDTAVYYC

B.255 GGTF....STYAISWVRQAPGQGLEWMGGIIPI..FGTANYAQKFQ.GRVTITADTSTSTAYMELSSLTSEDTAVYYC

B.261 GGTF....STYAISWVRQAPGQGLEWMGGIIPI..FGTANYAQKFQ.GRVTITADTSTSTAYMELSSLTSEDTAVYYC

B.266 GGTF....STYAISWVRQAPGQGLEWMGGIIPI..FGTANYAQKFQ.GRVTITADTSTSTAYMELSSLTSEDTAVYYC

B.291 GGTF....STYAISWVRQAPGQGLEWMGGIIPI..FGTANYAQKFQ.GRVTITADTSTSTAYMELSSLTSEDTAVYYC

B.309 GGTF....STYAISWVRQAPGQGLEWMGGIIPI..FGTANYAQKFQ.GRVTITADTSTSTAYMELSSLTSEDTAVYYC

B.344 GGTF....STYAISWVRQAPGQGLEWMGGIIPI..FGTANYAQKFQ.GRVTITADTSTSTAYMELSSLTSEDTAVYYC

B.2766 GGTF....STYAISWVRQAPGQGLEWMGGIIPI..FGTANYAQKFQ.GRVTITADTSTSTAYMELSSLTSEDTAVYYC

B.2847 GGTF....STYAISWVRQAPGQGLEWMGGIIPI..FGTANYAQKFQ.GRVTITADTSTSTAYMELSSLTSEDTAVYYC

B.199 GGTF....SNYAISWVRQAPGQGLEWMGGIIPI..FGTANYAQKFQ.GRATITADTSTSTAYMELSSLRSEDTAVYYC

B.2759 GGTF....SNYAISWVRQAPGQGLEWMGGIIPI..FGTANYAQKFQ.GRATITADTSTSTAYMELSSLRSEDTAVYYC

B.202 GGTF....SSYAISWVRQAPGQGPEWMGGIIPI..FGTANYAQKFQ.GRVTITADTSTSTAYMELSSLTSEDTAVYYC

B.221 GGTF....STYAISWVRQAPGQGLEWMGGIIPI..FGTANYAQKFQ.GRVTITADKSTSTVYMELSGLRSEDTAVYYC

B.225 GGTF....SSYAISWVRQAPGQGLEWMGGIIPI..FGTANYAQKFQ.GRVTITADRSTSTAYMELNSLTSEDTAVYYC

B.317 GGTF....SSYAISWVRQAPGQGLEWMGGIIPI..FGTANYAQKFQ.GRVTITADRSTSTAYMELNSLTSEDTAVYYC

B.226 GGTF....STYAISWVRQAPGQGLEWMGGIIPI..FGTANYAQKFQ.GRVTITADKSTSTAYMELSSLTSEDTALYYC

B.228 GGTF....SNYAISWVRQAPGQGLEWMGGIMPI..FGTANYAQKFQ.GRVTITADTSTSTAYMELSSLRSEDTAVYYC

B.231 GGTF....SNYAISWVRQAPGQGLEWMGGIIPI..FGTANYAQEFQ.GRVTITADKSTSTAYMELSSLRSEDTAIYYC

B.235 GGTF....SDYAISWVRQAPGQGLEWMGGIIPM..FGTANYAQKFQ.GRVTITADKSTSTTYMELSSLRSEDTAVYYC

B.238 GGTF....STYAISWVRQAPGQGLEWMGGIIPI..FGTANYAQKFQ.DRVTITADTSTSTAYMEVSSLTSEDTAVYYC

B.242 GGTF....SVYSISWVRQAPGQGLEWMGGIIPM..FGTANYAQKFQ.DRVTITADKSTSTAYMELTSLRSEDTAVYYC

B.251 GGTF....STYAICWVRQAPGQGLEWMGGIIPI..FGTANYAQKFQ.GRVTITADTSTSTAYMELSSLRSEDTAVYYC

B.256 GGTF....DNYAISWVRQAPGQGLEWMGGIIPI..FGTANYAQKFQ.GRVTITADKFTSTAYMELSSLRSEDTAVYYC

B.263 GGTF....DNYAISWVRQAPGQGLEWMGGIIPI..FGTANYAQKFQ.GRVTITADKFTSTAYMELSSLRSEDTAVYYC

B.258 GGTF....SSYAISWVRQAPGQGLEWMGGIIPI..FGTANYAQKFQ.GRVTITADTSTSTAYMEVSSLTSEDTAVYYC

B.304 GGTF....SSYAISWVRQAPGQGLEWMGGIIPI..FGTANYAQKFQ.GRVTITADTSTSTAYMEVSSLTSEDTAVYYC

B.282 GGTF....STYAISWVRQAPGQGLEWMGGIIPI..FGTANYAQKFQ.DRVTITADTSTSTGYMELSSLTSEDTAVYYC

B.283 GYTF....TSYGISWVRQAPGQGLEWMGGIIPI..FGTANYAQKFQ.GRVTITADKSTSTAYMELSSLRSEDTAVYYC

B.1060 GYTF....TSYGISWVRQAPGQGLEWMGGIIPI..FGTANYAQKFQ.GRVTITADKSTSTAYMELSSLRSEDTAVYYC

B.2907 GYTF....TSYGISWVRQAPGQGLEWMGGIIPI..FGTANYAQKFQ.GRVTITADKSTSTAYMELSSLRSEDTAVYYC

B.3581 GYTF....TSYGISWVRQAPGQGLEWMGGIIPI..FGTANYAQKFQ.GRVTITADKSTSTAYMELSSLRSEDTAVYYC

B.284 GGTF....STYAISWVRQAPGQGLEWMGGIIPI..FGTANYAQKFQ.GRVTITADTSTSTAYMELSSLRSEDAAVYYC

B.286 GGTF....STYAISWVRQAPGQGLEWMGGIIPI..FGTANYAQNFQ.GRVTITADKSTSTAYMELSSLRSEDTAIYYC

B.298 GGTF....STYAISWVRQAPGQGLEWMGGIIPI..FGTANYAQNFQ.GRVTITADKSTSTAYMELSSLRSEDTAIYYC

B.288 GGTF....STYAISWVRQAPGQGLEWMGGIIPI..FGTANYGQKFQ.GRVTITADKSTSTAYMELSSLRSDDTAVYYC

B.303 GGTF....SSYAISWVRQAPGQGLEWMGGIIPI..FGTANYAQKFQ.GRVTITADESTSTVYMELSSLKSEDTAVYYC

B.310 GGTF....STYAISWVRQAPGQGLEWMGGIIPI..FGTPNYAQKFQ.GRVTITADKSTSTAYMELSSLKSEDTAVYYC

B.312 GGTF....SSYAISWVRQAPGQGLEWMGGIIPI..FGTANYAERFQ.GRVTITADKSTNTAYMELSSLRSEDTAVYYC

B.340 GGTF....STYAISWVRQAPGQGLEWVGGIIPI..FGTANYAQKFQ.GRVTITADKSTSTAYMELSSLTSEDTAVYYC

B.346 GGTF....SSYAISWVRQVPGQGLEWMGGIIPI..FGTANYAQKFQ.GRVTITADKSTSTAYMEVSSLRSEDTAIYYC

B.348 GRTF....STYAISWVRQAPGQGLEWMGGIIPI..FGTANYAQKFQ.GRVTITADTSTSTAYMELSSLRSEDTAVYYC

B.358 GGIF....SNYAISWVRQAPGQGLEWMGGIIPI..FGTANYAQRFQ.GRVTITADKSTSTAYMELSSLRSEDTAVYYC

B.361 GGTF....STYAISWVRQAPGQGLEWMGGIIPI..FGTANYAQKFQ.GRVTITADRSTSTAYMELSSLRSEDTAVYFC

B.364 GGTF....SSYAISWVRQAPGQGLEWMGGIIPI..FGTANYAPKFQ.GRVTITADTSTSTAYMELNSLRSEDTAVYYC

B.2811 GGTF....SSYAISWVRQAPGQGLEWMGGIIPI..FGTANYAPKFQ.GRVTITADTSTSTAYMELNSLRSEDTAVYYC

B.369 GGTF....SSYAISWVRQAPGQGLEWMGGIIPI..LGIANYAQKFQ.GRVTITADKSTSTAYMELSSLRYEDTAVYYC

B.374 GYTF....SSYAISWVRQAPGQGLEWMGGIIPI..LGIANYAQKFQ.GRVTITADKSTSTAYMELSSLRSEDTAVYYC

B.384 GGTF....SSYAISWVRQAPGQGLEWMGGIIPM..LGIANYAQKFQ.GRVTITADKSTSTAYMELSSLRSEDTAVYYC

B.452 GGTF....ISYTITWVRQAPGQGLEWMGGIIPI..FGTANYAQKFQ.GRVTITADKSTSTAYMELSSLRSEDTAVYYC

B.456 GGTF....NTYAISWVRQAPGQGLEWMGGIIPI..FGTANYAQKFQ.GRVTITADESTSTAYMELSSLRSEDTAVYYC

B.466 GYTF....TSYAISWVRQAPGQGLEWMGGIIPI..FGTANYAQKFQ.GRVTITADESTSTAYMELSSLRSEDTAVYYC

B.606 GYTF....TSYAISWVRQAPGQGLEWMGGIIPI..FGTANYAQKFQ.GRVTITADESTSTAYMELSSLRSEDTAVYYC

B.1277 GYTF....TSYAISWVRQAPGQGLEWMGGIIPI..FGTANYAQKFQ.GRVTITADESTSTAYMELSSLRSEDTAVYYC

B.2400 GYTF....TSYAISWVRQAPGQGLEWMGGIIPI..FGTANYAQKFQ.GRVTITADESTSTAYMELSSLRSEDTAVYYC

B.3590 GYTF....TSYAISWVRQAPGQGLEWMGGIIPI..FGTANYAQKFQ.GRVTITADESTSTAYMELSSLRSEDTAVYYC

B.480 GYTF....SSYGITWVRQAPGQGLEWMGGIIPI..FGTANYAQKFQ.GRVTITADKSTSTAYMELSSLRSEDTAVYYC

B.509 GGTF....SSYAIIWVRQAPGQGLEWMGGIIPI..FGTANYAQKFQ.GRVTIIADESTSTAYMELSSLRSEDTAVYYC

B.521 GGTF....SSYAISWVRQVPGQGPEWMGGIIPI..FGTANYAQKFQ.GRVTITADESTSTAYMELSSLRSEDTAVYYC

B.532 GVTF....SNYAISWVRQAPGQGLEWMGGIIPI..FGTANYAQKFQ.GRVTITADESTSTAYMELSSLRSEDTAVYYC

B.538 GGTF....SNYAISWVRQAPGQGLEWMGGIIPI..FGTANYAQKFQ.GRVTITADESTSTAYMALSSLRSEDTAVYYC

B.540 GGTF....SSYAISWVRQAPGQGLEWMGGIIPI..FGTANYAQKFQ.GRVTITADQSTNTAYMEVSSLRSEDTAVYYC

B.541 GGTF....STYSISWVRQAPGQGLEWMGGIIPI..FGTANYAQKFQ.GRVTITADESTSTAYMELSSLRSEDTAVYYC

B.608 GGTF....STYAISWVRQASGQGLEWMGGIIPI..FGTANYAQKFQ.GRVTITADESTSTAYMELSSLRSEDTAVYYC

B.672 GGTF....SSDAISWVRQAPGQGLEWMGGIIPI..FGTANYAQKFQ.GRVTITADESTSTAYMELSSLRSENTAVYYC

B.705 GGTF....STYAISWVRQAPGQGLEWMGGIIPI..FGTANYAPKFQ.GRVTITADISTSTAYMELSSLRSEDTAVYYC

B.716 GGTF....SSYAISWVRQAPGQGLEWMGGIIPF..FGTANYAQKFQ.GRVTITADRSTSTAYMELSSLRSEDTAVYFC

B.750 RGTF....SDYAISWVRQAPGQGLEWMGGIIPI..FGTANYAQKFQ.GRVTITADESTSTAYMELSSLRSEDTAVYYC

B.875 GGTF....SSYAISWVRQAPGQGLEWMGGIIPM..FGTANYAQKFQ.GRVTITADESTSTAYMELSSLRSEDTAMYYC

B.887 GGTF....SSYAISWVRQAPGQGLEWMGGIIPI..FGTTKRAQKFQ.GRVTITADKSTSTAYMELSSLRSEDTAVYYC

B.916 GGTF....SKYTISWVRQAPGQGLEWMGGIIPI..FGTANYAQKFQ.GRVTITADESTSTAYMELSSLRSEDTAVYYC

B.946 GGTF....SSYAINWVRQAPGQGLEWMGGIIPL..FGTANYAQKFQ.GRVTITADESTSTAYMELSSLRSEDTAVYYC

B.958 GGTF....SSYAINWVRQAPGQGLEWMGGIIPI..FGTTNYAQKFQ.GRVTITADKSTGTAYMELSSLRSEDTAVYYC

B.972 GGTF....STYAISWVRQAPGQGLEWMGGIIPI..FGTENYAQKFQ.GRVTITADESTSTAYMELSSLRSEDTAVYYC

B.1037 GGTF....SSYAISWVRQAPGQGLEWMGGIIPI..FGTANYAQKFQ.GRVTITADKSTSTAYMELTSLSSQDTAVYYC

B.1085 GGTF....SNYAISWVRQAPGQGLEWMGGIIPI..FGTANYAQKFQ.GRVTITADESTRTAYMELSSLRSEDTAVYYC

B.1106 RGTF....SSYAISWVRQAPGQGLEWMGGIIPI..FGTANYAQKFQ.GRVTIIADESTSTAYMELSSLRSEDTAVYYC

B.1118 GGTF....STYAINWVRQAPGQGLEWMGGIIPV..FGTANYAQKFQ.GRVTITADKSTSTAYMELSSLRSEDTAVYYC

B.1126 GGTF....SSYAISWVRQAPGQGLEWMGGIIPI..FGSTNYAQKFQ.GRVTITADESTSTAYMELSSLRSEDTAVYYC

B.1138 GGTF....SRYAISWVRQAPGQGLEWMGGIIPI..FGTANHAQKFQ.GRVTITADESTSTAYMELSSLRSEDTAVYYC

B.1167 GGTF....SNYAISWVRQAPGQGLEWMGGIIPI..FGTANYAQKFQ.GRVTITADESTSTTYMELSSLRSEDTAVYYC

B.1168 GGTF....NSYAINWVRQAPGQGLEWMGGIIPI..FGTANYAQKFQ.GRVTITADESTSTAYMELSSLRSEDTAVYYC

B.1192 GGTF....SNYAISWVRQAPGQGLEWVGGIIPI..FGTANYAQKFQ.GRVTITADESTSTAYMELSSLRSEDTAVYYC

B.1298 GGTF....SSHAISWVRQAPGQGLEWMGGIIPI..FGTANYAQNFQ.GRVTITADESTSTAYMELSSLRSEDTAVYYC

B.1370 GGTF....SSYAISWVRQAPGQGLEWMGGIIPI..FGTANYAQKFQ.GRVTITEDTSTDTAYMELSSLRSEDTAVYYC

B.1404 GGSF....SMYPISWVRQAPGQGLEWMGGIIPI..FGTANYAQKFQ.GRVTITADKSTSTAYMELSSLRSEDTAVYYC

B.1493 GGTF....SSYAITWVRQAPGQGLEWMGGIIPI..FGTTNYAQKFQ.GRVTITADESTSTAYMELSSLRSEDTAVYYC

B.1511 GGTF....SSYASSWVRQAPGQGIEWMGGIIPI..FGTANYAQKFQ.GRVTITADESTSTAYMELSSLRSEDTAVYYC

B.1564 GGTF....SSYAISWVRQAPGQGLEWMGGIIPI..FGTANYGQKFQ.GRVTITADKSTSTAYMELNNLRSEDTAVYYC

B.1569 GGTF....NNYAISWVRQGPGQGLEWMGGTIPI..FGTANYAQKFQ.DRVTITADKSTSTAYMELSSLRSEDTAVYYC

B.1594 GGTF....SSYAISWVRQAPGQGLEWMGGIIPI..FGTAKYAQKFQ.GRVTITADKSTSTAYMELSSLIYEDTAVYYC

B.1626 GGTF....STYAISWVRQAPGQGLEWMGGIIPL..FGTANYAQKFQ.GRVTITADESTSTAYMELSSLRSEDTAVYYC

B.1645 GGTF....STYAISWVRQAPGQGLEWMGGIIPL..FGTANYAQKFQ.GRVTITADESTSTAYMELSSLRSEDTAVYYC

B.1632 GGTF....SSYAISWVRQAPGQGLEWVGGIIPI..FGTANYAQKFQ.GRVTITADESTSTAYMELSSLRYEDTAVYYC

B.1633 GGTF....SSSAISWVRQAPGQGLEWMGGIIPL..FGTANYAQKFQ.GRVTITADESTSTAYMELSSLRSEDTAVYYC

B.1663 GGTF....SSSAISWVRQAPGQGLEWMGGIIPL..FGTANYAQKFQ.GRVTITADESTSTAYMELSSLRSEDTAVYYC

B.1669 GGTF....SSSAISWVRQAPGQGLEWMGGIIPL..FGTANYAQKFQ.GRVTITADESTSTAYMELSSLRSEDTAVYYC

B.1772 GGTF....SSSAISWVRQAPGQGLEWMGGIIPL..FGTANYAQKFQ.GRVTITADESTSTAYMELSSLRSEDTAVYYC

B.1816 GGTF....SSSAISWVRQAPGQGLEWMGGIIPL..FGTANYAQKFQ.GRVTITADESTSTAYMELSSLRSEDTAVYYC

B.1875 GGTF....SSSAISWVRQAPGQGLEWMGGIIPL..FGTANYAQKFQ.GRVTITADESTSTAYMELSSLRSEDTAVYYC

B.1896 GGTF....SSSAISWVRQAPGQGLEWMGGIIPL..FGTANYAQKFQ.GRVTITADESTSTAYMELSSLRSEDTAVYYC

B.2729 GGTF....SSSAISWVRQAPGQGLEWMGGIIPL..FGTANYAQKFQ.GRVTITADESTSTAYMELSSLRSEDTAVYYC

B.2732 GGTF....SSSAISWVRQAPGQGLEWMGGIIPL..FGTANYAQKFQ.GRVTITADESTSTAYMELSSLRSEDTAVYYC

B.1675 GGTF....SSYAISWVRQAPGQGLEWVGGIIPI..FGTANYAQKFQ.GRVTITADESTTTAYMELSSLRSEDTAVYYC

B.2730 GGTF....SSYAISWVRQAPGQGLEWVGGIIPI..FGTANYAQKFQ.GRVTITADESTTTAYMELSSLRSEDTAVYYC

B.1685 GGTF....SNYAISWVRQAPGQGLEWMGGIIPI..FGTANYAQKFQ.GRVTITAQESTSTAYMELSSLRSEDTAVYYC

B.1686 GGTF....SSYAISWVRQAPGQGLEWMGGIIPI..FGTANYAQKFQ.GRVTITADESTTTAYMEVSSLRSEDTAVYYC

B.3551 GGTF....SSYAISWVRQAPGQGLEWMGGIIPI..FGTANYAQKFQ.GRVTITADESTTTAYMEVSSLRSEDTAVYYC

B.1690 GGTF....SSSAISWVRQAPGQGLEWMGGIIPI..FGTANYAQKFQ.GRVTITADESTSTAYMELSSLRSEDMAVYYC

B.1711 GGTF....SSYAISWVRQAPGQGLEWMGGIIPI..FGTANYAQKFQ.GRVTITADESTSTTYMELNSLRSEDTAVYYC

B.1726 GGTF....SSYAISWVRQAPGQGLEWMGGITPV..FGTANYAQKFQ.GRVTITADESTSTAYMELSSLRSEDTAVYYC

B.1742 GGTF....SSYAISWVRQAPGQGLEWMGGIIPI..FGTANYAQKFQ.GRVTITADESTNTAYMELSSLRSDDTAVYYC

B.1769 GGTF....SSYAISWVRQAPGQGLEWMGGIIPI..FGTPNYAQKFR.GRVTITADESTSTAYMELSSLRSEDTAVYYC

B.1775 GGTF....SSYAISWVRQAPGQGLEWMGGIIPI..FGRANYAQKFQ.GRVTITADESTSTAYMELSSLRSEDTAIYYC

B.1785 GGTF....SSYAISWVRQAPGQGLEWVGGIIPV..FGTANYAQKFQ.GRVTITADESTSTAYMELSSLRSEDTAVYYC

B.1805 GGTF....SSYAISWVRQAPGQGLEWMGGIIPI..FGTANYAQKFQ.GRVTITADESTRTAYMELSSLRSEDTAIYYC

B.1807 GGTF....SSYAISWVRQAPGQGLEWMGGIIPL..FGTANYAQKFQ.GRVTISADDSTSTAYMELSSLRSEDTAVYYC

B.1847 GGTF....SSYAISWVRQAPGQGLEWVGGIIPL..FGTANYAQKFQ.GRVTITADESTSTAYMELSSLRSEDTAVYYC

B.1860 GGTF....SSYAISWVRQAPGQGLEWVGGIIPL..FGTANYAQKFQ.GRVTITADESTSTAYMELSSLRSEDTAVYYC

B.1890 GGTF....SSYAISWVRQAPGQGLEWVGGIIPL..FGTANYAQKFQ.GRVTITADESTSTAYMELSSLRSEDTAVYYC

B.2743 GGTF....SSYAISWVRQAPGQGLEWVGGIIPL..FGTANYAQKFQ.GRVTITADESTSTAYMELSSLRSEDTAVYYC

B.1854 GGTF....SSYAISWVRQAPGQGLEWMGGIIPI..FGTANYAQKFQ.GRVTITADESTSTAYVELSSLRSEDTAVFYC

B.1857 GGTF....SSYSISWVRQAPGQGLEWMGGIIPI..FGTAKYAQKFQ.GRVTITADESTSTAYMELSSLRSEDTAVYYC

B.1874 GGTF....SSYAISLVRQAPGQGLEWMGGIIPI..FGTANYAQKFQ.GRVTITADESTSTTYMELSSLRSEDTAVYYC

B.1877 GGTF....SSSAISWVRQAPGQGLVWMGGIIPI..FGTANYAQKFQ.GRVTITADESTSTAYMELSSLRSEDTAVYYC

B.1947 GGTF....SNYAINWVRQAPGQGLEWMGGIIPI..FGTANYAQKFQ.GRVTITADVSTSTAYMELSSLRSEDTAVYYC

B.1948 GGTF....SSYAINWVRQAPGQGLEWMGGIIPI..FGTANYAQKFQ.GRVTITADESTSTAYMELNSLRSEDTAVYYC

B.1965 GGTF....SSYAINWVRQAPGQGLEWMGGIIPM..FGTANYAQKFQ.GRVTITADESTSTAYMELSSLRSEDTAVYYC

B.2029 GGTF....SSYAISWVRQAPGQGLEWMGGIIPL..FGTAHYAQKFQ.GRVTITADESTSTAYMELSSLRSEDTAVYYC

B.2052 GGTF....SRHPISWVRQAPGQGLEWMGGIIPI..FGTANYAQKFQ.GRVTITADKSTSTAYMELSSLRSEDTAVYYC

B.2066 GGTF....SNYAISWVRQAPGQSLEWMGGIIPI..FGTANYAQKFQ.GRVTITADESTSTAYMELSSLRSEDTAVYYC

B.2070 GGTF....SSYVISWVRQAPGQGLEWMGGIIPI..FGTANYAQKFQ.GRVTITADESTSTVYMELSSLRSEDTAVYYC

B.2086 GGTF....SNYAISWVRQAPGQGLEWMGGIIPI..FGTANYAQKFQ.GRVTISADESTSTAYMELSSLRSEDTAVYYC

B.2101 GGTF....SSYAISWVRQAPGQGLEWMGGIIPI..FGTANYAQKFQ.GRITITADESTSTAFMELSSLRSEDTAVYYC

B.2116 GYTF....SSYTISWVRQAPGQGLEWMGGIIPI..FGTANYAQKFQ.GRVTITADESTSTAYMELSSLRSEDTAVYYC

B.2128 GGTF....SSYAISWVRQAPGQGLEWMGRIIPI..LGLANYAQKFQ.GRVTITADKSTSTAYMELSSLRSEDTAVYYC

B.2168 GGTF....SSYAISWVRQAPGQGLEWMGGIIPI..FGTANYAQKFQ.GRVTITADESTSTAYMELTSLRSEDTALYYC

B.2277 GGTF....SSYAISWVRQAPGQGLEWMGGIIPI..FGTANYAQKFQ.GRVTITADESTRTAYMELSSLRFEDTAVYYC

B.2278 GGTF....SSYAIAWVRQAPGQGLEWMGGIIPI..FGTANYAQKFQ.GRVTITADESTSTAYMEMSSLRSEDTAVYYC

B.2332 GGTF....SSYAISWVRQAPGQGLEWMGGIIPF..FSTANYAQKFQ.GRVTITADKSTSTAYMELSSLRSEDTAVYFC

B.2337 GGTF....SSYAISWVRQAPGQGLEWMGGIIPI..FGTANYAQKFQ.GRVTITADESTSTAYMELSSLKSEDTAVYFC

B.2343 GGTF....SSYAISWVRQAPGQGLEWMGGIIPI..FGTANYAQKFQ.GRVTITADKSTSTAFMELTSLRSEDSAVYYC

B.2360 GGTF....SSYAISWVRQAPGQGLEWMGGIIPI..FGTAHYAQKFQ.GRVTINADESTSTAYMELSSLRSEDTAVYYC

B.2509 GGTF....SSYAISWLRQAPGQGLEWMGRIIPI..FGTANYAQKFQ.GRVTITADESTSTAYMELSSLRSEDTAVYYC

B.2613 GGTF....SSYAISWVRQAPGQGLEWMGGIIPI..FGIVNYAQKFQ.GRVTITADKSTSTAYMELSSLRSEDTALYYC

B.2620 GGTF....SSYAISWVRQAPGQGLEWMGGIIPI..FGTANYAHKFQ.GRVTITADESTSTVYMELSSLRSEDTAVYYC

B.2624 GGTF....SSYAISWVRQAPGQGLEWMGGIIPM..FDIANYAQKFQ.GRVTITADKSTSTAYMELSSLRSEDTAVYYC

B.2703 GGTF....SSYAISWVRQAPGQGLEWVGGIIPI..FGTANYAQKFQ.GRVTITADESTSTAYMELSSLRSDDTAVYYC

B.2727 GGTF....SSYAISWVRQAPGQGLEWVGGIIPI..FGTANYAQKFQ.GRVTITADESTSTAYMELSSLRSDDTAVYYC

B.2716 GGTF....SSSAISWVRQAPGQGLEWMGGIIPI..FGTANYAQKFQ.GRVTITADESTTTAYMELSSLRSEDTAVYYC

B.2751 GGTF....STYAISWVRQAPGQGLEWMGGIIPI..FGTANYAQKFQ.GRVTITADKSTSTVYMELSSLTSEDTAVYYC

B.2752 GGTF....SNYAISWVRQAPGQGLEWMGGIIPI..FGTANYAQKFQ.GRVTITADKSTSTAYMELTSLTSEDTAVYYC

B.2755 GGTF....SSYAISWVRQAPGQGLEWMGGIIPI..FGTANYAQKFQ.GRVTITADTSTSTAYMELNSLTSEDTAVYYC

B.2758 GGTF....SNYAISWVRQAPGQGLEWMGGIIPI..FGTANYAQKFQ.GRVTITADTSTSTAYMELSSLTSEDTAVYYC

B.2836 GGTF....SNYAISWVRQAPGQGLEWMGGIIPI..FGTANYAQKFQ.GRVTITADTSTSTAYMELSSLTSEDTAVYYC

B.2771 GGTF....STYAISWVRQAPGQGLEWMGGIIPI..FGTANYAQKFQ.GRVTITADTSTSTAYMELSSLRSEDTAMYYC

B.2774 GGTF....SSYAISWVRQAPGQGLEWMGGIIPI..FGTANYAQKFQ.GRVTITADTSTNTAYMELSSLTSEDTAVYYC

B.2779 GGTF....SSYAISWVRQAPGQGLEWMGGIIPI..FGTANYAQKFQ.GRATITADKSTTTAYMELSSLTSEDTAVYYC

B.2786 GGTF....STYAISWVRQAPGQGLEWMGGIIPL..FGTANYAQKFQ.GRVTITADKSTSTAYMELSGLRSEDTAVYYC

B.2832 GGTF....STYAISWVRQAPGQGLEWMGGIIPI..FGTANYAQKFQ.GRVTITADTSTSTAYMELSSLRSEDTALYYC

B.2835 GGTF....STYAISWVRQAPGHGLEWMGGIIPI..FGTANYAQKFQ.GRVTVTADKSTSTAYMELSSLRSEDTAVYYC

B.2841 GGTF....STYAISWVRQAPGQGLEWMGGIIPI..FGTANYAQKFQ.GRVTITADTSTNTAYMELSSLRSEDTAVYYC

B.2849 GGTF....STYAISWVRQAPGQGLEWMGGIIPI..FGTANYAQKFQ.GRATITADKSTSTAYMELSSLTSEDTAVYYC

B.2850 GGTF....SSYAISWVRQAPGQGLEWMGGINPI..FGTPNYAQKFQ.GRVTITADKSTSTAYMELTSLRSEDTAVYYC

B.2855 GGTF....SSYAISWVRQAPGQGLEWMGGIIPI..FGTANYAQKFQ.GRVTITADTSTSTAYMELSSLSSEDTAKYYC

B.2859 GGTF....SNYAISWVRQAPGQGLEWMGGIIPI..FGTTNYAQKFQ.GRVTITADESTSTAYMELSSLRSEDTAVYYC

B.2863 GGTF....SSYAISWVRQAPGQGLEWMGGIIPI..FGTANYAQKFQ.GRVTITADTSTSIVYMELSSLRSEDTAVYYC

B.2912 GGTF....SSYAISWVRQAPGQGLEWMGGIIPI..FGTANYAQKFQ.GRVTITADESTSTAYMELSSLRSDDTALYYC

B.2913 GGTF....SSYAISWVRQAPGQGLEWMGGIIPI..YGTPNYAQKFQ.GRVTITADESTSTAYMELSSLRSEDTAVYYC

B.2917 GGTF....SSYGISWVRQAPGQGLEWMGRIIPI..FGTINYAQKFQ.GRVTITADKSTSTAYMELSSLRSEDTAVYYC

B.2995 GGTF....SSYAISWVRQAPGQRLEWMGGIIPI..FGTANYAQKFL.GRVTITADESTSTAYMELSSLRSEDTAVYYC

B.3010 GYTF....ISYAISWVRQAPGQGLEWMGGIIPI..FGTANYAQKFQ.GRVTITADESTSTAYMELSSLRSEDTAVYYC

B.3113 GGTF....SNYAISWVRQAPGQGLEWMGGIIPI..FGATNYAQKFQ.GRVTITADKSTSTAYMELSSLRSEDTAVYYC

B.3176 GGTF....SSYAISWVRQAPGQGLEWMGGIIPI..FGTANYAQKFQ.GRITITADNSTSTAYMEVSSLRSEDTAVYYC

B.3183 GGTF....SSYAISWVRQAPGQGLEWMGGIIPI..FATANYAQKFQ.GRVTITADDSTSTAYMELSSLRSQDTAVYYC

B.3262 GGTF....SSFAISWVRQAPGQGLEWMGGIIPI..FGSANYAQKFQ.GRVTITADKSTTTAYMELSSLRSEDTAVYYC

B.3332 GGTF....SSYAISWVRQAPGQGLEWMGWIIPI..LGIANYAQKFQ.GRVTITADKSTSTAYMELSSLRSEDTAVYYC

B.3416 GGTF....STYAISWVRQAPGQGLEWMGGIIPI..LGIANYAQKFQ.GRVTITADKSTSTAYMELSSLRSEDTAVYYC

B.3461 GGTF....NSFAISWVRQAPGQGLEWMGGIIPI..FGTANYAQKFQ.GRVTITADESTSTAYMELSSLRSEDTAVYYC

B.3487 GGTF....SSYAISWVRQAPGQGLEWMGGIIPI..FGIANYAQKFQ.GRVTITADESTSTAYMELSSLRFEDTAVYYC

B.3493 GGTF....SSYAISWVRQAPGQGPEWMGGIIPI..FGTANYAQQFQ.CRVTITADESTSTAYMELSSLRSEDTAVYYC

B.3511 GGTF....SSFAISWVRQAPGQGLEWMGGIIPI..FGTANYAQKFQ.GRVTITADRSTSTAFMELSSLRSEDTAVYYC

B.3534 GGTF....SSYAISWVRQAPGQGLEWMGGIIPI..FGTANYAHKFQ.GRVTITADTSTNTAYMELSSLRSEDTAVYYC

B.3535 GGTF....SSYAISWVRQAPGQGLEWMGGIIPI..FGTANYVQKFQ.GRVTITADKSTNTVYMELSSLRSEDTAVYYC

B.3571 GGTF....SSYAISWVRQAPGQGLEWMGGIIPI..FGTANYAQKFQ.GRVTITADESTSTAYMELRSLSSEDTAVYYC

B.3604 GGTF....SSYAISWVRQAPGQGLEWMGGIIPI..FGTANYAQKFQ.GRVTITADKSTSTAYMELTSLISEDTAIYYC

B.3622 GGTF....SSYAISWVRQAPGQGLEWMGGIIPI..FGTANYAQKFQ.GRVTITADESTSTAYLELSSLRSEDTAMYYC

B.3626 GGTF....SNYAISWVRQAPGQGLEWMGGIIPI..FGTANYAQKFQ.GRVTITADESTSTAYMELNSLRSEDTAVYYC

**E. Alignment with six exchanges________________________________________**

Weber et. al Sequence 1334_05_H04,1198_01_E11 and 1198_03_G07

GGTF....SSYAISWVRQAPGQGLEWMGGIIPI..FGTANYAQKLQ.DRVTITADKSTSTAYMELSSLRSEDTAVYYC

GGTF....SFQTISWVRQAPGQGLEWMGGIIPI..FGTPSYEQKFQ.GRVTITADKSTSTAYMELSSLRSEDTAVYYC

Aligned Sequences (n=316) from our cohort

B.14 GGTF....SSYAISWVRQAPGQGLEWMGRIIPI..LGIANYAQKFK.GRVTITADKSTSTAYMELSSLRSEDTAVYYC

B.39 GGTF....SSYAFSWVRQAPGQGLEWMGGITPI..FGTPNYAQKFQ.GRVTITADKSTTTAYMELSSLRSEDTAVYYC

B.61 GGTF....ASYAISWVRQAPGQGLEWMGRIIPI..LGTANYAQKFQ.GRVTITADESTSTAYMELSSLRSEDTAVYYC

B.63 GGSF....STYPISWVRQAPGQGLEWMGGIIPI..FGTANYAQKFQ.GRVTITADESTSTAYMELSSLRSEDTAVYYC

B.86 GGTF....RSYAFSWVRQAPGQGLEWMGGIIPI..FDTTNYAQKFQ.GRVTITADKSTSTAYMELSSLRSEDTAVYYC

B.110 GGTF....SSYTISWVRQAPGQGLEWMGRIIPI..LGIANYAQKFQ.GRVTITADKSTSTAYMELSSLRSEDTAVYYC

B.117 GGTF....SSYTISWVRQAPGQGLEWMGRIIPI..LGIANYAQKFQ.GRVTITADKSTSTAYMELSSLRSEDTAVYYC

B.118 GGTF....SSYTISWVRQAPGQGLEWMGRIIPI..LGIANYAQKFQ.GRVTITADKSTSTAYMELSSLRSEDTAVYYC

B.119 GGTF....SSYTISWVRQAPGQGLEWMGRIIPI..LGIANYAQKFQ.GRVTITADKSTSTAYMELSSLRSEDTAVYYC

B.127 GGTF....SSYTISWVRQAPGQGLEWMGRIIPI..LGIANYAQKFQ.GRVTITADKSTSTAYMELSSLRSEDTAVYYC

B.128 GGTF....SSYTISWVRQAPGQGLEWMGRIIPI..LGIANYAQKFQ.GRVTITADKSTSTAYMELSSLRSEDTAVYYC

B.142 GGTF....SSYTISWVRQAPGQGLEWMGRIIPI..LGIANYAQKFQ.GRVTITADKSTSTAYMELSSLRSEDTAVYYC

B.159 GGTF....SSYTISWVRQAPGQGLEWMGRIIPI..LGIANYAQKFQ.GRVTITADKSTSTAYMELSSLRSEDTAVYYC

B.166 GGTF....SSYTISWVRQAPGQGLEWMGRIIPI..LGIANYAQKFQ.GRVTITADKSTSTAYMELSSLRSEDTAVYYC

B.176 GGTF....SSYTISWVRQAPGQGLEWMGRIIPI..LGIANYAQKFQ.GRVTITADKSTSTAYMELSSLRSEDTAVYYC

B.182 GGTF....SSYTISWVRQAPGQGLEWMGRIIPI..LGIANYAQKFQ.GRVTITADKSTSTAYMELSSLRSEDTAVYYC

B.183 GGTF....SSYTISWVRQAPGQGLEWMGRIIPI..LGIANYAQKFQ.GRVTITADKSTSTAYMELSSLRSEDTAVYYC

B.185 GGTF....SSYTISWVRQAPGQGLEWMGRIIPI..LGIANYAQKFQ.GRVTITADKSTSTAYMELSSLRSEDTAVYYC

B.189 GGTF....SSYTISWVRQAPGQGLEWMGRIIPI..LGIANYAQKFQ.GRVTITADKSTSTAYMELSSLRSEDTAVYYC

B.398 GGTF....SSYTISWVRQAPGQGLEWMGRIIPI..LGIANYAQKFQ.GRVTITADKSTSTAYMELSSLRSEDTAVYYC

B.414 GGTF....SSYTISWVRQAPGQGLEWMGRIIPI..LGIANYAQKFQ.GRVTITADKSTSTAYMELSSLRSEDTAVYYC

B.418 GGTF....SSYTISWVRQAPGQGLEWMGRIIPI..LGIANYAQKFQ.GRVTITADKSTSTAYMELSSLRSEDTAVYYC

B.419 GGTF....SSYTISWVRQAPGQGLEWMGRIIPI..LGIANYAQKFQ.GRVTITADKSTSTAYMELSSLRSEDTAVYYC

B.498 GGTF....SSYTISWVRQAPGQGLEWMGRIIPI..LGIANYAQKFQ.GRVTITADKSTSTAYMELSSLRSEDTAVYYC

B.502 GGTF....SSYTISWVRQAPGQGLEWMGRIIPI..LGIANYAQKFQ.GRVTITADKSTSTAYMELSSLRSEDTAVYYC

B.519 GGTF....SSYTISWVRQAPGQGLEWMGRIIPI..LGIANYAQKFQ.GRVTITADKSTSTAYMELSSLRSEDTAVYYC

B.520 GGTF....SSYTISWVRQAPGQGLEWMGRIIPI..LGIANYAQKFQ.GRVTITADKSTSTAYMELSSLRSEDTAVYYC

B.528 GGTF....SSYTISWVRQAPGQGLEWMGRIIPI..LGIANYAQKFQ.GRVTITADKSTSTAYMELSSLRSEDTAVYYC

B.530 GGTF....SSYTISWVRQAPGQGLEWMGRIIPI..LGIANYAQKFQ.GRVTITADKSTSTAYMELSSLRSEDTAVYYC

B.546 GGTF....SSYTISWVRQAPGQGLEWMGRIIPI..LGIANYAQKFQ.GRVTITADKSTSTAYMELSSLRSEDTAVYYC

B.551 GGTF....SSYTISWVRQAPGQGLEWMGRIIPI..LGIANYAQKFQ.GRVTITADKSTSTAYMELSSLRSEDTAVYYC

B.593 GGTF....SSYTISWVRQAPGQGLEWMGRIIPI..LGIANYAQKFQ.GRVTITADKSTSTAYMELSSLRSEDTAVYYC

B.610 GGTF....SSYTISWVRQAPGQGLEWMGRIIPI..LGIANYAQKFQ.GRVTITADKSTSTAYMELSSLRSEDTAVYYC

B.612 GGTF....SSYTISWVRQAPGQGLEWMGRIIPI..LGIANYAQKFQ.GRVTITADKSTSTAYMELSSLRSEDTAVYYC

B.614 GGTF....SSYTISWVRQAPGQGLEWMGRIIPI..LGIANYAQKFQ.GRVTITADKSTSTAYMELSSLRSEDTAVYYC

B.641 GGTF....SSYTISWVRQAPGQGLEWMGRIIPI..LGIANYAQKFQ.GRVTITADKSTSTAYMELSSLRSEDTAVYYC

B.703 GGTF....SSYTISWVRQAPGQGLEWMGRIIPI..LGIANYAQKFQ.GRVTITADKSTSTAYMELSSLRSEDTAVYYC

B.714 GGTF....SSYTISWVRQAPGQGLEWMGRIIPI..LGIANYAQKFQ.GRVTITADKSTSTAYMELSSLRSEDTAVYYC

B.732 GGTF....SSYTISWVRQAPGQGLEWMGRIIPI..LGIANYAQKFQ.GRVTITADKSTSTAYMELSSLRSEDTAVYYC

B.753 GGTF....SSYTISWVRQAPGQGLEWMGRIIPI..LGIANYAQKFQ.GRVTITADKSTSTAYMELSSLRSEDTAVYYC

B.768 GGTF....SSYTISWVRQAPGQGLEWMGRIIPI..LGIANYAQKFQ.GRVTITADKSTSTAYMELSSLRSEDTAVYYC

B.771 GGTF....SSYTISWVRQAPGQGLEWMGRIIPI..LGIANYAQKFQ.GRVTITADKSTSTAYMELSSLRSEDTAVYYC

B.774 GGTF....SSYTISWVRQAPGQGLEWMGRIIPI..LGIANYAQKFQ.GRVTITADKSTSTAYMELSSLRSEDTAVYYC

B.799 GGTF....SSYTISWVRQAPGQGLEWMGRIIPI..LGIANYAQKFQ.GRVTITADKSTSTAYMELSSLRSEDTAVYYC

B.800 GGTF....SSYTISWVRQAPGQGLEWMGRIIPI..LGIANYAQKFQ.GRVTITADKSTSTAYMELSSLRSEDTAVYYC

B.813 GGTF....SSYTISWVRQAPGQGLEWMGRIIPI..LGIANYAQKFQ.GRVTITADKSTSTAYMELSSLRSEDTAVYYC

B.816 GGTF....SSYTISWVRQAPGQGLEWMGRIIPI..LGIANYAQKFQ.GRVTITADKSTSTAYMELSSLRSEDTAVYYC

B.827 GGTF....SSYTISWVRQAPGQGLEWMGRIIPI..LGIANYAQKFQ.GRVTITADKSTSTAYMELSSLRSEDTAVYYC

B.837 GGTF....SSYTISWVRQAPGQGLEWMGRIIPI..LGIANYAQKFQ.GRVTITADKSTSTAYMELSSLRSEDTAVYYC

B.842 GGTF....SSYTISWVRQAPGQGLEWMGRIIPI..LGIANYAQKFQ.GRVTITADKSTSTAYMELSSLRSEDTAVYYC

B.1201 GGTF....SSYTISWVRQAPGQGLEWMGRIIPI..LGIANYAQKFQ.GRVTITADKSTSTAYMELSSLRSEDTAVYYC

B.1219 GGTF....SSYTISWVRQAPGQGLEWMGRIIPI..LGIANYAQKFQ.GRVTITADKSTSTAYMELSSLRSEDTAVYYC

B.1220 GGTF....SSYTISWVRQAPGQGLEWMGRIIPI..LGIANYAQKFQ.GRVTITADKSTSTAYMELSSLRSEDTAVYYC

B.1228 GGTF....SSYTISWVRQAPGQGLEWMGRIIPI..LGIANYAQKFQ.GRVTITADKSTSTAYMELSSLRSEDTAVYYC

B.1233 GGTF....SSYTISWVRQAPGQGLEWMGRIIPI..LGIANYAQKFQ.GRVTITADKSTSTAYMELSSLRSEDTAVYYC

B.1252 GGTF....SSYTISWVRQAPGQGLEWMGRIIPI..LGIANYAQKFQ.GRVTITADKSTSTAYMELSSLRSEDTAVYYC

B.1264 GGTF....SSYTISWVRQAPGQGLEWMGRIIPI..LGIANYAQKFQ.GRVTITADKSTSTAYMELSSLRSEDTAVYYC

B.1271 GGTF....SSYTISWVRQAPGQGLEWMGRIIPI..LGIANYAQKFQ.GRVTITADKSTSTAYMELSSLRSEDTAVYYC

B.1279 GGTF....SSYTISWVRQAPGQGLEWMGRIIPI..LGIANYAQKFQ.GRVTITADKSTSTAYMELSSLRSEDTAVYYC

B.1292 GGTF....SSYTISWVRQAPGQGLEWMGRIIPI..LGIANYAQKFQ.GRVTITADKSTSTAYMELSSLRSEDTAVYYC

B.1297 GGTF....SSYTISWVRQAPGQGLEWMGRIIPI..LGIANYAQKFQ.GRVTITADKSTSTAYMELSSLRSEDTAVYYC

B.1336 GGTF....SSYTISWVRQAPGQGLEWMGRIIPI..LGIANYAQKFQ.GRVTITADKSTSTAYMELSSLRSEDTAVYYC

B.1378 GGTF....SSYTISWVRQAPGQGLEWMGRIIPI..LGIANYAQKFQ.GRVTITADKSTSTAYMELSSLRSEDTAVYYC

B.1401 GGTF....SSYTISWVRQAPGQGLEWMGRIIPI..LGIANYAQKFQ.GRVTITADKSTSTAYMELSSLRSEDTAVYYC

B.1702 GGTF....SSYTISWVRQAPGQGLEWMGRIIPI..LGIANYAQKFQ.GRVTITADKSTSTAYMELSSLRSEDTAVYYC

B.1749 GGTF....SSYTISWVRQAPGQGLEWMGRIIPI..LGIANYAQKFQ.GRVTITADKSTSTAYMELSSLRSEDTAVYYC

B.1812 GGTF....SSYTISWVRQAPGQGLEWMGRIIPI..LGIANYAQKFQ.GRVTITADKSTSTAYMELSSLRSEDTAVYYC

B.1823 GGTF....SSYTISWVRQAPGQGLEWMGRIIPI..LGIANYAQKFQ.GRVTITADKSTSTAYMELSSLRSEDTAVYYC

B.1841 GGTF....SSYTISWVRQAPGQGLEWMGRIIPI..LGIANYAQKFQ.GRVTITADKSTSTAYMELSSLRSEDTAVYYC

B.1903 GGTF....SSYTISWVRQAPGQGLEWMGRIIPI..LGIANYAQKFQ.GRVTITADKSTSTAYMELSSLRSEDTAVYYC

B.1912 GGTF....SSYTISWVRQAPGQGLEWMGRIIPI..LGIANYAQKFQ.GRVTITADKSTSTAYMELSSLRSEDTAVYYC

B.1916 GGTF....SSYTISWVRQAPGQGLEWMGRIIPI..LGIANYAQKFQ.GRVTITADKSTSTAYMELSSLRSEDTAVYYC

B.1938 GGTF....SSYTISWVRQAPGQGLEWMGRIIPI..LGIANYAQKFQ.GRVTITADKSTSTAYMELSSLRSEDTAVYYC

B.2127 GGTF....SSYTISWVRQAPGQGLEWMGRIIPI..LGIANYAQKFQ.GRVTITADKSTSTAYMELSSLRSEDTAVYYC

B.2411 GGTF....SSYTISWVRQAPGQGLEWMGRIIPI..LGIANYAQKFQ.GRVTITADKSTSTAYMELSSLRSEDTAVYYC

B.2416 GGTF....SSYTISWVRQAPGQGLEWMGRIIPI..LGIANYAQKFQ.GRVTITADKSTSTAYMELSSLRSEDTAVYYC

B.2424 GGTF....SSYTISWVRQAPGQGLEWMGRIIPI..LGIANYAQKFQ.GRVTITADKSTSTAYMELSSLRSEDTAVYYC

B.2431 GGTF....SSYTISWVRQAPGQGLEWMGRIIPI..LGIANYAQKFQ.GRVTITADKSTSTAYMELSSLRSEDTAVYYC

B.2442 GGTF....SSYTISWVRQAPGQGLEWMGRIIPI..LGIANYAQKFQ.GRVTITADKSTSTAYMELSSLRSEDTAVYYC

B.2447 GGTF....SSYTISWVRQAPGQGLEWMGRIIPI..LGIANYAQKFQ.GRVTITADKSTSTAYMELSSLRSEDTAVYYC

B.2450 GGTF....SSYTISWVRQAPGQGLEWMGRIIPI..LGIANYAQKFQ.GRVTITADKSTSTAYMELSSLRSEDTAVYYC

B.2454 GGTF....SSYTISWVRQAPGQGLEWMGRIIPI..LGIANYAQKFQ.GRVTITADKSTSTAYMELSSLRSEDTAVYYC

B.2466 GGTF....SSYTISWVRQAPGQGLEWMGRIIPI..LGIANYAQKFQ.GRVTITADKSTSTAYMELSSLRSEDTAVYYC

B.2488 GGTF....SSYTISWVRQAPGQGLEWMGRIIPI..LGIANYAQKFQ.GRVTITADKSTSTAYMELSSLRSEDTAVYYC

B.2490 GGTF....SSYTISWVRQAPGQGLEWMGRIIPI..LGIANYAQKFQ.GRVTITADKSTSTAYMELSSLRSEDTAVYYC

B.2492 GGTF....SSYTISWVRQAPGQGLEWMGRIIPI..LGIANYAQKFQ.GRVTITADKSTSTAYMELSSLRSEDTAVYYC

B.2702 GGTF....SSYTISWVRQAPGQGLEWMGRIIPI..LGIANYAQKFQ.GRVTITADKSTSTAYMELSSLRSEDTAVYYC

B.2721 GGTF....SSYTISWVRQAPGQGLEWMGRIIPI..LGIANYAQKFQ.GRVTITADKSTSTAYMELSSLRSEDTAVYYC

B.2960 GGTF....SSYTISWVRQAPGQGLEWMGRIIPI..LGIANYAQKFQ.GRVTITADKSTSTAYMELSSLRSEDTAVYYC

B.2962 GGTF....SSYTISWVRQAPGQGLEWMGRIIPI..LGIANYAQKFQ.GRVTITADKSTSTAYMELSSLRSEDTAVYYC

B.2964 GGTF....SSYTISWVRQAPGQGLEWMGRIIPI..LGIANYAQKFQ.GRVTITADKSTSTAYMELSSLRSEDTAVYYC

B.2974 GGTF....SSYTISWVRQAPGQGLEWMGRIIPI..LGIANYAQKFQ.GRVTITADKSTSTAYMELSSLRSEDTAVYYC

B.3387 GGTF....SSYTISWVRQAPGQGLEWMGRIIPI..LGIANYAQKFQ.GRVTITADKSTSTAYMELSSLRSEDTAVYYC

B.3393 GGTF....SSYTISWVRQAPGQGLEWMGRIIPI..LGIANYAQKFQ.GRVTITADKSTSTAYMELSSLRSEDTAVYYC

B.3395 GGTF....SSYTISWVRQAPGQGLEWMGRIIPI..LGIANYAQKFQ.GRVTITADKSTSTAYMELSSLRSEDTAVYYC

B.3396 GGTF....SSYTISWVRQAPGQGLEWMGRIIPI..LGIANYAQKFQ.GRVTITADKSTSTAYMELSSLRSEDTAVYYC

B.3405 GGTF....SSYTISWVRQAPGQGLEWMGRIIPI..LGIANYAQKFQ.GRVTITADKSTSTAYMELSSLRSEDTAVYYC

B.3415 GGTF....SSYTISWVRQAPGQGLEWMGRIIPI..LGIANYAQKFQ.GRVTITADKSTSTAYMELSSLRSEDTAVYYC

B.3417 GGTF....SSYTISWVRQAPGQGLEWMGRIIPI..LGIANYAQKFQ.GRVTITADKSTSTAYMELSSLRSEDTAVYYC

B.3418 GGTF....SSYTISWVRQAPGQGLEWMGRIIPI..LGIANYAQKFQ.GRVTITADKSTSTAYMELSSLRSEDTAVYYC

B.3423 GGTF....SSYTISWVRQAPGQGLEWMGRIIPI..LGIANYAQKFQ.GRVTITADKSTSTAYMELSSLRSEDTAVYYC

B.201 GGTF....STYAISWVRQGPGQGLEWMGGIIPI..FGTANYAQKFQ.GRVTITADTSTSTAYMELSSLTSEDTAVYYC

B.209 GGTF....STYAISWVRQAPGQGLEWMGGIIPI..FGTANYAQKFQ.GRVTITADTSTSTAYLELSSLTSEDTAVYYC

B.218 GGTF....STYAVSWVRQAPGQGLEWMGGIIPI..FGTANYAQKFQ.GRVTITADTSTSTAYMELSSLTSEDTAVYYC

B.222 GGTF....NSHAISWVRQAPGQGLEWMGGIIPI..FGTANYAQKFQ.GRVTITADTSTSTAYMELSSLTSEDTAVYYC

B.252 GGTF....SSNAISWVRQAPGQGLEWMGGIIPI..FGRANYAQKFQ.GRVTITADESTSTVYMELSSLRSEDTAVYYC

B.254 GYTF....TSYAISWVRQAPGQGLEWMGGIIPI..FGTANYAQKFQ.GRVTITADTSTSTAYMELSSLTSEDTAVYYC

B.267 GGTF....SSYAISWVRQAPGQGLEWMGGIIPI..FGTANYAQKFQ.GRVTITADESTSTAYMELNNLRSEDTAVYFC

B.269 GGTF....STYAISWVRQAPGQGLEWMGGIIPL..FGTANYAQKFQ.GRVTITADTSTSTAYMELSSLTSEDTAVYYC

B.280 GGTF....SNYAINWVRQAPGQGLEWMGGIIPI..FGTANYAQKFQ.GRVTITADTSTSTAYMELSSLTSEDTAVYYC

B.281 GGTF....GSHAISWVRQAPGQGLEWMGGIIPI..FGTANYAQKFQ.GRATITADTSTSTAYMELSSLRSEDTAVYYC

B.285 GGSF....SNYAISCVRQAPGQGLEWMGGIIPI..FGTANYAQKFQ.GRATITADKSTSTAYMELSSLRSEDTAVYYC

B.302 GGTF....SSYAISWVRQAPGQGLEWMGGIIPI..FGTANYAQKFQ.GRVTITADKSTTTVYMEVSRLRSEDTAVYYC

B.313 GGTF....SNYAISWVRQAPGQGLEWMGGIIPI..FGTANYAQKFQ.GRVTITADKSTSTAYMELNSLTSEDTAYYYC

B.315 GGTF....STYAISWVRQAPGQGLEWMGGIIPI..FGTANYAQKFQ.GRVTITADTSTSTAYMEVSSLTSEDTAVYYC

B.318 GGTF....STYAISWVRQAPGQGLEWMGGIIPI..FGTANYAQKFQ.GRVTITADISTSIVYMELSSLRSEDTAVYYC

B.321 GGTF....STYAISWVRQAPGQGLEWMGGIIPI..FGTANYAQKFQ.GRVTITADKSTSTDYMELSSLSSEDTAMYYC

B.322 GGTF....SSYAISWVRQAPGQGLEWMGGIIPI..FDTANYAQKFQ.GRVTITADESTSTAYMEVSSLRSEDMAVYYC

B.332 GGTF....STYAISWVRQAPGQGLEWMGGIIPI..FGTANYAQKFQ.GRVTITADKSTSTAYMDLSSLKSDDTAVYYC

B.333 GGTF....SNYAISWVRQAPGQGLEWMGGIIPI..FGTANYAQKFQ.GRVTITADKSTNTAYMELSSLISEDSAVYYC

B.338 GGTF....SSYAISWVRQAPGQGLEWMGGIIPI..FGTANYAQKFQ.GRVTITADRSTSTAYMELNSLRYDDTAVYYC

B.341 GGTF....STYAISWVRQAPGQGLEWMGGIIPI..FGTANYAHNFQ.GRVTITADKSTSTTYMELSSLRSEDTAVYYC

B.349 GGTF....SSYAISWVRQAPGQGLEWMGGIIPL..FGTANYAQKFQ.GRVTITADESTSTAYMELSNLRSEDTAVYFC

B.350 GGTF....SSYAISWVRQAPGQGLEWMGGIIPI..FGTANYAQKFQ.GRVTITADTSTSTAYMELNSLTSEDTAAYYC

B.412 GGTF....SSYAISWVRQAPGQGLEWMGGIIPI..LGIANYAQKFQ.GRVTITADKSTSTAYMELRSLRSDDTAVYYC

B.424 GGTV....SNYAINWVRQAPGQGLEWMGGIIPI..FGSANYAQKFQ.GRVTITADKSTSTAYMELSSLRSEDTAVYYC

B.450 GGTF....SSYAISWVRQAPGQGLEWMGGIIPM..FSTSNYAQKFQ.GRVRITADKSTSTAYMELSSLRSEDTAVYYC

B.473 GGTF....SSYPISWVRQAPGQGLEWMGGIIPI..LGTSNYAQKFQ.GRVTITADESTSTAYMELSSLRSEDTAVYYC

B.529 GYTF....TSYGISWVRQAPGQGLEWMGGIIPI..FGTANYAQKFQ.GRVTITADESTSTAYMELSSLRSEDTAVYYC

B.719 GYTF....TSYGISWVRQAPGQGLEWMGGIIPI..FGTANYAQKFQ.GRVTITADESTSTAYMELSSLRSEDTAVYYC

B.740 GYTF....TSYGISWVRQAPGQGLEWMGGIIPI..FGTANYAQKFQ.GRVTITADESTSTAYMELSSLRSEDTAVYYC

B.1487 GYTF....TSYGISWVRQAPGQGLEWMGGIIPI..FGTANYAQKFQ.GRVTITADESTSTAYMELSSLRSEDTAVYYC

B.2937 GYTF....TSYGISWVRQAPGQGLEWMGGIIPI..FGTANYAQKFQ.GRVTITADESTSTAYMELSSLRSEDTAVYYC

B.3079 GYTF....TSYGISWVRQAPGQGLEWMGGIIPI..FGTANYAQKFQ.GRVTITADESTSTAYMELSSLRSEDTAVYYC

B.3234 GYTF....TSYGISWVRQAPGQGLEWMGGIIPI..FGTANYAQKFQ.GRVTITADESTSTAYMELSSLRSEDTAVYYC

B.3398 GYTF....TSYGISWVRQAPGQGLEWMGGIIPI..FGTANYAQKFQ.GRVTITADESTSTAYMELSSLRSEDTAVYYC

B.3414 GYTF....TSYGISWVRQAPGQGLEWMGGIIPI..FGTANYAQKFQ.GRVTITADESTSTAYMELSSLRSEDTAVYYC

B.3617 GYTF....TSYGISWVRQAPGQGLEWMGGIIPI..FGTANYAQKFQ.GRVTITADESTSTAYMELSSLRSEDTAVYYC

B.3631 GYTF....TSYGISWVRQAPGQGLEWMGGIIPI..FGTANYAQKFQ.GRVTITADESTSTAYMELSSLRSEDTAVYYC

B.561 GGTF....SSYTISWVRQAPGQGLEWMGGIIPI..FGTANYAQKFQ.GRVTITADESASTAYMEVSSLRSEDTAVYYC

B.579 GGTF....SRYAISWVRQAPGQGLEWMGGIIPM..FGTANYAQRFQ.GRVTITADESTSTAYMELSSLRSEDTAVYYC

B.607 GGTF....SSYTISWVRQAPGQGLEWMGRIIPI..LGIANYAQKFQ.VRVTITADKSTSTAYMELSSLRSEDTAVYYC

B.613 GGTF....STYAISWVRQAPGQGFEWLGGIIPI..FGTANYAQKFQ.GRVTITADESTSTAYMELSSLRSEDTAVYYC

B.670 GGTF....SSYAISWVRQAPGQGLEWMGGIIPI..FGTANYAQKFQ.GRVTITADESTSTAYMELSSLTSDDTSVYYC

B.694 GGTF....SRYTISWVRQAPGQGLEWMGRIIPI..LGTANYAQKFQ.GRVTITADKSTSTAYMELSSLRSEDTAVYYC

B.752 GGTF....SSYAISWVRQAPGQVLEWMGRIIPI..LGIANYAQKFQ.GRVTITADKSTSTAYMELSSLRSEDTAVYYC

B.803 GGTF....SSYAISWVRQAPGQGLEWMGRIIPI..LGIANYAQKFQ.GRVTITADKSMSTAYMELSSLRSEDTAVYYC

B.847 GGTF....SNYAISWVRQAPGQGLEWMGGIIPL..FGTANYAQKFQ.GRVTITADESTRTAYMELSSLRSEDTAVYYC

B.868 GGTF....SSYDISWVRQAPGQGLEWMGGIIPI..FGTANYAQKFQ.GRVTISADESTSTVYMELSSLRSEDTAVYYC

B.870 GGTF....SSYAISWVRQAPGQGLEWMGGIIPI..FGTANYAQNFQ.GRVTIIADESTSTAYMELSSLRSDDTAVYYC

B.894 GGSF....SNYAISWVRQAPGQGLEWMGGIIPI..FGTANYAQKFQ.GRVTITADESTSTAYMELSSLRCEDTAVYYC

B.938 GGTF....SNYAISWVRQAPGQGLEWMGGIIPL..FGTPNYAQKFQ.GRVTITADESTSTAYMELSSLRSEDTAVYYC

B.939 GGTF....SSYTISWVRQAPGQGLEWMGGITPI..FGSANYAQKFQ.GRVTITADESTSTAYMELSSLRSEDTAVYYC

B.950 GGTF....SSYTISWVRQAPGQGLEWMGGITPI..FGTPNYEQKFQ.GRVTISADESTSTAYMELSSLRSEDTAVYYC

B.952 GDTF....SSYAISWVRQAPGQGLEWMGGIIPI..FGASNYAQKFQ.GRVTITADESTSTAYMELSSLRSEDTAVYYC

B.963 GGTF....SSYAISWVRQAPGQGLEWMGGIIPL..FGTANYAQKFQ.GRVTITADESTSTAYMEVRSLRSEDTAVYYC

B.965 GGTF....SKYAISWVRQAPGQGLEWMGGIIPI..FGTANYAQKFQ.GRVTITADESTSTAYMEVNSLRSEDTAVYYC

B.968 GGTF....SSYAISWVQQAPGQGLEWMGGIIPI..FGTANYAQKFQ.GRVTITADESTSTAYMELNSLRAEDTAVYYC

B.1006 GGTF....SSYAISWVRQAPGQGLEWMGGIIPI..FGTANYAQKFQ.GRVTITADESTGTAYMELNSLRSEDTALYYC

B.1078 GGTF....SSSAISWVRQAPGQGLEWMGGIIPI..FRTADYAQKFQ.GRVTITADESTSTAYMELSSLRSEDTAVYYC

B.1091 GGTF....SGYAISWVRQAPGQGLEWMGGIIPL..FGTANYAQKFQ.GRVTITADESTSTAYMEVSSLRSEDTAVYYC

B.1105 GGTF....SSYAISWVRQAPGHGLDWMGGIIPI..FGTAFYAQTFQ.GRVTITADKSTSTAYMELSSLRSEDTAVYYC

B.1144 GGTF....SSYVINWVRQAPGQGLEWMGGFIPI..FGTANYAQKFQ.GRVTITADESTSTAYMELSSLRSEDTAVYYC

B.1186 RGTF....SSYAISWVRQAPGQGLEWMGGIIPI..FGTANYAQKFQ.GRVTITADESTSTAYMELSSLRPDDTAVYYC

B.1189 GGAF....SSYAISWVRQAPGQGLEWMGGIIPI..FGTAKYAQKFQ.GRVTITADESTSTAYMELCSLRSEDTAVYYC

B.1194 GGTF....SRYALSWVRQAPGQGLEWMGGIIPI..FGTANYAQKFQ.GRVTITADESTSTVYMELSSLRSEDTAVYYC

B.1289 GGTF....SSYAISWVRQAPGQGLEWMGGIIPI..FGRANYAQKFQ.GRVTITADESTSTAYMELRSLRSEDTAIYYC

B.1293 GGTF....SSSAISWVRQAPGQGLEWMGGIIPI..FGTANYAQKFQ.GRVTITADESTTTAYMELRSLRSEDTAVYYC

B.1305 GGTF....SRYAISWVRQAPGQGLEWMGGITPI..FGTAKYAQKFQ.GRVTITADASTSTAYMELSSLRSEDTAVYYC

B.1311 GGTF....SSYPISWVRQAPGQGLEWMGGIIPI..FGTANYAQKYQ.GRVTITADVSTSTAYMDLRSLRSEDTAVYYC

B.1324 GGTF....SRYAISWVRQAPGQGLEWMGGIIPI..FGTANYAQKFQ.GRVTITADESTSTAYMELSSLKSDDTAVYYC

B.1424 GGTF....SSYAISWVRQAPGQGLEWMGGIIPI..FGTANYAQKFQ.GRVTITADESTTTAYMEMTSLRSEDTAVYYC

B.1431 GGTF....SSYAISWVRQAPGQGLEWMGGIIPI..FGTRNYAQKFQ.GRVTITADELTITAYMELSSLRSEDTAVYYC

B.1442 GGTF....SSYAISWMRQAPGQGLEWMGGIIPI..FDKPNYAQKFQ.GRVTITADKSTSTAYMELSSLRSEDTAVYYC

B.1477 GGTF....SSYAISWVRQAPGQGLEWMGGIIPI..FGTTTYPQKFQ.GRVTITADKFTSTAYMELSSLRSEDTAVYYC

B.1504 GGTF....SSDAISWVRQAPGQGLEWMGRIIPI..LGIANYAQKFQ.GRVTITADKSTSTAYMELSSLRSEDTAVYYC

B.1524 GGTF....SSYAISWVRQAPGQGLEWMGRINPI..FGTANYAQKFQ.GRVTITADESTSTAYVELSSLRSEDTAVYYC

B.1552 GGTF....SSYAIDWVRQAPGQGLEWMGGIIPI..FGKPNYAQKFQ.GRVTITADESTSTAYMELSSLRSEDTAVYYC

B.1574 GGSF....STYAINWVRQAPGQGLEWMGGIIPI..FGTANYAQKFQ.GRVTITADESTSTAYMELSSLRSEDTAVYYC

B.1611 GGFF....SSYAISWVRQAPGQGLEWMGGIIPI..FGATKYAQKFQ.DRVTLTADKSTSTAYMELSSLRSEDTAVYYC

B.1624 GGTF....SSSAISWVRQAPGQGLEWMGGIIPL..FGTANYAQKFQ.GRVTITADESTTTAYMELSSLRSEDTAVYYC

B.1782 GGTF....SSSAISWVRQAPGQGLEWMGGIIPL..FGTANYAQKFQ.GRVTITADESTTTAYMELSSLRSEDTAVYYC

B.1846 GGTF....SSSAISWVRQAPGQGLEWMGGIIPL..FGTANYAQKFQ.GRVTITADESTTTAYMELSSLRSEDTAVYYC

B.1629 GGTF....SSSAISWVRQAHGQGLEWMGGIIPL..FGTANYAQKFQ.GRVTITADESTSTAYMELSSLRSEDTAVYYC

B.1635 GGTF....SSSAISWVRQAPGQGLEWMGGIIPL..FGTANYAQKFQ.GRVTITADESTSTAYMELSSLRSEDTAVFYC

B.1647 GGTF....STYAISWVRQAPGQGLEWVGGIIPI..FGRANYAQKFQ.GRVTITADESTSTAYMELSSLRSEDTAVYYC

B.2712 GGTF....STYAISWVRQAPGQGLEWVGGIIPI..FGRANYAQKFQ.GRVTITADESTSTAYMELSSLRSEDTAVYYC

B.1649 GGTF....SSYAISWVRQAPGQGLEWVGGIIPI..FGTANYAQNFQ.GRVTITADESTTTAYMELSSLRSEDTAVYYC

B.1657 GGTF....SSSAISWVRQAPGQGLEWMGGIIPL..FGTANYAQKFQ.GRVTITADESTSTAYMELTSLRSEDTAVYYC

B.1665 GGTF....SSSAISWVRQAPGQGLEWMGGIIPL..FGTANYAQKFQ.GRVTITADESTSTAYMELNSLRSEDTAVYYC

B.1808 GGTF....SSSAISWVRQAPGQGLEWMGGIIPL..FGTANYAQKFQ.GRVTITADESTSTAYMELNSLRSEDTAVYYC

B.1667 GGTF....SSYAIRWVRQAPGQGLEWMGGIIPI..FGTANYAQKFQ.GRVTITADGSTSTAYMELTSLRSEDTAMYYC

B.1678 GGTF....SSSAISWVRQAPGQGLEWMGGIIPL..FGTANYAQKFQ.GRVTITADESTSTVYMELSSLRSEDTAVYYC

B.1774 GGTF....SSSAISWVRQAPGQGLEWMGGIIPL..FGTANYAQKFQ.GRVTITADESTSTVYMELSSLRSEDTAVYYC

B.1873 GGTF....SSSAISWVRQAPGQGLEWMGGIIPL..FGTANYAQKFQ.GRVTITADESTSTVYMELSSLRSEDTAVYYC

B.1680 GGTF....SSYAISWVRQAPGQGLEWVGGIIPI..FATANYAQMFQ.GRVTITADVSTSTAYMELSSLRSEDTAVYYC

B.1695 GGTF....SSSAISWVRQAPGQGLEWMGGIIPL..FGTANYAQQFQ.GRVTITADESTSTAYMELSSLRSEDTAVYYC

B.1707 GGTF....SSYAISWVRQAPGQGLEWMGGIIPL..FGTANYAQKFQ.GRVSITADESTSTAYMELSSLRAEDTAVYYC

B.1738 GGTF....SSSAISWVRQAPGQGLEWMGGIIPL..FGTANYAQKFQ.GRVTITADESTSTAYMELSSLKSEDTAVYYC

B.2728 GGTF....SSSAISWVRQAPGQGLEWMGGIIPL..FGTANYAQKFQ.GRVTITADESTSTAYMELSSLKSEDTAVYYC

B.1750 GGTF....SSYAISWVRQAPGQGLEWVGGIIPL..FGKANYAQKFQ.GRVTITADESTSTAYMELSSLRSEDTAVYYC

B.1761 GGTF....SSYAISWVRQAPGQGLEWVGGIIPI..FGTANYAQKFQ.GRVTIIADESTSTAYMELSSLRSDDTAVYYC

B.1787 GGTF....SSYTISWVRHAPGQGLEWMGGIIPI..FGTADYAQKFQ.GRVTITADESTSTAYMELSSLRSEDTAVYYC

B.1793 GGTF....SSSAISWVRQAPGQGLEWMGGIIPL..FGTANYAQKFQ.GRVTITADESTSTAYMEVSSLRSEDTAVYYC

B.1797 GGTF....SSYAISWVRQAPGQGLEWMGGIIPM..FGTGNYAQKFQ.GRVTITADESTSTAYMELSSLRPEDTAVYYC

B.1804 GGNF....RSYAISWVRQAPGQGLEWVGGIIPI..FGTANYAQKFQ.GRVTITADESTSTAYMELSSLRSEDTAVYYC

B.1809 GGTF....SSYAISWVRQAPGQGLEWMGGVIPI..FGSANYAQKFQ.GRVTITADESTSTAYMELSSLRSEDTAVYFC

B.1821 GGTF....SSYAISWVRQAPGQGLEWMGGIIPI..FGTSNYAQRFQ.GRVTITADKSTRTAYMELSSLRSDDTAVYYC

B.1833 GGTF....SSYAISWVRQAPGQGLEWVGGIIPI..FGTANYAQKFQ.GRVTITADESTRTAYMELSSLTSEDTAVYYC

B.1845 GGTF....SSSAISWVRQAPGQGLEWVGGIIPI..FGRANYAQKFQ.GRVTITADESTSTAYMELSSLRSEDTAVYYC

B.1862 GGTF....SSYAISWVRQAPGQGLEWVGGIIPI..FGTANYAQKFQ.GRVTITADESTSTPYMELTSLRSEDTAVYYC

B.1880 GGTF....SSYAISWLRQAPGQGLDWVGGIIPI..FGTANYAQKFQ.GRVTITADESTSTAYMELSSLRSEDTAVYYC

B.1882 GGTF....SSYVISWVRQAPGQGLEWVGGIIPT..FGTANYAQKFQ.GRVTITADESTSTAYMELSSLRSEDTAVYYC

B.1887 GGTF....SSQAISWVRQAPGQGLEWMGGIIPI..FGRANYAQKFQ.DRVTITADESTSTAHMELSSLRSEDTAIYYC

B.1925 GGTF....SSYAISWVRQAPGQGLEWMGGIIPI..FGTTNYAQKFQ.GRVTITADESTSTAYMELSSLKSEDTAIYYC

B.1953 GGTF....RSYAISWVRQAPGQGLEWMGGIIPI..FGTANYAQKFQ.GRVTIIADESTTTAYMELSSLRSEDTAVYYC

B.1970 GGTF....SRYAISWVRQAPGQGLEWMGGIIPI..FGTANYEQKFQ.GRVTITADESTNTAYMELSSLRSEDTAVYYC

B.1971 GGTF....SSSTISWVRQAPGQGLEWMGGIIPM..FGTAHYAQKFQ.GRVTITADKSTSTAYMELSSLRSEDTAVYYC

B.1983 GGTF....SSYAINWVRQAPGQGLEWMGGIIPM..FGTAHYAQKFQ.GRVTITADESTSTAYMELSSLRSEDTAVYYC

B.1989 GGTF....SSYTITWVRQAPGQGLEWMGGIIPM..FGTANYAQKFQ.GRVTITADDSTSTAYMELSSLRSEDTAVYYC

B.1994 GGTF....S.YAITWVRQAPGQGLEWMGGIIPI..FGTPNYAQKFQ.GRVTITADESTSTAYMELSSLRSEDTAVYYC

B.1998 GGTF....STYPISWVRQAPGQGLEWMGGIIPI..FGTANYAQKFQ.GRVTITADESTSTAYMELSGLRSEDTAVYYC

B.2010 GGTF....SSYAMHWVRQAPGQRLEWMGGIIPI..FGTANYAQKFQ.GRVTITADESTSTAYMELSSLRSEDTAVYYC

B.2016 GGTF....SSYAITWVRQAPGQGLEWMGGIIPL..FGTANYAQRFQ.GRVTITADESTSTAYMELSSLRSEDTAVYYC

B.2017 GGTF....SSYAISWVRQAPGQGLEWMGGIIPI..FGTANYAPKFQ.GRVTITADESTSTAYLELSSLRSEDTAMYYC

B.2022 GGTF....SRYAISWVRQAPGQGLEWMGGIIPI..FGTANYAQKFQ.GRVTITADESTNTAYMELSSLRSEDTAMYYC

B.2027 GGTF....SKYAINWVRQAPGQGLEWMGGIIPI..FGTASYAQKFQ.GRVTITADESTSTAYMELSSLRSEDTAVYYC

B.2050 GGTF....SNYTISWVRHAPGQGLECMGGIIPI..FGTPNYAQKFQ.GRVTITADKSTSTAYMELSSLRSEDTAVYYC

B.2059 GGTF....SSYAISWVRQAPGQGLEWMGGVIPM..FGTANYAQKFQ.IRVTITADKSSSTAYIELSSLRSEDTAVYYC

B.2061 GGTF....SSYAISWVRQAPGQGLEWMGGIIPI..FGTANYAQKFQ.GRVTITADESTSTAYMELSSLTSDDTAVYFC

B.2078 GGTF....SSYAISWVRQAPGQGLEWMGGIIPI..FGTANYAQKFQ.GRVTISADESTTTAYMELSSLRFEDTAVYYC

B.2091 GYTF....TSYAISWVRQAPGQGLEWMGGIVPI..FGTANYAQKFQ.GRVTITADESTSTAYMELSSLRSEDTAVYYC

B.2096 GGTF....SSYTISWVRQAPGQGLEWMGGIIPI..FAVANYAQKFQ.GRVTITADESTSTAYMELSSLRSEDTAVYYC

B.2106 GGTF....SRYAISWVRQAPGHGLEWMGGIIPI..FGTANYPQKFQ.GRVTITADESTSTAYMELSSLRSEDTAVYYC

B.2135 GGTF....SSYVISWVRQAPGQGLEWMGRIIPI..LGIANYAQKFQ.GRVTITADKSTSTAYMELSSLRSEDTAVYYC

B.2165 GGTF....SNYAISWVRQAPGQGLEWMGGIIPI..FGTANYAQKFQ.GRVTITADESTSTVYMEVSSLRSEDTAVYYC

B.2172 GGTF....SSHAISWVRQAPGQGLEWMGGITPL..FGRANYAQKFQ.GRVTITADKSTSTAYMELSSLRSEDTAVYYC

B.2275 GGTF....SNYAINWVRQAPGQGLEWMGGIIPI..FGRANYAQKFQ.GRVTITADESTSTAYMELSSLRSEDTAVYYC

B.2409 GGTF....SNYAINWVRQAPGQGLEWMGGIIPI..FGRANYAQKFQ.GRVTITADESTSTAYMELSSLRSEDTAVYYC

B.2292 GGTF....SSYAISWVRQAPGQGLEWMGGIIPI..FGTANYAQKFQ.GRVTITADESTRTAYMDLSSLRFEDTAVYYC

B.2308 GGTF....SSYAISWVRQAPGQGLEWMGRIIPI..FDTTNYAQRFQ.GRVTITADKSTSTAYMELSSLRSEDTAVYYC

B.2341 GGTF....SSYAISWVRQAPGQGLEWMGGIIPI..FGSANYAQKFR.GRVTITADESTNTAYMELSSLRSEDTAVYYC

B.2357 GGTF....SSYAINWVRQAPGQGLEWMGEIIPI..FGKGNYAQKFQ.GRVTITADKSTSTAYMELSSLRSEDTAVYYC

B.2388 GGTF....SSYAISWVRQAPGQGLEWMGGIIPI..FGTANYAQKFQ.GRVTITADKSTNTAYMELSRLTSEDTAVYFC

B.2399 GGTF....SNHAITWVRQAPGQGLEWMGGIIPI..FGTANYAQKFQ.GRVTITADESTSTAYMELSSLRSEDTAVYYC

B.2403 GGTF....SSYGISWVRQAPGQGLEWMGGIIPI..FGTAHYAQKFQ.GRVTINADESTSTAYMELSSLRSEDTAVYYC

B.2406 GYTF....TSYGISWVRQAPGQGLEWMGGIIPI..FGTANYAQKFQ.GRVTITADKSTSTAYMELSSLRSEDTAIYYC

B.2412 GGTF....SRYAISWVRQAPGQGLEWMGRIIPI..LGIANYAQKFQ.GRVTITADKSTSTAYMELSSLRSEDTAVYYC

B.2448 GGTF....SSYAISWVRQAPGQGLECMGRIIPI..LGIANYAQKFQ.GRVTITADKSTSTAYMELSSLRSEDTAVYYC

B.2475 GGTF....SSYTINWVRQAPGQGLEWMGGIIPI..FGTANYAQKFQ.GRVTITADESTSTAYLELSSLRSEDTAVYYC

B.2517 GGTF....SSYAISWVRQAPGQGIEWMGRIIPI..LGIANYAQKFQ.GRVTITADKSTSTAYMELSSLRSEDTAVYYC

B.2561 GGTF....STYAIDWVRQAPGQGLEWMGGITPI..FGTANYAQKFQ.GRVTITADKSTTTAYMELSSLRSEDTAVYYC

B.2572 GGTF....SRYTISWVRQAPGQGLEWMGGITPI..FGTANYAQKFQ.GRVTITADESTSTAYMELSSLRSEDTAVYYC

B.2610 GGTF....SSYVISWVRQAPGQGLEWMGGIIPI..FGPANHAQKLQ.GRVTITADESTSTAYMELSSLRSDDTAVYYC

B.2674 GGTF....SSYVISWVRQAPGQGLEWMGGIIPI..FGPANHAQKLQ.GRVTITADESTSTAYMELSSLRSDDTAVYYC

B.2630 GGTF....SRYAISWVRQAPGQGLEWMGGIIPM..FGTANYAQKFQ.GRVTITADESTTTAYMELSSLRSEDTAVYYC

B.2695 GGTF....SSYAISWVRQAPGQGLEWVGGIIPL..FGTANYAQKFQ.GRVTITADESTTTAYMELSSLRSEDTAVYYC

B.2696 GGTF....SSYAISWVRQAPGQGLEWMGGIIPI..FGTANYAQKFQ.GRVTITADESTTTAYMDLSSLRSDDTAVYYC

B.2701 GGTF....NTYAISWVRQAPGQGLEWMGGIIPI..FGTANYAQKFQ.GRVTITADESTSTAYMELSGLRSEDTAVYYC

B.2704 GGTF....SSYAISWVRQAPGQGLEWMGGIIPI..FGTANYAQKFQ.GRVTITADESTNTAYMELASLRSDDTAVYYC

B.2710 GGTF....STYAISWVRQAPGQGLEWMGGIIPL..FGTANYAQKFQ.GRVTITADESTTTAYMELSSLRSEDTAVYYC

B.2717 GGTF....SSSAISWVRQAPGQGLEWMGGIIPI..FGTANYAQKFQ.GRVTITADESTSTAYMELTSLRSEDMAVYYC

B.2719 GGTF....SSSAISWVRQAPGQGLEWMGGIIPL..FGTANYAQKFQ.GRVTITADEPTSTAYMELSSLRSEDTAVYYC

B.2722 GGTF....NSYAISWVRQAPGQGLEWMGGIIPI..FGTTNYAQKFQ.GRVTITADESTRTAYMELSSLRSEDTAVYYC

B.2724 GGTF....SNSAISWVRQAPGQGLEWMGGIIPL..FGTANYAQKFQ.GRVTITADESTSTAYMELSSLRSEDTAVYYC

B.2726 GGTF....SSSAISWVRQAPGQGLEWMGGIIPL..FGTANYAQKFQ.GRVTITADESTNTAYMELSSLRSEDTAVYYC

B.2731 GGTF....SRFAISWVRQAPGQGLEWVGGIIPI..FGTANYAQKFQ.GRVTITADESTSTAYMELSSLRSEDTAVYYC

B.2738 GGTF....SRYAISWVRQAPGQGLEWMGGIMPM..FGTANYAQKFQ.GRVTITADESTSTAYMELSSLRSEDTAVYYC

B.2740 GGTF....SSSAISWVRQAPGQGLEWMGGIMPI..FGTANYAQKFQ.GRVTITADESTSIAYMELSSLRSEDTAVYYC

B.2748 GGTF....SSSAISWVRQAPGQGLEWMGGIIPL..FGTANYPQKFQ.GRVTITADESTSTAYMELSSLRSEDTAVYYC

B.2756 GGTF....STYAISWVRQAPGQGLEWMGGIIPI..FGTANYAQKFQ.GRVTITADTSTSTTYMELSSLTSEDTAVYYC

B.2808 GGTF....STYAISWVRQAPGQGLEWMGGIIPI..FGTANYAQKFQ.GRVTITADTSTSTTYMELSSLTSEDTAVYYC

B.2760 GGTF....SSYAISWVRQAPGQGLEWMGGIIPI..FDTANYAQKFQ.GRVTISADKSTRTAYMELSSLTSEDTAVYYC

B.2781 GGTF....STYAISWVRQAPGQGLEWMGGIIPI..FGTANYAQKFQ.GRVTITADTSTSTAFMELSSLTSEDTAVYYC

B.2788 GGTF....SSYAISWVRQAPGQGLEWMGGIIPI..FGTANYAQKFQ.GRVTITADRSTSTAYMELNSLTSADTAVYYC

B.2789 GGTF....SSYAISWVRQAPGQGPEWMGGIIPI..FGTANYAQKFQ.GRVTITADTSTSTAYMELNSLTSEDTAVYYC

B.2793 GGTF....STYAISWVRQAPGQGLEWMGGIIPI..FGTANYAQKFQ.GRVTITADRSTSTAYMELSGLRSEDTAIYYC

B.2796 GGTF....STYAISWVRQAPGQGLEWMGGIIPI..FGTANYAQKFQ.GTVTITADKSTSTAYMELSSLISEDSAVYYC

B.2801 GGTF....SLYAISWVRQAPGQGLEWMGGIIPM..FGTSNYAQKFQ.GRVTITADKSTSTAYMELNSLRSEDTAVYYC

B.2803 GGTF....SNYAINWVRQAPGQGLEWMGGIIPI..FGTANYAQKFQ.GRVTITADKSTNTAYMDLSSLRSEDTAVYYC

B.2817 GGTF....STYAISWVRQAPGQGLEWMGGIIPI..FGTANYAQKFQ.GRVTITADTSTSTAYMELNSLTSEDTAVYYC

B.2827 GGTF....SSNAISWVRQAPGQGLEWMGGIIPM..FGTANYAQKFQ.GRVTITADTSTSTAYMELSSLRSEDTAVYFC

B.2830 GGTF....SSYAISWVRQAPGQGPEWMGGIVPI..FGTANYAQTFR.GRVTITADKSTSTAYMELSSLRSEDTAVYYC

B.2839 GGTF....SSYAISWVRQAPGQGPEWMGGIIPL..FGTANYAQNFQ.GRVTITADKSTSTAYMELSSLRSEDTAIYYC

B.2843 GGTF....STSAISWVRQAPGQGLEWVGGIIPI..FGTANYAQKFQ.GRVTITADESTSTAYMELSSLRSEDTAVYYC

B.2851 GGTF....STYAISWVRQAPGQGLEWMGGIIPI..FGTANYAQKFQ.GRVTITADTSTSTAYMELSSLTSEDTAVYFC

B.2852 GGTF....SSYAISWMRQAPGQGPEWMGGIIPI..FGTANYAQKFQ.GRVTITADKSTNTAYMDLSSLRSEDTAVYYC

B.2854 GGTF....SNYAISWVRQAPGQGLEWMGGIIPI..FGTANYAQKFQ.GRVTITADKSTSTAYMELNSLRFEDTAIYYC

B.2860 GGTF....NTYAISWVRQAPGQGLEWMGGIIPI..FGTANYAQKFQ.GRVTITADTSTSTAYMELSSLTSEDTAVYYC

B.2864 GGTF....GNYAISWVRQAPGQGLEWMGGIIPI..FGTGNYAQKFQ.GRVTITADTSTSTAYMELSSLRSEDTAVYYC

B.2873 GGTF....SSYAISWVRQAPGQGLEWMGRIIPI..LGIANYAQKFQ.GRVTITADKSTTTAYMELSSLRSEDTAVYYC

B.2977 GGTF....SRYGISWVRQAPGQGLEWMGGTIPI..FGTAKYAQKFQ.GRVTITADKSTSTAYMELSSLRSEDTAVYYC

B.2982 GGTF....SSYVMTWVRQAPGQGLEWMGGIIPI..FGTANYAQKFQ.GRVTITADESTSTAYMELSSLRSEDTAVYYC

B.2994 GGTF....SNYAISWVRQAPGQGLEWMGGIIPI..FGASNYAQKFQ.GRVTITADESTSTAYMELSSLRSEDTAVYYC

B.2999 GGTF....SNYAISWVRQAPGLGLEWMGGIIPI..FGTPNYAQKFQ.GRVTITADESTSTAYMELSSLRSEDTAVYYC

B.3011 GGTF....SNFAISWVRQAPGQGLEWMGGIIPI..FGTANYARKFQ.GRVTITADDSTSTAYMELSSLRSEDTAVYYC

B.3033 GGTF....SSYATSWVRQAPGQGLEWMGRIIPI..LGIANYAQKFQ.GRVTITADKSTSTAYMELSSLRSEDTAVYYC

B.3047 GGTF....SSYAISWVRQAPGQGLEWMGRIIPI..LGIANYAQKFQ.GRVTITADKSTSTVYMELSSLRSEDTAVYYC

B.3054 GGTF....SSYAISWVRQAPGQGLEWMGGIIPI..LGRANNAQKFQ.GRVTITADKSTSTAYMELSSLRFEDTAVYYC

B.3067 GDTF....STYGISWVRQAPGQGLEWMGGIIPI..FGTVNYAQKFQ.GRVTITADKSTSTAYMELSSLRSEDTAVYYC

B.3124 GGTF....SSYAISWVRQAPGQGLEWMGGIIPT..FGSPNYAQKFQ.GRVTITADESTSTAYMELSSLRSEDTAVYYC

B.3146 GGSF....TSYAINWVRQAPGQGLEWMGGIIPI..FGTANYAQKFQ.GRVTITADESTSTAYMELSSLRSEDTAVYYC

B.3148 GGTF....TNYAIDWVRQAPGQGLEWMGGIIPI..FGTANYAQKFQ.GRVTITADESTSTAYMELSSLRSEDTAVYYC

B.3171 GYTF....SDYYIHWVRQAPGQGLEWMGGIIPI..FGTANYAQKFQ.GRVTITADKSTSTAYMELSSLRSEDTAVYYC

B.3197 GYTF....SSYDINWVRQAPGQGLEWMGGIIPI..FGTANYAQKFQ.GRVTITADESTSTAYMELSSLRSEDTAVYYC

B.3218 GGTF....SSYAISWVRQAPGQGLEWMGGIIPI..FGTANYAQKLQ.GRVTMTTDTSTSTAYMELRSLRSDDTAVYYC

B.3272 GGTF....SSNAIRWVRQAPGQGLEWMGGIIPI..FGTANYAQKFQ.GRVTITADKSTSTAYMDLSSLRSDDTAVYYC

B.3287 GGTF....SSYAISWVRQAPGQGLEWMGRIIPI..LGIANYAQKFQ.GRVTITADKSTSTAYMELSSLRS.DTAVYYC

B.3324 GYTF....SDYNIHWVRQAPGQGLEWMGGIIPI..FGTANYAQKFQ.GRVTITADKSTSTAYMELSSLRSEDTAVYYC

B.3333 GGTF....SSYAIRWVRQAPGQGLEWMGRIIPI..LGIANYAQKFQ.GRVTITADKSTSTAYMELSSLRSEDTAVYYC

B.3374 GGTF....SSYAISWVRQAPGQGLEWMGRIIPI..LGIANYAQKFQ.GRVTITADESTSTAYMELSSLRSEDTAVYYC

B.3386 GGTF....SSYAISWVRQAPGQGLEWMGGIIPI..FGIANYAQKFQ.GRVSTTADDSTSTAYMELSSLRSEDTAVYYC

B.3408 GGTF....SSYSISWVRQAPGQGLEWMGRIIPI..LGIANYAQKFQ.GRVTITADKSTSTAYMELSSLRSEDTAVYYC

B.3438 GGTF....SSYAINWVRQAPGQGLEWVGGIIPI..FRTANYAQKFQ.GRVTITADESTSTAYMELSSLRSEDTAVYYC

B.3472 GGTF....SSYAISWVRQAPGQGLEWMGGIIPI..FGTVNYAQKFQ.GRVTITADESTSTAYMELSRLRFEDTAVYYC

B.3512 GGTF....SSYAISWVRQAPGQGLEWMGGIIPI..FGTTNYVQKFQ.DRVTITADKSTNTVYMELSSLGSEDTAVYYC

**Supplementary Data S2:**

The total IGHV1-69 repertoire from the single-cell RNA sequencing data (Fig. 4; n=351 with chronic disease and n=373 with SVR) was aligned to a published sequence dataset of 144 validated HCV-neutralizing antibodies [1]. All Sequences were first gapped according to their germline alignment, then levenshtein distance was calculated for each pair of sequences. Nucleotide sequences represent cell barcodes.

**A. Alignment with two exchanges_________________________________________**

**Alignments with two exchanges:**

Weber et. al Sequences 1198_01_E11 and 1198_03_G07

GGTF....SSYAISWVRQAPGQGLEWMGGIIPI..FGTANYAQKLQ.DRVTITADKSTSTAYMELSSLRSEDTAVYYC

Aligned Sequences (n=92) from our cohort

hcv23_AAAGATGAGCGTTTAC-1 GGTF....SSYAISWVRQAPGQGLEWMGGIIPI..FGTANYAQKFQ.GRVTITADKSTSTAYMELSSLRSEDTAVYYC

hcv23_AAAGTAGGTACTTGAC-1 GGTF....SSYAISWVRQAPGQGLEWMGGIIPI..FGTANYAQKFQ.GRVTITADKSTSTAYMELSSLRSEDTAVYYC

hcv23_AACTCCCTCAGGTTCA-1 GGTF....SSYAISWVRQAPGQGLEWMGGIIPI..FGTANYAQKFQ.GRVTITADKSTSTAYMELSSLRSEDTAVYYC

hcv23_ACCAGTAAGGTAGCCA-1 GGTF....SSYAISWVRQAPGQGLEWMGGIIPI..FGTANYAQKFQ.GRVTITADKSTSTAYMELSSLRSEDTAVYYC

hcv23_ACGGCCAAGCCGATTT-1 GGTF....SSYAISWVRQAPGQGLEWMGGIIPI..FGTANYAQKFQ.GRVTITADKSTSTAYMELSSLRSEDTAVYYC

hcv23_ACTGAGTGTCACCTAA-1 GGTF....SSYAISWVRQAPGQGLEWMGGIIPI..FGTANYAQKFQ.GRVTITADKSTSTAYMELSSLRSEDTAVYYC

hcv23_AGAGTGGAGCGATGAC-1 GGTF....SSYAISWVRQAPGQGLEWMGGIIPI..FGTANYAQKFQ.GRVTITADKSTSTAYMELSSLRSEDTAVYYC

hcv23_AGATCTGTCGTCCAGG-1 GGTF....SSYAISWVRQAPGQGLEWMGGIIPI..FGTANYAQKFQ.GRVTITADKSTSTAYMELSSLRSEDTAVYYC

hcv23_AGCGGTCTCGTGGGAA-1 GGTF....SSYAISWVRQAPGQGLEWMGGIIPI..FGTANYAQKFQ.GRVTITADKSTSTAYMELSSLRSEDTAVYYC

hcv23_AGGCCGTAGGTGTTAA-1 GGTF....SSYAISWVRQAPGQGLEWMGGIIPI..FGTANYAQKFQ.GRVTITADKSTSTAYMELSSLRSEDTAVYYC

hcv23_AGGTCATGTCAAAGAT-1 GGTF....SSYAISWVRQAPGQGLEWMGGIIPI..FGTANYAQKFQ.GRVTITADKSTSTAYMELSSLRSEDTAVYYC

hcv23_ATCCACCAGGCCGAAT-1 GGTF....SSYAISWVRQAPGQGLEWMGGIIPI..FGTANYAQKFQ.GRVTITADKSTSTAYMELSSLRSEDTAVYYC

hcv23_ATTACTCTCAAACCAC-1 GGTF....SSYAISWVRQAPGQGLEWMGGIIPI..FGTANYAQKFQ.GRVTITADKSTSTAYMELSSLRSEDTAVYYC

hcv23_CAAGGCCCATCCTAGA-1 GGTF....SSYAISWVRQAPGQGLEWMGGIIPI..FGTANYAQKFQ.GRVTITADKSTSTAYMELSSLRSEDTAVYYC

hcv23_CACAAACCACCAGGTC-1 GGTF....SSYAISWVRQAPGQGLEWMGGIIPI..FGTANYAQKFQ.GRVTITADKSTSTAYMELSSLRSEDTAVYYC

hcv23_CACTCCATCTTGTTTG-1 GGTF....SSYAISWVRQAPGQGLEWMGGIIPI..FGTANYAQKFQ.GRVTITADKSTSTAYMELSSLRSEDTAVYYC

hcv23_CATATGGAGCGTCAAG-1 GGTF....SSYAISWVRQAPGQGLEWMGGIIPI..FGTANYAQKFQ.GRVTITADKSTSTAYMELSSLRSEDTAVYYC

hcv23_CATCAGAAGCTGAACG-1 GGTF....SSYAISWVRQAPGQGLEWMGGIIPI..FGTANYAQKFQ.GRVTITADKSTSTAYMELSSLRSEDTAVYYC

hcv23_CCGTGGATCCCAAGTA-1 GGTF....SSYAISWVRQAPGQGLEWMGGIIPI..FGTANYAQKFQ.GRVTITADKSTSTAYMELSSLRSEDTAVYYC

hcv23_CCTACACCACATAACC-1 GGTF....SSYAISWVRQAPGQGLEWMGGIIPI..FGTANYAQKFQ.GRVTITADKSTSTAYMELSSLRSEDTAVYYC

hcv23_CGAACATCACAGTCGC-1 GGTF....SSYAISWVRQAPGQGLEWMGGIIPI..FGTANYAQKFQ.GRVTITADKSTSTAYMELSSLRSEDTAVYYC

hcv23_CGATTGAAGGATGTAT-1 GGTF....SSYAISWVRQAPGQGLEWMGGIIPI..FGTANYAQKFQ.GRVTITADKSTSTAYMELSSLRSEDTAVYYC

hcv23_CGGTTAACAATAGAGT-1 GGTF....SSYAISWVRQAPGQGLEWMGGIIPI..FGTANYAQKFQ.GRVTITADKSTSTAYMELSSLRSEDTAVYYC

hcv23_CTAACTTTCAGAGGTG-1 GGTF....SSYAISWVRQAPGQGLEWMGGIIPI..FGTANYAQKFQ.GRVTITADKSTSTAYMELSSLRSEDTAVYYC

hcv23_CTAAGACAGTGTTAGA-1 GGTF....SSYAISWVRQAPGQGLEWMGGIIPI..FGTANYAQKFQ.GRVTITADKSTSTAYMELSSLRSEDTAVYYC

hcv23_CTCGAGGGTCAAAGCG-1 GGTF....SSYAISWVRQAPGQGLEWMGGIIPI..FGTANYAQKFQ.GRVTITADKSTSTAYMELSSLRSEDTAVYYC

hcv23_CTGATAGAGTAAGTAC-1 GGTF....SSYAISWVRQAPGQGLEWMGGIIPI..FGTANYAQKFQ.GRVTITADKSTSTAYMELSSLRSEDTAVYYC

hcv23_GACACGCCAAAGCAAT-1 GGTF....SSYAISWVRQAPGQGLEWMGGIIPI..FGTANYAQKFQ.GRVTITADKSTSTAYMELSSLRSEDTAVYYC

hcv23_GACAGAGTCGACAGCC-1 GGTF....SSYAISWVRQAPGQGLEWMGGIIPI..FGTANYAQKFQ.GRVTITADKSTSTAYMELSSLRSEDTAVYYC

hcv23_GACGTGCCAGTAAGCG-1 GGTF....SSYAISWVRQAPGQGLEWMGGIIPI..FGTANYAQKFQ.GRVTITADKSTSTAYMELSSLRSEDTAVYYC

hcv23_GATCTAGGTCTGCCAG-1 GGTF....SSYAISWVRQAPGQGLEWMGGIIPI..FGTANYAQKFQ.GRVTITADKSTSTAYMELSSLRSEDTAVYYC

hcv23_GATTCAGAGGTAAACT-1 GGTF....SSYAISWVRQAPGQGLEWMGGIIPI..FGTANYAQKFQ.GRVTITADKSTSTAYMELSSLRSEDTAVYYC

hcv23_GCAGTTAAGTGCTGCC-1 GGTF....SSYAISWVRQAPGQGLEWMGGIIPI..FGTANYAQKFQ.GRVTITADKSTSTAYMELSSLRSEDTAVYYC

hcv23_GCGACCACATATGCTG-1 GGTF....SSYAISWVRQAPGQGLEWMGGIIPI..FGTANYAQKFQ.GRVTITADKSTSTAYMELSSLRSEDTAVYYC

hcv23_GGGAGATAGAAGGCCT-1 GGTF....SSYAISWVRQAPGQGLEWMGGIIPI..FGTANYAQKFQ.GRVTITADKSTSTAYMELSSLRSEDTAVYYC

hcv23_GTACTCCGTATGAAAC-1 GGTF....SSYAISWVRQAPGQGLEWMGGIIPI..FGTANYAQKFQ.GRVTITADKSTSTAYMELSSLRSEDTAVYYC

hcv23_GTCACGGGTCGACTAT-1 GGTF....SSYAISWVRQAPGQGLEWMGGIIPI..FGTANYAQKFQ.GRVTITADKSTSTAYMELSSLRSEDTAVYYC

hcv23_GTCATTTCATAGGATA-1 GGTF....SSYAISWVRQAPGQGLEWMGGIIPI..FGTANYAQKFQ.GRVTITADKSTSTAYMELSSLRSEDTAVYYC

hcv23_GTGCATAGTCGCGTGT-1 GGTF....SSYAISWVRQAPGQGLEWMGGIIPI..FGTANYAQKFQ.GRVTITADKSTSTAYMELSSLRSEDTAVYYC

hcv23_GTTCATTCACAAGTAA-1 GGTF....SSYAISWVRQAPGQGLEWMGGIIPI..FGTANYAQKFQ.GRVTITADKSTSTAYMELSSLRSEDTAVYYC

hcv23_TACGGTACACGGCTAC-1 GGTF....SSYAISWVRQAPGQGLEWMGGIIPI..FGTANYAQKFQ.GRVTITADKSTSTAYMELSSLRSEDTAVYYC

hcv23_TACTTGTTCGTGGGAA-1 GGTF....SSYAISWVRQAPGQGLEWMGGIIPI..FGTANYAQKFQ.GRVTITADKSTSTAYMELSSLRSEDTAVYYC

hcv23_TATCTCATCGTCTGAA-1 GGTF....SSYAISWVRQAPGQGLEWMGGIIPI..FGTANYAQKFQ.GRVTITADKSTSTAYMELSSLRSEDTAVYYC

hcv23_TCAATCTCAACGCACC-1 GGTF....SSYAISWVRQAPGQGLEWMGGIIPI..FGTANYAQKFQ.GRVTITADKSTSTAYMELSSLRSEDTAVYYC

hcv23_TCGCGTTGTTCCCGAG-1 GGTF....SSYAISWVRQAPGQGLEWMGGIIPI..FGTANYAQKFQ.GRVTITADKSTSTAYMELSSLRSEDTAVYYC

hcv23_TCGTAGATCGAATGGG-1 GGTF....SSYAISWVRQAPGQGLEWMGGIIPI..FGTANYAQKFQ.GRVTITADKSTSTAYMELSSLRSEDTAVYYC

hcv23_TCTGGAACATAAGACA-1 GGTF....SSYAISWVRQAPGQGLEWMGGIIPI..FGTANYAQKFQ.GRVTITADKSTSTAYMELSSLRSEDTAVYYC

hcv23_TCTTTCCAGGATCGCA-1 GGTF....SSYAISWVRQAPGQGLEWMGGIIPI..FGTANYAQKFQ.GRVTITADKSTSTAYMELSSLRSEDTAVYYC

hcv23_TGGCCAGTCGCCATAA-1 GGTF....SSYAISWVRQAPGQGLEWMGGIIPI..FGTANYAQKFQ.GRVTITADKSTSTAYMELSSLRSEDTAVYYC

hcv23_TTCCCAGTCAACCAAC-1 GGTF....SSYAISWVRQAPGQGLEWMGGIIPI..FGTANYAQKFQ.GRVTITADKSTSTAYMELSSLRSEDTAVYYC

hcv23_TTGCCGTAGTGAAGAG-1 GGTF....SSYAISWVRQAPGQGLEWMGGIIPI..FGTANYAQKFQ.GRVTITADKSTSTAYMELSSLRSEDTAVYYC

hcv23_TTGTAGGGTGGTGTAG-1 GGTF....SSYAISWVRQAPGQGLEWMGGIIPI..FGTANYAQKFQ.GRVTITADKSTSTAYMELSSLRSEDTAVYYC

hcv23_ACAGCTATCCATGCTC-1 GGTF....SSYAISWVRQAPRQGLEWMGGIIPI..FGTANYAQKFQ.DRVTITADKSTSTAYMELSSLRSEDTAVYYC

hcv23_AGCGTATGTTCTCATT-1 GGTF....SSYAISWVRQAPRQGLEWMGGIIPI..FGTANYAQKFQ.DRVTITADKSTSTAYMELSSLRSEDTAVYYC

hcv49_AAACGGGAGCCAGGAT-1 GGTF....SSYAISWVRQAPGQGLEWMGGIIPI..FGTANYAQKFQ.GRVTITADKSTSTAYMELSSLRSEDTAVYYC

hcv49_AGCGTCGCAGCTGCTG-1 GGTF....SSYAISWVRQAPGQGLEWMGGIIPI..FGTANYAQKFQ.GRVTITADKSTSTAYMELSSLRSEDTAVYYC

hcv49_ATAAGAGCATCTACGA-1 GGTF....SSYAISWVRQAPGQGLEWMGGIIPI..FGTANYAQKFQ.GRVTITADKSTSTAYMELSSLRSEDTAVYYC

hcv49_ATCACGAAGTCTCCTC-1 GGTF....SSYAISWVRQAPGQGLEWMGGIIPI..FGTANYAQKFQ.GRVTITADKSTSTAYMELSSLRSEDTAVYYC

hcv49_ATCGAGTGTAAATGAC-1 GGTF....SSYAISWVRQAPGQGLEWMGGIIPI..FGTANYAQKFQ.GRVTITADKSTSTAYMELSSLRSEDTAVYYC

hcv49_ATCTGCCAGCCGGTAA-1 GGTF....SSYAISWVRQAPGQGLEWMGGIIPI..FGTANYAQKFQ.GRVTITADKSTSTAYMELSSLRSEDTAVYYC

hcv49_ATGGGAGGTTGCTCCT-1 GGTF....SSYAISWVRQAPGQGLEWMGGIIPI..FGTANYAQKFQ.GRVTITADKSTSTAYMELSSLRSEDTAVYYC

hcv49_ATGGGAGTCATCTGTT-1 GGTF....SSYAISWVRQAPGQGLEWMGGIIPI..FGTANYAQKFQ.GRVTITADKSTSTAYMELSSLRSEDTAVYYC

hcv49_CAAGGCCGTCTAGTCA-1 GGTF....SSYAISWVRQAPGQGLEWMGGIIPI..FGTANYAQKFQ.GRVTITADKSTSTAYMELSSLRSEDTAVYYC

hcv49_CAAGTTGTCGCCGTGA-1 GGTF....SSYAISWVRQAPGQGLEWMGGIIPI..FGTANYAQKFQ.GRVTITADKSTSTAYMELSSLRSEDTAVYYC

hcv49_CAGCCGATCTGGAGCC-1 GGTF....SSYAISWVRQAPGQGLEWMGGIIPI..FGTANYAQKFQ.GRVTITADKSTSTAYMELSSLRSEDTAVYYC

hcv49_CAGCTAATCCTCCTAG-1 GGTF....SSYAISWVRQAPGQGLEWMGGIIPI..FGTANYAQKFQ.GRVTITADKSTSTAYMELSSLRSEDTAVYYC

hcv49_CAGTAACCAAACCTAC-1 GGTF....SSYAISWVRQAPGQGLEWMGGIIPI..FGTANYAQKFQ.GRVTITADKSTSTAYMELSSLRSEDTAVYYC

hcv49_CATTCGCGTGTCGCTG-1 GGTF....SSYAISWVRQAPGQGLEWMGGIIPI..FGTANYAQKFQ.GRVTITADKSTSTAYMELSSLRSEDTAVYYC

hcv49_CGAGCACTCGGTCCGA-1 GGTF....SSYAISWVRQAPGQGLEWMGGIIPI..FGTANYAQKFQ.GRVTITADKSTSTAYMELSSLRSEDTAVYYC

hcv49_CTACACCGTAGGCATG-1 GGTF....SSYAISWVRQAPGQGLEWMGGIIPI..FGTANYAQKFQ.GRVTITADKSTSTAYMELSSLRSEDTAVYYC

hcv49_CTACATTAGCGGCTTC-1 GGTF....SSYAISWVRQAPGQGLEWMGGIIPI..FGTANYAQKFQ.GRVTITADKSTSTAYMELSSLRSEDTAVYYC

hcv49_CTCGGGACATATACGC-1 GGTF....SSYAISWVRQAPGQGLEWMGGIIPI..FGTANYAQKFQ.GRVTITADKSTSTAYMELSSLRSEDTAVYYC

hcv49_CTGAAACAGGCATGGT-1 GGTF....SSYAISWVRQAPGQGLEWMGGIIPI..FGTANYAQKFQ.GRVTITADKSTSTAYMELSSLRSEDTAVYYC

hcv49_GATCTAGCAAGCTGTT-1 GGTF....SSYAISWVRQAPGQGLEWMGGIIPI..FGTANYAQKFQ.GRVTITADKSTSTAYMELSSLRSEDTAVYYC

hcv49_GCCTCTAAGCAATCTC-1 GGTF....SSYAISWVRQAPGQGLEWMGGIIPI..FGTANYAQKFQ.GRVTITADKSTSTAYMELSSLRSEDTAVYYC

hcv49_GCGACCATCGGTGTCG-1 GGTF....SSYAISWVRQAPGQGLEWMGGIIPI..FGTANYAQKFQ.GRVTITADKSTSTAYMELSSLRSEDTAVYYC

hcv49_GCGCAGTTCTGGTGTA-1 GGTF....SSYAISWVRQAPGQGLEWMGGIIPI..FGTANYAQKFQ.GRVTITADKSTSTAYMELSSLRSEDTAVYYC

hcv49_GCGGGTTCACGGTAAG-1 GGTF....SSYAISWVRQAPGQGLEWMGGIIPI..FGTANYAQKFQ.GRVTITADKSTSTAYMELSSLRSEDTAVYYC

hcv49_GCTGCTTTCGTTGACA-1 GGTF....SSYAISWVRQAPGQGLEWMGGIIPI..FGTANYAQKFQ.GRVTITADKSTSTAYMELSSLRSEDTAVYYC

hcv49_GGAGCAAGTTCGTGAT-1 GGTF....SSYAISWVRQAPGQGLEWMGGIIPI..FGTANYAQKFQ.GRVTITADKSTSTAYMELSSLRSEDTAVYYC

hcv49_GTCAAGTCATGGGACA-1 GGTF....SSYAISWVRQAPGQGLEWMGGIIPI..FGTANYAQKFQ.GRVTITADKSTSTAYMELSSLRSEDTAVYYC

hcv49_GTTCTCGCAGCTCCGA-1 GGTF....SSYAISWVRQAPGQGLEWMGGIIPI..FGTANYAQKFQ.GRVTITADKSTSTAYMELSSLRSEDTAVYYC

hcv49_TACGGATCAACGCACC-1 GGTF....SSYAISWVRQAPGQGLEWMGGIIPI..FGTANYAQKFQ.GRVTITADKSTSTAYMELSSLRSEDTAVYYC

hcv49_TACTCATGTCGTTGTA-1 GGTF....SSYAISWVRQAPGQGLEWMGGIIPI..FGTANYAQKFQ.GRVTITADKSTSTAYMELSSLRSEDTAVYYC

hcv49_TACTCATGTGTAAGTA-1 GGTF....SSYAISWVRQAPGQGLEWMGGIIPI..FGTANYAQKFQ.GRVTITADKSTSTAYMELSSLRSEDTAVYYC

hcv49_TCAGCAATCAAGATCC-1 GGTF....SSYAISWVRQAPGQGLEWMGGIIPI..FGTANYAQKFQ.GRVTITADKSTSTAYMELSSLRSEDTAVYYC

hcv49_TCAGCTCAGATCTGAA-1 GGTF....SSYAISWVRQAPGQGLEWMGGIIPI..FGTANYAQKFQ.GRVTITADKSTSTAYMELSSLRSEDTAVYYC

hcv49_TCAGGTACAGCAGTTT-1 GGTF....SSYAISWVRQAPGQGLEWMGGIIPI..FGTANYAQKFQ.GRVTITADKSTSTAYMELSSLRSEDTAVYYC

hcv49_TCCCGATAGATGCCAG-1 GGTF....SSYAISWVRQAPGQGLEWMGGIIPI..FGTANYAQKFQ.GRVTITADKSTSTAYMELSSLRSEDTAVYYC

hcv49_TGAGCATGTGTGGCTC-1 GGTF....SSYAISWVRQAPGQGLEWMGGIIPI..FGTANYAQKFQ.GRVTITADKSTSTAYMELSSLRSEDTAVYYC

hcv49_TGCGGGTTCCCTCAGT-1 GGTF....SSYAISWVRQAPGQGLEWMGGIIPI..FGTANYAQKFQ.GRVTITADKSTSTAYMELSSLRSEDTAVYYC

hcv49_TGTTCCGCACCTATCC-1 GGTF....SSYAISWVRQAPGQGLEWMGGIIPI..FGTANYAQKFQ.GRVTITADKSTSTAYMELSSLRSEDTAVYYC

**B. Alignment with three exchanges_________________________________________**

Weber et. al Sequences 1198_01_E11 and 1198_03_G07

GGTF....SSYAISWVRQAPGQGLEWMGGIIPI..FGTANYAQKLQ.DRVTITADKSTSTAYMELSSLRSEDTAVYYC

Aligned Sequences (n=129) from our cohort

hcv23_AACTCCCAGCAGGCTA-1

GGTF....STYAISWVRQAPGQGLEWMGGIIPI..FGTANYAQKFQ.GRVTITADKSTSTAYMELSSLRSEDTAVYYC

hcv23_AATCGGTCAGTTTACG-1

GGTF....STYAISWVRQAPGQGLEWMGGIIPI..FGTANYAQKFQ.GRVTITADKSTSTAYMELSSLRSEDTAVYYC

hcv23_ACCCACTCATCCCACT-1

GGTF....STYAISWVRQAPGQGLEWMGGIIPI..FGTANYAQKFQ.GRVTITADKSTSTAYMELSSLRSEDTAVYYC

hcv23_AGATTGCCAAAGTCAA-1

GGTF....STYAISWVRQAPGQGLEWMGGIIPI..FGTANYAQKFQ.GRVTITADKSTSTAYMELSSLRSEDTAVYYC

hcv23_AGCTTGAAGAACTCGG-1

GGTF....STYAISWVRQAPGQGLEWMGGIIPI..FGTANYAQKFQ.GRVTITADKSTSTAYMELSSLRSEDTAVYYC

hcv23_ATAACGCCATACGCTA-1

GGTF....STYAISWVRQAPGQGLEWMGGIIPI..FGTANYAQKFQ.GRVTITADKSTSTAYMELSSLRSEDTAVYYC

hcv23_ATGAGGGCAATGAATG-1

GGTF....STYAISWVRQAPGQGLEWMGGIIPI..FGTANYAQKFQ.GRVTITADKSTSTAYMELSSLRSEDTAVYYC

hcv23_ATTCTACGTTGTGGAG-1

GGTF....STYAISWVRQAPGQGLEWMGGIIPI..FGTANYAQKFQ.GRVTITADKSTSTAYMELSSLRSEDTAVYYC

hcv23_CCACCTAGTGCGATAG-1

GGTF....STYAISWVRQAPGQGLEWMGGIIPI..FGTANYAQKFQ.GRVTITADKSTSTAYMELSSLRSEDTAVYYC

hcv23_CGCCAAGCAGCGATCC-1

GGTF....STYAISWVRQAPGQGLEWMGGIIPI..FGTANYAQKFQ.GRVTITADKSTSTAYMELSSLRSEDTAVYYC

hcv23_CGGAGTCTCCTCAATT-1

GGTF....STYAISWVRQAPGQGLEWMGGIIPI..FGTANYAQKFQ.GRVTITADKSTSTAYMELSSLRSEDTAVYYC

hcv23_CTAACTTAGACAGGCT-1

GGTF....STYAISWVRQAPGQGLEWMGGIIPI..FGTANYAQKFQ.GRVTITADKSTSTAYMELSSLRSEDTAVYYC

hcv23_CTACCCAAGGGTGTGT-1

GGTF....STYAISWVRQAPGQGLEWMGGIIPI..FGTANYAQKFQ.GRVTITADKSTSTAYMELSSLRSEDTAVYYC

hcv23_CTACGTCCACCATGTA-1

GGTF....STYAISWVRQAPGQGLEWMGGIIPI..FGTANYAQKFQ.GRVTITADKSTSTAYMELSSLRSEDTAVYYC

hcv23_GACACGCCATATGAGA-1

GGTF....STYAISWVRQAPGQGLEWMGGIIPI..FGTANYAQKFQ.GRVTITADKSTSTAYMELSSLRSEDTAVYYC

hcv23_GACCAATCAGTGAGTG-1

GGTF....STYAISWVRQAPGQGLEWMGGIIPI..FGTANYAQKFQ.GRVTITADKSTSTAYMELSSLRSEDTAVYYC

hcv23_GATTCAGAGATCGGGT-1

GGTF....STYAISWVRQAPGQGLEWMGGIIPI..FGTANYAQKFQ.GRVTITADKSTSTAYMELSSLRSEDTAVYYC

hcv23_GGACAAGCACGGTTTA-1

GGTF....STYAISWVRQAPGQGLEWMGGIIPI..FGTANYAQKFQ.GRVTITADKSTSTAYMELSSLRSEDTAVYYC

hcv23_GGACAGACAGTATAAG-1

GGTF....STYAISWVRQAPGQGLEWMGGIIPI..FGTANYAQKFQ.GRVTITADKSTSTAYMELSSLRSEDTAVYYC

hcv23_GGAGCAAAGAGGGCTT-1

GGTF....STYAISWVRQAPGQGLEWMGGIIPI..FGTANYAQKFQ.GRVTITADKSTSTAYMELSSLRSEDTAVYYC

hcv23_GGCTGGTAGGACGAAA-1

GGTF....STYAISWVRQAPGQGLEWMGGIIPI..FGTANYAQKFQ.GRVTITADKSTSTAYMELSSLRSEDTAVYYC

hcv23_GGGACCTCATCACGTA-1

GGTF....STYAISWVRQAPGQGLEWMGGIIPI..FGTANYAQKFQ.GRVTITADKSTSTAYMELSSLRSEDTAVYYC

hcv23_GGTGTTACAGGGCATA-1

GGTF....STYAISWVRQAPGQGLEWMGGIIPI..FGTANYAQKFQ.GRVTITADKSTSTAYMELSSLRSEDTAVYYC

hcv23_GTATCTTGTATAGGTA-1

GGTF....STYAISWVRQAPGQGLEWMGGIIPI..FGTANYAQKFQ.GRVTITADKSTSTAYMELSSLRSEDTAVYYC

hcv23_GTGAAGGAGGAGCGAG-1

GGTF....STYAISWVRQAPGQGLEWMGGIIPI..FGTANYAQKFQ.GRVTITADKSTSTAYMELSSLRSEDTAVYYC

hcv23_TAAGTGCCAGGTCTCG-1

GGTF....STYAISWVRQAPGQGLEWMGGIIPI..FGTANYAQKFQ.GRVTITADKSTSTAYMELSSLRSEDTAVYYC

hcv23_TAGACCAAGTTGAGAT-1

GGTF....STYAISWVRQAPGQGLEWMGGIIPI..FGTANYAQKFQ.GRVTITADKSTSTAYMELSSLRSEDTAVYYC

hcv23_TGAGCATAGAAACCAT-1

GGTF....STYAISWVRQAPGQGLEWMGGIIPI..FGTANYAQKFQ.GRVTITADKSTSTAYMELSSLRSEDTAVYYC

hcv23_AACTCTTTCGTCACGG-1

GGTF....SNYAISWVRQAPGQGLEWMGGIIPI..FGTANYAQKFQ.GRVTITADKSTSTAYMELSSLRSEDTAVYYC

hcv23_AAGTCTGCACTTCTGC-1

GGTF....SNYAISWVRQAPGQGLEWMGGIIPI..FGTANYAQKFQ.GRVTITADKSTSTAYMELSSLRSEDTAVYYC

hcv23_ACACTGAAGACGCAAC-1

GGTF....SNYAISWVRQAPGQGLEWMGGIIPI..FGTANYAQKFQ.GRVTITADKSTSTAYMELSSLRSEDTAVYYC

hcv23_AGCAGCCGTAGCCTAT-1

GGTF....SNYAISWVRQAPGQGLEWMGGIIPI..FGTANYAQKFQ.GRVTITADKSTSTAYMELSSLRSEDTAVYYC

hcv23_ATTCTACTCAGTCAGT-1

GGTF....SNYAISWVRQAPGQGLEWMGGIIPI..FGTANYAQKFQ.GRVTITADKSTSTAYMELSSLRSEDTAVYYC

hcv23_CGACTTCGTGCGAAAC-1

GGTF....SNYAISWVRQAPGQGLEWMGGIIPI..FGTANYAQKFQ.GRVTITADKSTSTAYMELSSLRSEDTAVYYC

hcv23_CTCAGAACAGGCGATA-1

GGTF....SNYAISWVRQAPGQGLEWMGGIIPI..FGTANYAQKFQ.GRVTITADKSTSTAYMELSSLRSEDTAVYYC

hcv23_GATCAGTGTAGTACCT-1

GGTF....SNYAISWVRQAPGQGLEWMGGIIPI..FGTANYAQKFQ.GRVTITADKSTSTAYMELSSLRSEDTAVYYC

hcv23_GTGCAGCAGAAACCTA-1

GGTF....SNYAISWVRQAPGQGLEWMGGIIPI..FGTANYAQKFQ.GRVTITADKSTSTAYMELSSLRSEDTAVYYC

hcv23_TCGAGGCGTCTTGATG-1

GGTF....SNYAISWVRQAPGQGLEWMGGIIPI..FGTANYAQKFQ.GRVTITADKSTSTAYMELSSLRSEDTAVYYC

hcv23_TCTATTGGTAAGAGGA-1

GGTF....SNYAISWVRQAPGQGLEWMGGIIPI..FGTANYAQKFQ.GRVTITADKSTSTAYMELSSLRSEDTAVYYC

hcv23_TTCTACAAGAGCTGCA-1

GGTF....SNYAISWVRQAPGQGLEWMGGIIPI..FGTANYAQKFQ.GRVTITADKSTSTAYMELSSLRSEDTAVYYC

hcv23_AACTTTCGTCTCGTTC-1

GGTF....SSYAISWVRQAPGQGLEWMGGIIPI..FGTANYAQKFQ.GRVTITADKSTSTAYMELSSLRSEDTAMYYC

hcv23_GCTTGAATCGGTTCGG-1

GGTF....SSYAISWVRQAPGQGLEWMGGIIPI..FGTANYAQKFQ.GRVTITADKSTSTAYMELSSLRSEDTAMYYC

hcv23_TAGGCATGTGCGCTTG-1

GGTF....SSYAISWVRQAPGQGLEWMGGIIPI..FGTANYAQKFQ.GRVTITADKSTSTAYMELSSLRSEDTAMYYC

hcv23_TGAAAGATCATTGCGA-1

GGTF....SSYAISWVRQAPGQGLEWMGGIIPI..FGTANYAQKFQ.GRVTITADKSTSTAYMELSSLRSEDTAMYYC

hcv23_TGGCGCACAAACTGCT-1

GGTF....SSYAISWVRQAPGQGLEWMGGIIPI..FGTANYAQKFQ.GRVTITADKSTSTAYMELSSLRSEDTAMYYC

hcv23_AAGGCAGGTTGGACCC-1

GGTF....SSYAISWVRQAPGQGLEWMGGIIPI..FGTANYAQKFQ.GRVTITADKSTSTVYMELSSLRSEDTAVYYC

hcv23_AGCGTATCAACGATCT-1

GDTF....SSYAISWVRQAPGQGLEWMGGIIPI..FGTANYAQKFQ.GRVTITADKSTSTAYMELSSLRSEDTAVYYC

hcv23_AGCGTCGCATGTAAGA-1

GGTF....SSYAISWVRQAPGQGLEWMGGIIPI..FGTANYAQKFQ.GRVTITADKSTSTAYMELSSLRYEDTAVYYC

hcv23_AGGTCATTCTTGTACT-1

GGTF....SSYAISWVRQAPGQGLEWMGGIIPI..FGTSNYAQKFQ.GRVTITADKSTSTAYMELSSLRSEDTAVYYC

hcv23_GTACGTAAGTGCGATG-1

GGTF....SSYAISWVRQAPGQGLEWMGGIIPI..FGTSNYAQKFQ.GRVTITADKSTSTAYMELSSLRSEDTAVYYC

hcv23_ATCATGGGTCGATTGT-1

GGTF....SSYAISWLRQAPGQGLEWMGGIIPI..FGTANYAQKFQ.GRVTITADKSTSTAYMELSSLRSEDTAVYYC

hcv23_CAGAATCGTCTAGCCG-1

GGTF....SSYAISWVRQAPGQGLEWMGGIIPM..FGTANYAQKFQ.GRVTITADKSTSTAYMELSSLRSEDTAVYYC

hcv23_GCACTCTAGAGCTTCT-1

GGTF....SSYAISWVRQAPGQGLEWMGGIIPM..FGTANYAQKFQ.GRVTITADKSTSTAYMELSSLRSEDTAVYYC

hcv23_GGGTCTGTCACCTTAT-1

GGTF....SSYAISWVRQAPGQGLEWMGGIIPM..FGTANYAQKFQ.GRVTITADKSTSTAYMELSSLRSEDTAVYYC

hcv23_TATGCCCCAGCTCCGA-1

GGTF....SSYAISWVRQAPGQGLEWMGGIIPM..FGTANYAQKFQ.GRVTITADKSTSTAYMELSSLRSEDTAVYYC

hcv23_TGGCTGGCATTGTGCA-1

GGTF....SSYAISWVRQAPGQGLEWMGGIIPM..FGTANYAQKFQ.GRVTITADKSTSTAYMELSSLRSEDTAVYYC

hcv23_CCAGCGATCTGCCAGG-1

GGTF....SSYAISWVRQAPGQGLEWMGGIIPI..FGTANYAQKFQ.GRVTITADKSTSTAYMELNSLRSEDTAVYYC

hcv23_CCCTCCTGTCTCCCTA-1

GGTF....SSYAISWVRQAPGQGLEWMGGIIPI..FGTANYAQKFQ.GRVTITADKSTSTAYMELNSLRSEDTAVYYC

hcv23_CGTTCTGCATGCATGT-1

GGTF....SSYAISWVRQAPGQGLEWMGGIIPI..FGTANYAQKFQ.GRVTITADKSTSTAYMELNSLRSEDTAVYYC

hcv23_GAGGTGATCAGAAATG-1

GGTF....SSYAISWVRQAPGQGLEWMGGIIPI..FGTANYAQKFQ.GRVTITADKSTSTAYMELNSLRSEDTAVYYC

hcv23_GTGTGCGCAGTCAGAG-1

GGTF....SSYAISWVRQAPGQGLEWMGGIIPI..FGTANYAQKFQ.GRVTITADKSTSTAYMELNSLRSEDTAVYYC

hcv23_TACGGATTCTTGCCGT-1

GGTF....SSYAISWVRQAPGQGLEWMGGIIPI..FGTANYAQKFQ.GRVTITADKSTSTAYMELNSLRSEDTAVYYC

hcv23_TGACGGCCAATGGATA-1

GGTF....SSYAISWVRQAPGQGLEWMGGIIPI..FGTANYAQKFQ.GRVTITADKSTSTAYMELNSLRSEDTAVYYC

hcv23_TTCGGTCGTATATGGA-1

GGTF....SSYAISWVRQAPGQGLEWMGGIIPI..FGTANYAQKFQ.GRVTITADKSTSTAYMELNSLRSEDTAVYYC

hcv23_CCATTCGCATTCCTCG-1

GGTF....SNYAISWVRQAPGQGLEWMGGIIPI..FGTANYAQKFQ.DRVTITADKSTNTAYMELSSLRSEDTAVYYC

hcv23_CGAGCCAAGACTTGAA-1

GGTF....SSYAISWVRQAPGQGPEWMGGIIPI..FGTANYAQKFQ.GRVTITADKSTSTAYMELSSLRSEDTAVYYC

hcv23_CGATCGGCATAGAAAC-1

GGTF....SSYAISWVRQAPGQGLEWMGGIIPI..FGTANYAQKFQ.GRVTITADKSTSTTYMELSSLRSEDTAVYYC

hcv23_CTCTACGCAGTCGATT-1

GGTF....SSYAISWVRQVPGQGLEWMGGIIPI..FGTANYAQKFQ.GRVTITADKSTSTAYMELSSLRSEDTAVYYC

hcv23_GACTAACTCCACGTGG-1

GGSF....SSYAISWVRQAPGQGLEWMGGIIPI..FGTANYAQKFQ.GRVTITADKSTSTAYMELSSLRSEDTAVYYC

hcv23_GATCTAGGTCCGTCAG-1

GGTF....SSYAISWVRQAPGQGLEWMGGIIPI..FATANYAQKFQ.GRVTITADKSTSTAYMELSSLRSEDTAVYYC

hcv23_TACGGGCCACATGTGT-1

GGTF....SSYAISWVRQAPGQGLEWMGGIIPI..FATANYAQKFQ.GRVTITADKSTSTAYMELSSLRSEDTAVYYC

hcv23_GCAGCCAAGACACTAA-1

GGTF....SSYAISWVRQAPGQGLEWMGGIIPI..FGTANYAQKFQ.GRVTITADKSTSTAYMELSSLRSEDTAVFYC

hcv23_GGGAGATAGGGTGTTG-1

GGTF....SSYAISWVRQAPGQGLEWMGGIIPI..FGTANYAQKFQ.GRVTITADKSTSTAYMELTSLRSEDTAVYYC

hcv23_TGAGCATCATGAGCGA-1

GGTF....SSYAISWVRQAPGQGLEWMGGIIPI..FGTANYAQKFQ.GRVTITADKSTSTAYMELTSLRSEDTAVYYC

hcv23_GGGCATCCATCCTTGC-1

GGTF....SSYAISWVRQAPGQGLEWMGGIIPI..FGTANYAQKFQ.GRVTITADKSTSTAYMELSSLKSEDTAVYYC

hcv23_GTACTCCAGAATAGGG-1

GGTF....SSYAISWVRQAPGQGLEWMGGIIPL..FGTANYAQKFQ.GRVTITADKSTSTAYMELSSLRSEDTAVYYC

hcv23_TACGGTACATAGAAAC-1

GGTF....NSYAISWVRQAPGQGLEWMGGIIPI..FGTANYAQKFQ.GRVTITADKSTSTAYMELSSLRSEDTAVYYC

hcv23_TAGTTGGCAATTGCTG-1

GGTF....SSYAISWVRQAPGQGLEWMGGIIPI..FGTANYAQKFQ.GRATITADKSTSTAYMELSSLRSEDTAVYYC

hcv23_TTATGCTAGAATTGTG-1

GGTF....SSYAISWVRQAPGQGLEWMGGIIPI..FGTANYAQKFQ.GRVTITADKSTSTAYMELSSLRSDDTAVYYC

hcv23_TTTATGCAGGTAGCTG-1

GGTF....SSYAISWVRQAPGRGLEWMGGIIPI..FGTANYAQKFQ.GRVTITADKSTSTAYMELSSLRSEDTAVYYC

hcv49_ACGGAGAAGCTAGTGG-1

GGTF....STYAISWVRQAPGQGLEWMGGIIPI..FGTANYAQKFQ.GRVTITADKSTSTAYMELSSLRSEDTAVYYC

hcv49_ACTGATGGTGCCTGTG-1

GGTF....STYAISWVRQAPGQGLEWMGGIIPI..FGTANYAQKFQ.GRVTITADKSTSTAYMELSSLRSEDTAVYYC

hcv49_AGTGGGATCGCCAGCA-1

GGTF....STYAISWVRQAPGQGLEWMGGIIPI..FGTANYAQKFQ.GRVTITADKSTSTAYMELSSLRSEDTAVYYC

hcv49_ATTACTCAGGAGTCTG-1

GGTF....STYAISWVRQAPGQGLEWMGGIIPI..FGTANYAQKFQ.GRVTITADKSTSTAYMELSSLRSEDTAVYYC

hcv49_CAACCTCGTTCGCGAC-1

GGTF....STYAISWVRQAPGQGLEWMGGIIPI..FGTANYAQKFQ.GRVTITADKSTSTAYMELSSLRSEDTAVYYC

hcv49_CATATGGGTCTAACGT-1

GGTF....STYAISWVRQAPGQGLEWMGGIIPI..FGTANYAQKFQ.GRVTITADKSTSTAYMELSSLRSEDTAVYYC

hcv49_CCTCTGAAGCAACGGT-1

GGTF....STYAISWVRQAPGQGLEWMGGIIPI..FGTANYAQKFQ.GRVTITADKSTSTAYMELSSLRSEDTAVYYC

hcv49_CGGACGTTCTCGATGA-1

GGTF....STYAISWVRQAPGQGLEWMGGIIPI..FGTANYAQKFQ.GRVTITADKSTSTAYMELSSLRSEDTAVYYC

hcv49_CTCACACTCGTTGCCT-1

GGTF....STYAISWVRQAPGQGLEWMGGIIPI..FGTANYAQKFQ.GRVTITADKSTSTAYMELSSLRSEDTAVYYC

hcv49_CTTAACTTCACTTATC-1

GGTF....STYAISWVRQAPGQGLEWMGGIIPI..FGTANYAQKFQ.GRVTITADKSTSTAYMELSSLRSEDTAVYYC

hcv49_GGATGTTCAAGTACCT-1

GGTF....STYAISWVRQAPGQGLEWMGGIIPI..FGTANYAQKFQ.GRVTITADKSTSTAYMELSSLRSEDTAVYYC

hcv49_GTAGGCCAGTAGTGCG-1

GGTF....STYAISWVRQAPGQGLEWMGGIIPI..FGTANYAQKFQ.GRVTITADKSTSTAYMELSSLRSEDTAVYYC

hcv49_GTTAAGCAGCGTCTAT-1

GGTF....STYAISWVRQAPGQGLEWMGGIIPI..FGTANYAQKFQ.GRVTITADKSTSTAYMELSSLRSEDTAVYYC

hcv49_TACTCATAGTGGACGT-1

GGTF....STYAISWVRQAPGQGLEWMGGIIPI..FGTANYAQKFQ.GRVTITADKSTSTAYMELSSLRSEDTAVYYC

hcv49_TAGCCGGAGTCAAGGC-1

GGTF....STYAISWVRQAPGQGLEWMGGIIPI..FGTANYAQKFQ.GRVTITADKSTSTAYMELSSLRSEDTAVYYC

hcv49_TATGCCCGTAAGGGCT-1

GGTF....STYAISWVRQAPGQGLEWMGGIIPI..FGTANYAQKFQ.GRVTITADKSTSTAYMELSSLRSEDTAVYYC

hcv49_TCAGCAAGTGTGACGA-1

GGTF....STYAISWVRQAPGQGLEWMGGIIPI..FGTANYAQKFQ.GRVTITADKSTSTAYMELSSLRSEDTAVYYC

hcv49_TCAGCTCAGTCCGTAT-1

GGTF....STYAISWVRQAPGQGLEWMGGIIPI..FGTANYAQKFQ.GRVTITADKSTSTAYMELSSLRSEDTAVYYC

hcv49_TGTATTCTCTGTTGAG-1

GGTF....STYAISWVRQAPGQGLEWMGGIIPI..FGTANYAQKFQ.GRVTITADKSTSTAYMELSSLRSEDTAVYYC

hcv49_ACGGGTCCATGCAATC-1

GGTF....SNYAISWVRQAPGQGLEWMGGIIPI..FGTANYAQKFQ.GRVTITADKSTSTAYMELSSLRSEDTAVYYC

hcv49_CCAATCCGTACAGTGG-1

GGTF....SNYAISWVRQAPGQGLEWMGGIIPI..FGTANYAQKFQ.GRVTITADKSTSTAYMELSSLRSEDTAVYYC

hcv49_CGCGTTTCAGACGCCT-1

GGTF....SNYAISWVRQAPGQGLEWMGGIIPI..FGTANYAQKFQ.GRVTITADKSTSTAYMELSSLRSEDTAVYYC

hcv49_GAAGCAGAGATATGCA-1

GGTF....SNYAISWVRQAPGQGLEWMGGIIPI..FGTANYAQKFQ.GRVTITADKSTSTAYMELSSLRSEDTAVYYC

hcv49_GAATGAAAGAAAGTGG-1

GGTF....SNYAISWVRQAPGQGLEWMGGIIPI..FGTANYAQKFQ.GRVTITADKSTSTAYMELSSLRSEDTAVYYC

hcv49_GGATTACAGCTAACTC-1

GGTF....SNYAISWVRQAPGQGLEWMGGIIPI..FGTANYAQKFQ.GRVTITADKSTSTAYMELSSLRSEDTAVYYC

hcv49_GGCGACTTCTCAAACG-1

GGTF....SNYAISWVRQAPGQGLEWMGGIIPI..FGTANYAQKFQ.GRVTITADKSTSTAYMELSSLRSEDTAVYYC

hcv49_TCGGTAATCGCATGAT-1

GGTF....SNYAISWVRQAPGQGLEWMGGIIPI..FGTANYAQKFQ.GRVTITADKSTSTAYMELSSLRSEDTAVYYC

hcv49_TGGTTAGAGTGAACGC-1

GGTF....SNYAISWVRQAPGQGLEWMGGIIPI..FGTANYAQKFQ.GRVTITADKSTSTAYMELSSLRSEDTAVYYC

hcv49_TTCCCAGTCAAGCCTA-1

GGTF....SNYAISWVRQAPGQGLEWMGGIIPI..FGTANYAQKFQ.GRVTITADKSTSTAYMELSSLRSEDTAVYYC

hcv49_ACGGGTCTCAATCACG-1

GGTF....SSYAISWVRQAPGQGLEWMGGIIPI..FGTSNYAQKFQ.GRVTITADKSTSTAYMELSSLRSEDTAVYYC

hcv49_ACTGAGTTCCGAGCCA-1

GGTF....SSYAISWVRQAPGQGLEWMGGIIPI..FGTANYAQKFQ.GRVTITADKSTSTAYMELSSLRSEDTAMYYC

hcv49_GGGATGATCGTACGGC-1

GGTF....SSYAISWVRQAPGQGLEWMGGIIPI..FGTANYAQKFQ.GRVTITADKSTSTAYMELSSLRSEDTAMYYC

hcv49_GTATCTTAGTTAAGTG-1

GGTF....SSYAISWVRQAPGQGLEWMGGIIPI..FGTANYAQKFQ.GRVTITADKSTSTAYMELSSLRSEDTAMYYC

hcv49_TTGTAGGTCCACGAAT-1

GGTF....SSYAISWVRQAPGQGLEWMGGIIPI..FGTANYAQKFQ.GRVTITADKSTSTAYMELSSLRSEDTAMYYC

hcv49_AGGGATGTCAGGTTCA-1

GGTF....SSYAISWVRQAPGQGLEWMGGIIPI..FGTANYAQKFQ.GRVTITADKSTSTAYMELTSLRSEDTAVYYC

hcv49_CACACAACACCGAAAG-1

GGSF....SSYAISWVRQAPGQGLEWMGGIIPI..FGTANYAQKFQ.GRVTITADKSTSTAYMELSSLRSEDTAVYYC

hcv49_GACCAATGTGTAACGG-1

GGSF....SSYAISWVRQAPGQGLEWMGGIIPI..FGTANYAQKFQ.GRVTITADKSTSTAYMELSSLRSEDTAVYYC

hcv49_CACCAGGGTGCCTGGT-1

GGTF....NSYAISWVRQAPGQGLEWMGGIIPI..FGTANYAQKFQ.GRVTITADKSTSTAYMELSSLRSEDTAVYYC

hcv49_CAGGTGCGTGTGACCC-1

GGTF....SSYAISWVRQAPGQGLEWMGGIIPI..FGTANYAQKFQ.GRVTITADKSTSTAYMELSSLTSEDTAVYYC

hcv49_CCCTCCTCATAAAGGT-1

GGTF....SSYAISWVRQAPGQGLEWMGGIIPI..FGTANYAQKFQ.GRVTITADKSTSTAYMELSSLTSEDTAVYYC

hcv49_CCTTTCTGTAGAGCTG-1

GGTF....SSYAISWVRQAPGQGPEWMGGIIPI..FGTANYAQKFQ.GRVTITADKSTSTAYMELSSLRSEDTAVYYC

hcv49_CTACATTTCCCATTAT-1

GGTF....SSYAISWVRQAPGQGLEWMGGIIPI..FGTANYAQKFQ.GRVTITADKSTSTAYMELNSLRSEDTAVYYC

hcv49_GATCAGTCAGCGTAAG-1

GGTF....SSYAISWVRQAPGQGLEWMGGIIPI..FGTANYAQKFQ.GRVTITADKSTSTAYMELNSLRSEDTAVYYC

hcv49_GATCAGTTCAGGTAAA-1

GGTF....SSYAISWVRQAPGQGLEWMGGIIPM..FGTANYAQKFQ.GRVTITADKSTSTAYMELSSLRSEDTAVYYC

hcv49_GGCTCGATCTTGCATT-1

GGTF....SSYAISWVRQAPGQGLEWMGGIIPM..FGTANYAQKFQ.GRVTITADKSTSTAYMELSSLRSEDTAVYYC

hcv49_GCACATATCTCAACTT-1

GGTF....SSYAISWVRQAPGQGLEWMGGIIPI..FGTANYAQKFQ.GTVTITADKSTSTAYMELSSLRSEDTAVYYC

hcv49_GCGCAACGTTTGGGCC-1

GGTF....SSYAISWVRQAPGQGLEWMGGIIPI..FGTGNYAQKFQ.GRVTITADKSTSTAYMELSSLRSEDTAVYYC

hcv49_GGGCATCGTTGGAGGT-1

GGTF....SSYAISWVRQVPGQGLEWMGGIIPI..FGTANYAQKFQ.GRVTITADKSTSTAYMELSSLRSEDTAVYYC

hcv49_TTTGCGCGTTAAGAAC-1

GGTF....SNYAISWVRQAPGQGLEWMGGIIPI..FGTANYAQKFQ.DRVTITADKSTNTAYMELSSLRSEDTAVYYC

**References**

1. Weber T, Potthoff J, Bizu S, Labuhn M, Dold L, Schoofs T, et al. Analysis of antibodies from HCV elite neutralizers identifies genetic determinants of broad neutralization. Immunity. 2022;55(2):341-54 e7. Epub 20220105. doi: 10.1016/j.immuni.2021.12.003. PubMed PMID: 34990590.
